# A randomized cross-over study protocol to evaluate long-term gait training with a pediatric robotic exoskeleton outside the clinical setting in children with movement disorders

**DOI:** 10.1371/journal.pone.0304087

**Published:** 2024-07-08

**Authors:** Taylor M. Devine, Katharine E. Alter, Diane L. Damiano, Thomas C. Bulea

**Affiliations:** Rehabilitation Medicine Department, National Institutes of Health Clinical Center, Bethesda, Maryland, United States of America; Imperial College London, UNITED KINGDOM

## Abstract

Individuals with neuromuscular disorders display a combination of motor control deficits and lower limb weakness contributing to knee extension deficiency characterized by exaggerated stance phase knee flexion. There is a lack of evidence for long-term improvement of knee extension deficiency with currently available clinical treatment programs. Our previous work testing a wearable robotic exoskeleton with precisely timed assistive torque applied at the knee showed immediate increases in knee extension during walking for children with cerebral palsy, which continued to improve over an acute practice period. When we applied interleaved assistance and resistance to knee extension, we observed improvements in knee extension and increased muscle activation indicating the potential for muscle strengthening when used over time. There is a need for additional, high-quality trials to assess the impact of dosage, intensity and volume of training necessary to see persistent improvement in lower limb function for these patient populations. This randomized crossover study (ClinicalTrials.gov: NCT05726591) was designed to determine whether 12 weeks of overground gait training with a robotic exoskeleton outside of the clinical setting, following an initial in clinic accommodation period, has a beneficial effect on walking ability, muscle activity and overall motor function. Participants will be randomized to either complete the exoskeleton intervention or continue their standard therapy for 12 weeks first, followed by a crossover to the other study component. The primary outcome measure is change in peak knee extension angle during walking; secondary outcome measures include gait speed, strength, and validated clinical scales of motor function and mobility. Assessments will be completed before and after the intervention and at 6 weeks post-intervention, and safety and compliance will be monitored throughout. We hypothesize that the 12-week exoskeleton intervention outside the clinical setting will show greater improvements in study outcome measures than the standard therapy.

## Introduction

The standard of care for treating knee extension deficiency in neuromuscular disorders includes some combination of three approaches: surgical interventions, chemodenervation procedures (i.e. Botulinum toxin injections), and physical therapy. Controlled studies and interventional trials of available treatments indicate long-term improvement in knee extension deficiency remains variable. Surgical outcomes can be unpredictable and there is evidence for weakening and ambulatory decline in the long term [1, 2]. Botulinum toxin injections may result in a temporary or limited improvement in overactivity or muscle spasticity but do not address the primary problem or ameliorate long-term deficits [3, 4]. Botulinum toxin injections are generally used in tandem with physical therapy to optimize results. Physical therapy programs include strength training, such as progressive resistance training of knee extensors [5], or gait training either overground or treadmill-based. Gait training allows the child to be full weight bearing, to train with body weight support, or to be guided by robotic assistance thereby enabling the rehabilitation program to be tailored to each child’s unique needs. These methods have shown a range of results demonstrating effectiveness for children with cerebral palsy at improving gait speed [6] and mobility, reducing loads on the lower limbs to allow improved upright posture and increasing walking endurance as a result of overall improvement in gross motor function [7–10].

Within the category of device augmented therapies are robotic-assisted gait trainers (RAGT). These devices contrast with body-weight support treadmill training because they strap the user into a multi-joint (hip, knee and ankle) powered robotic system that can assist with walking in all or part of the gait cycle without requiring user or therapist driven assistance [11]. Early versions of RAGT were treadmill-based, which limited their useable environment to the clinical setting and does not accurately replicate the demands on the lower-extremities during overground walking [12]. Additionally, the device and treadmill belt will continue to operate regardless of user effort, so it can be difficult to determine whether a child is actively or passively engaged with the device [12]. A study of ambulatory children with bilateral cerebral palsy found decreased volition in motor control and muscle activation of the legs when walking with a robot-assisted gait trainer on the treadmill [13].

The challenges of treadmill-based RAGT has led to recent proliferation in wearable robotic exoskeleton technology for pediatric populations [14]. Exoskeletons offer modularity in design and implementation; some devices which target more affected individuals span the trunk and the legs, offering support at multiple joints and even can incorporate motorized walkers for support [15, 16]. Exoskeletons which span multiple joints can also adapt the assistance strategy to the individual user, and recent efforts have focused on advanced control approaches to achieve this goal [17–20]. Other devices target single joints such as the ankle [21] or knee [22–24] in ambulatory individuals who walk with a gait pathology such as knee extension deficiency or drop foot. Initial cohort studies of these technologies, performed mostly in clinical settings, demonstrate their effectiveness as assistive devices to improve gait biomechanics including increases in gait speed, step length, ankle plantarflexion, knee extension, hip extension, walking distance and reduction in metabolic energy cost [15, 25–27].

Whereas these results demonstrate that device augmented therapies may assist mobility, their promise as therapeutic technologies to improve long term function is less clear. Initial clinical studies have shown some potential for rehabilitation gains after exoskeleton-mediated gait training. For example, programs that utilize multi-joint exoskeletons to provide assistive forces to aid walking have shown increases in gait speed and step length [13, 15]. A two-phase clinical gait training program utilizing a multi-joint exoskeleton with partial body weight support, wherein the first phase focused on moving the limbs according to a target gait pattern while the second phase encouraged active participation via progressive reduction in assistance, showed improvements in strength and gait performance metrics in four children with CP [28]. An alternative approach is to utilize robotic devices to provide resistance during walking as form of training. A tethered pelvic-based system that applied downward force during treadmill walking resulted in improved step length and knee extension after 15 sessions (3 per week, 5 weeks) of training [29]. Other approaches that have provided targeted resistance to the ankle during treadmill walking using tethered [30] and untethered [31] approaches showed increases in preferred walking speed after the training period, as well as reduction in metabolic cost of transport, improved 6-minute walk test, and increased plantarflexor muscle activation [32]. While these findings demonstrate the potential for exoskeleton facilitated gait training to improve function, they have yet to be evaluated in randomized trials. Further, these studies utilized clinic-based training sessions, which do not fully leverage the wearable technology that can facilitate delivery of gait therapy in a range of settings, thereby increasing the therapy dosage and intensity.

To address these limitations in currently available therapies, we designed a novel wearable robotic exoskeleton for overground use in children with knee extension deficiency. The device applies extensor torque at precisely timed portions of the gait cycle to assist knee extension during walking. In early testing, we found the device encouraged volitional muscle activity assessed by increased surface EMG coincident with improved knee extension [33]. When applied to an observational cohort study of children with crouch gait from cerebral palsy under a previous, IRB approved protocol (#13-CC-0210) at the NIH Clinical Center, the device was found to be safe for overground use and effective at altering posture, gait biomechanics and muscle activity during use in a clinical lab setting [25, 26, 34]. The observed mean improvements in knee extension (13 degrees) were similar to those of invasive surgeries [1]. A range of responses to the exoskeleton were also observed in this initial cohort, motivating the pursuit of more-individual specific control strategies as well as developing hardware that was more powerful and untethered, which would expand its potential environment of use. A case study on a child with CP found that the second-generation NIH pediatric robotic exoskeleton, or P.REX, improved the subject’s walking mechanics during use. This new version of the device deployed an adaptive mechanism characterized by assistive torque applied proportionally to the individual’s estimated knee extension moment in stance phase of the gait cycle. These results suggested the potential to personalize control strategies to each user [23], which would allow the device to be tailored to the unique demands of subjects across neuromuscular disorders. The second-generation P.REX device also incorporated an optional functional electrical stimulation (FES) unit, which can deliver stimulation to the knee extensor muscles that is precisely timed to the gait cycle phase and synchronized with the robotic assistance or resistance [35].

Following these initial findings, a new, novel wearable robotic exoskeleton (NIH-Agilik) was developed with an expanded target population to include children with cerebral palsy, spina bifida, muscular dystrophy or incomplete spinal cord injury [36]. This device had a slimmer profile, was more power efficient, and incorporated a user-friendly interface to control the device settings, offering the potential to be applied outside of the clinical setting after initial safety and feasibility testing. The NIH-Agilik is capable of applying torque bidirectionally (i.e., extensor or flexor) to assist or resist knee movement. Torque profiles, which are defined by specifying the torque magnitude and direction and the ramp on and ramp off rate, can be independently set for five phases of the gait cycle: early, mid- and late stance and early and late swing phase, enabling the overall torque profile during the stride to be tailored to each individual user [36]. This capability broadens the exoskeleton’s use-case by providing a tool to leverage the benefits of strength training with the possibility to improve selective motor control by delivering precisely targeted resistance to challenge functional movement. Thus, whereas an exoskeleton that applies only assistive torques to make walking easier can have broad rehabilitative effects associated with increased walking duration and enhanced neuromuscular and cardiovascular activity, one that applies resistive torques can target specific muscle groups and/or functions for improvement, in this case knee extension during walking. Additionally, by interleaving exoskeleton provided assistive and resistive torques within and/or between strides, it is possible to extend this targeted rehabilitation approach to individuals who may be too weak or have too poor selective motor control to utilize an exoskeleton intervention that only resists movement. Indeed, this interleaved approach may be most suitable for children who have the least functional mobility and consequently are at the highest risk for losing the ability to walk.

We piloted an interleaved strategy with the NIH-Agilik exoskeleton that applied resistance to knee extension during late swing phase, which must be overcome by the user to complete each step, while also applying assistance to knee extension during early and mid-stance phase to provide antigravity support and maintain forward momentum [36]. This approach pursues the complementary goals of knee extensor strengthening to address the lower limb weakness and improved selective motor control by providing on-demand resistance during phases of active knee extension, while also leveraging the assistive torques to increase training duration. The immediate effects of this interleaved assistance and resistance control strategy on the biomechanics and neuromuscular activity of a single subject with cerebral palsy (GMFCS level III) were recently tested to establish initial feasibility of this strategy [36]. The control system displayed a high level of accuracy in providing appropriately timed torque to assist and resist knee extension during overground walking, resulting in immediate improvement in knee extension and simultaneous increase in knee extensor muscle activation, demonstrating the potential for strengthening after prolonged use [36].

Clinical and basic neuroscience suggests high intensity and high dosage training is necessary to achieve functional improvement via motor learning [37, 38] and the exoskeleton intervention being studied in this protocol leverages these key features in multiple ways. The exoskeleton applies resistance to knee extension during walking, which requires an intense and specific motor response to overcome, which itself can strengthen muscles and the synaptic connections required to activate them. Because the exoskeleton is wearable and can be used overground in multiple settings, the error-augmenting flexor torque can be delivered at a high dosage (hundreds or thousands of steps each day). It also elicits a desirable after-effect of increased knee extensor muscle activity, and potentially increased knee extension, that can then be reinforced on a regular and extended basis as the user walks without the exoskeleton between training sessions. Evidence for gait training with wearable robotics in community and home settings is extremely limited. Given the intervention’s combination of multiple motor learning principles, the NIH-Agilik’s safe and effective overground operation, and preliminary findings of an immediate benefit from NIH-Agilik’s use on biomechanics, neuromuscular activity and functional ability, there is strong merit for its investigation outside of the clinical setting. The purpose of this study is to evaluate safety, dosing and initial efficacy of a wearable exoskeleton for overground gait training in home or community environments over a 12-week training period.

## Materials and methods

### Objectives and endpoints

The overall goal of this protocol is to evaluate our intensive 12-week community-based exoskeleton training program. The International Classification of Functioning, Disability, and Health (ICF) provides a comprehensive framework for description and assessment of functional abilities split across four domains: body structures and functions, activities and participation, personal, and environmental factors [39, 40]. The ultimate goal of an intervention study is to improve activity and participation. Given this is analogous to a phase I study, our endpoints here aim to evaluate changes at the ICF level of function and activity, with the goal to provide an initial body of evidence to support more comprehensive studies of this and similar technologies in the future.

The primary objective is to evaluate the safety and effectiveness of a longitudinal community exoskeleton training program to improve crouch gait in children with CP or knee extension deficiency in children with MD, SB, or iSCI. To evaluate this objective, the primary endpoint is change in peak knee angle during midstance phase of walking (without the exoskeleton) between two time points: the initial and final outcome assessments. These assessments occur immediately before and after the 12-week community-based exoskeleton training period. A clinically significant improvement in peak knee extension will be considered here as greater than 10°, based on a study characterizing children with crouch gait as mild, moderate or severe in terms of 10° increments [41]. This change in peak knee extension will also be compared to the change measured between the assessments immediately before and after the 12-week standard therapy period as a secondary endpoint. This outcome is at the ICF level of body structure and function.

The secondary objectives at the ICF level of body structure and function are as follows.

1. To evaluate peak and overall volitional knee extensor muscle (vastii, rectus femoris) activation during walking. The associated endpoint is change in knee extensor muscle activation between two time points: the initial and final outcome assessments. This outcome will be assessed using surface EMG recorded during walking. Peak muscle activation will be measured as the greatest EMG activity within a stride, and overall knee extensor activation will be measured by area under the normalized EMG curve as in [26].
2. To evaluate change in knee extensor muscle strength following completion of the 12-week community robotic exoskeleton use. Knee extensor strength will be assessed using the Biodex dynamometer while seated with a knee angle of 60 degrees to measure the maximum isometric knee extension torque, between the initial and final outcome assessments.
3. To evaluate persistence of improvement in peak knee extension (primary endpoint) at six weeks post completion of robotic exoskeleton training.

The following secondary endpoints at the ICF level of activity will be evaluated.

1. The change in average gait speed during overground walking before and after robotic exoskeleton training. Gait speed will be assessed using two endpoints: walking overground using the Vicon Motion Capture system and the 6-minute walk test.
2. The improvement in gross motor function following robotic exoskeleton gait training. This will be measured using three validated functional instruments, the Timed Up-and-Go test [42], the GMFM-66 assessment [43] and the PEDI-CAT [44].

Like the primary endpoint, changes in the secondary endpoints from the exoskeleton intervention period will be compared with changes measured before and after the standard therapy period. We also will evaluate a number of exploratory objectives.

1. The safety and feasibility of the community-based exoskeleton gait training program assessed by recording adverse events and side effects, which will be split into two categories: (a) minor or anticipated side effects and (b) serious or unanticipated side effects. Feasibility will be assessed as a measure of compliance to study parameters, by determining whether the individual met the suggested dosage of device use while in the community.
2. We will evaluate the primary endpoint (peak knee extension at midstance) as a function of dosage by comparing this parameter across multiple time points: Baseline to Initial Assessment (signals completion of in-lab accommodation/training block), Baseline to Intermediate Assessment (signals 50% completion of community use block), Baseline to Final Assessment (signals completion of community use block), and Baseline to Follow-up Assessment (signals 6-weeks post completion of community use block).
3. We will evaluate changes in lower limb muscle spasticity after robotic exoskeleton gait training using the modified Ashworth scale and the Tardieu scale measured at two time points: the initial and final outcome assessment.

### Study design

**Fig 1:**
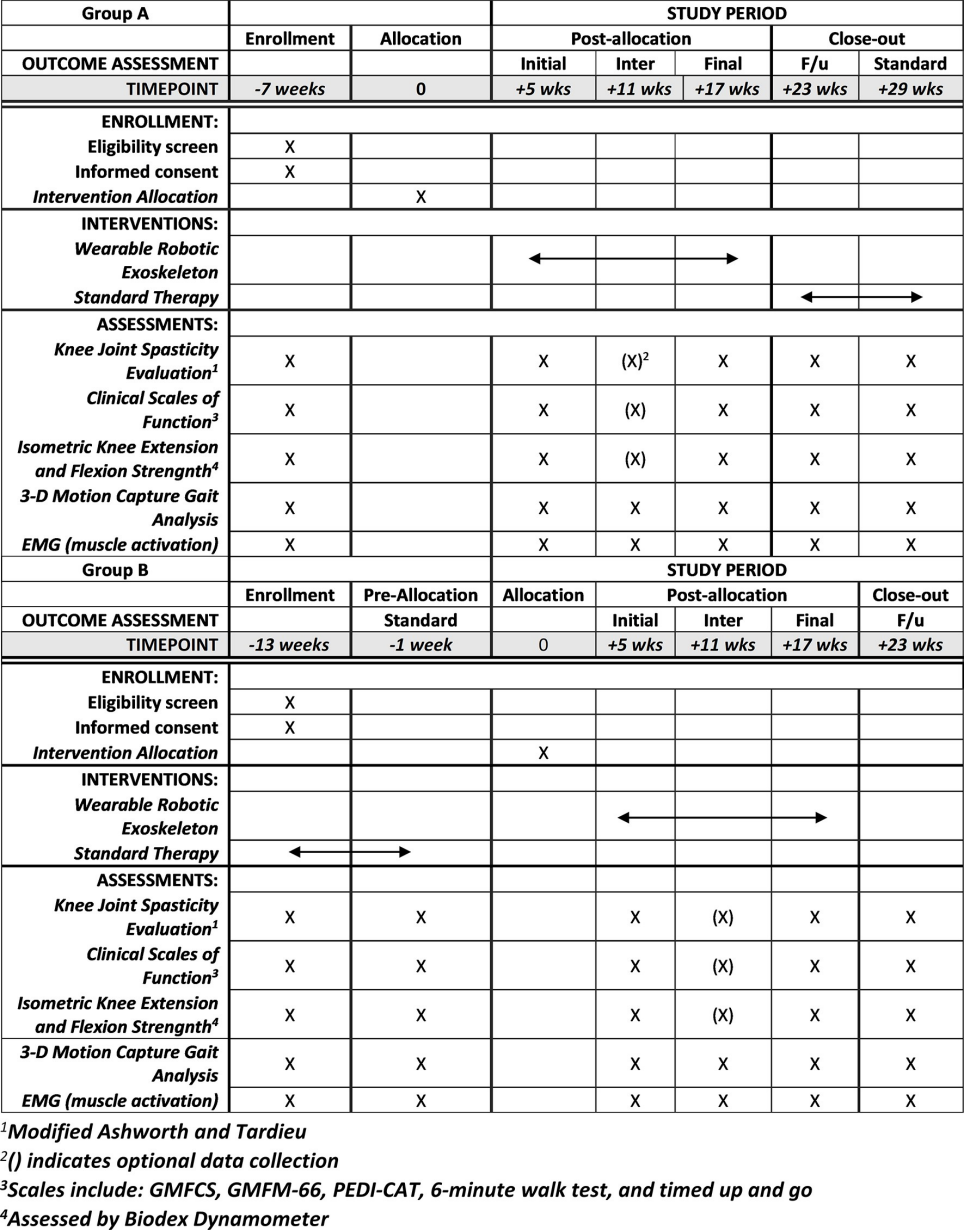
Enrollment, interventions and assessment chart for Group A and Group B.

**Fig 2:**
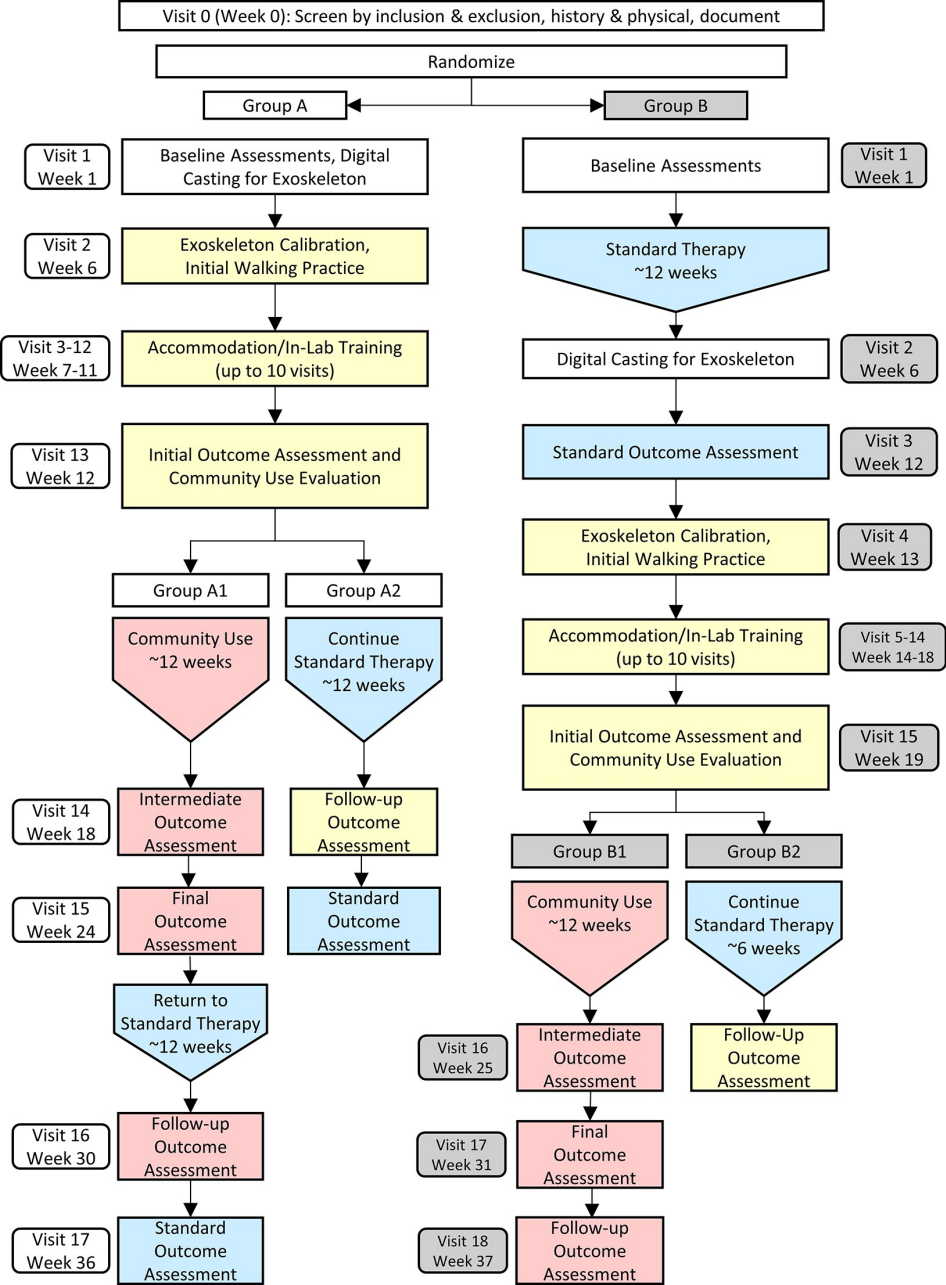
Schematic diagram of the randomized, cross-over protocol. Yellow indicates the 12-week block for exoskeleton set-up and in-lab accommodation. Pink indicates the 12-week block for community exoskeleton use, including a 6-week follow up period. Blue indicates the 12-week block for standard therapy in the community.

This study has been approved by the Institutional Review Board of the National Institutes of Health and is registered on clinicaltrials.gov (NCT05726591). It is a single site outpatient study with all data collection to occur at the National Institutes of Health Clinical Center, Rehabilitation Medicine Department, Neurorehabilitation and Biomechanics Section and is analogous to a phase I/II clinical trial. Interventions and up to six outcome assessments will be conducted according to the schedule outlined in Figs 1 and 2.

The design is structured as a randomized crossover study with two intervention arms: (i) exoskeleton intervention and (ii) standard therapy control (Fig 2). The crossover design was chosen to maximize participation by allowing all participants to receive the exoskeleton intervention. After enrollment, each participant will be randomized into one of two groups: Group A will receive the exoskeleton intervention first, followed by standard therapy control, while Group B will complete standard therapy control first before crossing over to receive the exoskeleton intervention. Continuation of the participant’s existing standard therapy was chosen as the control due to limited resources to administer a common program and because individuals in the target populations are often enrolled in a physical therapy regimen. All participants will complete an initial visit for exoskeleton fitting, setup, and calibration as well as multiple in-lab accommodation visits prior to starting the community-based exoskeleton intervention. An alternative option, if the participant is not able to complete the community use portion of the protocol for any reason, is to continue the intervention by completing a lab-based exoskeleton training consisting of 10 total visits, each with a target of 1-hour total of walking with the exoskeleton. The target intervention dosage in the community will be held constant across all subjects who participate at 1 hour/day, 5 days/week. Outcomes from individuals who complete the lab-based training will be analyzed separately from those who complete the community-based intervention. The maximum possible number of visits to NIH for completion of this study is 19. All visits will take no more than 4 hours to complete. There will be up to six outcome assessments to evaluate our primary, secondary and exploratory objectives.

### Study population

The target population is children between the age of 3 to 17 with crouch gait from cerebral palsy (CP) or knee extension deficiency from another neuromuscular disorder, including muscular dystrophy (MD), spina bifida (SB), or incomplete spinal cord injury (iSCI). We plan to specifically target children with these diagnoses because the associated gait pathology is known to progress toward loss of ability to walk with age, often as early as puberty [45]. Participants will be pooled by diagnosis; one for CP and one for other neuromuscular disorders. This design accounts for the differences in the underlying mechanism of gait pathology; subjects with CP walk in a crouched posture due to multiple impairments, including weakness, spasticity, over active muscles, motor control deficits [46], or some combination thereof, whereas patients with MD, SB or iSCI tend to present with gait pathology as a result of muscular weakness [47–49]. Each pool will complete the same study protocol but will be analyzed separately.

A recruitment flyer detailing study inclusion criteria and general information will be distributed via the NIH list-serv as well as shared with offices of clinical care providers, physicians or physical therapists in the area or displayed throughout hospitals, physicians’ offices or rehabilitation settings. We intend to also advertise the study on appropriate social media outlets (i.e. YouTube, Facebook or Twitter) and special interest groups within those sites through the NIH Office of Patient Recruitment. We may contact previous subjects who expressed interest in returning for more research participation. All prospective patients will be contacted by phone and/or email for initial screening of basic components for inclusion prior to being invited for visit 0 to complete all components of screening for inclusion.

All potential candidates and their parent/guardian will first provide informed assent/consent for the initial screening process. Following a determination of eligibility for inclusion in the protocol, the participant and their parent/guardian will then be asked to provide informed assent/consent for participation in the study. This study will follow NIH HRPP Policy 402 to ensure we provide sufficient information in an appropriate manner to each child subject to avoid undue influence or unnecessary risk while involved in research. We will require a signed consent document by the legal parent or guardian for each participant, with an assent signature line on the consent document for children age 14–17. There will be a separate assent document for children age 8–13. Children aged 6–7 will be asked to provide verbal assent for participation while children under the age of 6 years will not be required to give verbal assent because they typically are not able to fully understand the scope of scientific research.

To be eligible to participate in this study, the following inclusion criteria must be met:

- Age 3 to 17 years old.
- Have decreased active knee extension, from a diagnosis of cerebral palsy, muscular dystrophy, spina bifida or incomplete spinal cord injury
- Knee joint passive range of motion of at least 25 degrees in the sagittal plane (knee extension/flexion) assessed with hip extended in supine position. The presence of a hamstring contracture as assessed by straight leg raising test does not limit ability to participate in the study as long as range of motion requirement is met.
- Ankle joint range of motion of at least 15 degrees in the sagittal plane (dorsi-plantar flexion) with the foot in neutral alignment.
- A measured foot-thigh angle of -15 to 30 degrees in prone position.
- Ability to walk at least 10 feet without stopping with or without a walking aid.

If any of the following criteria are met, the individual would be excluded from participation:

- Any neurological, musculoskeletal or cardiorespiratory injury, health condition, or diagnosis other than cerebral palsy, muscular dystrophy, spina bifida, or incomplete spinal cord injury that would affect the ability to walk as directed with the robotic exoskeleton.
- A history of uncontrolled seizure in the past year.
- Pregnancy. All participants who are able to become pregnant will complete a urine test at the initial screening visit and in the case of a positive test, the participant will be excluded from participation. Further monitoring will rely on self-reporting of interruption in menstruation that would require re-testing for pregnancy at the next visit.
- Any acute cardiopulmonary condition which limits exercise to less than 60 minutes per session or less than 5 days per week.

Participants are free to withdraw their participation in the study at any time upon request. It is also possible for an investigator to withdraw a participant from the study intervention at their own discretion, or if the subject were to experience an adverse event or if their disease progressed to a point requiring discontinuation of study intervention. When a participant terminates participation in the study, effort will be made to complete a final evaluation visit within approximately 3 weeks of the last exoskeleton walking visit.

### Intervention and dosage

**Fig 3:**
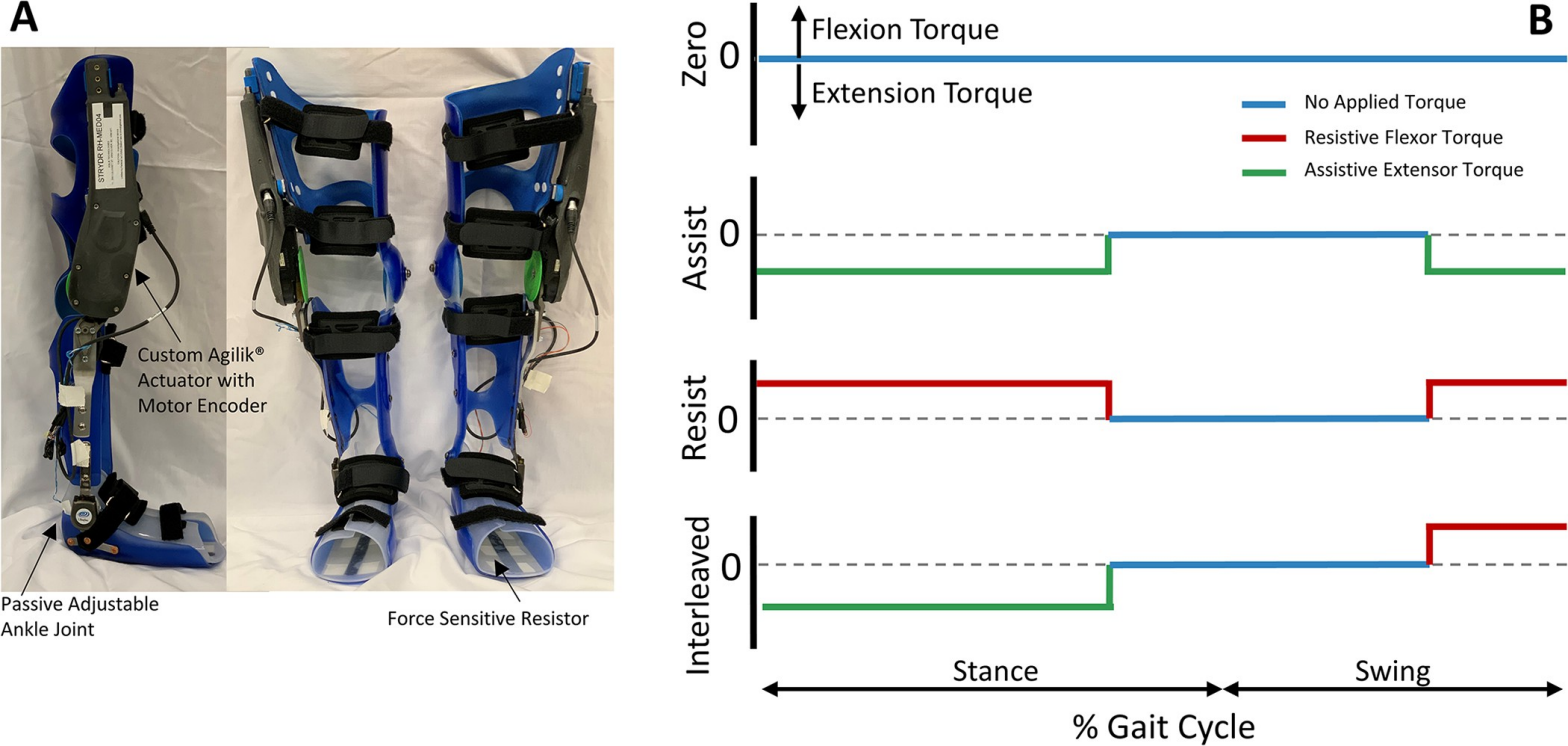
The NIH-Agilik exoskeleton. (A) Components of the NIH-Agilik exoskeleton, which includes orthotics customized to each user, an actuated knee joint, and a passive adjust ankle joint. (B) Example torque profiles applied over the gait cycle (heel-strike to heel-strike) for each exoskeleton operational mode.

The intervention used in this study is a wearable robotic exoskeleton consisting of two custom orthotic braces, one for each limb, with each having a single actuated degree of freedom at the sagittal plane knee joint (flexion/extension direction) and a passive, adjustable joint at the ankle (Fig 3A). Each limb is powered at the knee joint and controlled by the embedded electronics and sensors which can be optimized to each participant’s gait pattern. Functional electrical stimulation (FES) can be optionally included by placing surface electrodes over the targeted muscles (knee extensors) on the lower limbs. The FES electronics are integrated with the embedded control system of the exoskeleton enabling stimulation to be synchronized with the robotically delivered torque at the knee.

The exoskeleton is composed of multiple parts with final assembly in our facility. A 3-dimensional digital scan of each participant’s legs is taken with a Structure Sensor Pro connected to an iPad tablet and sent to an orthotics manufacturer (Ultraflex Systems, Inc.) who fabricates the custom thermoplastic shells with attached aluminum uprights on the lateral side of the limb to which the passive ankle joint and Agilik® actuator (Bionic Power, Inc.) are mounted. The upright on the lower leg includes a height adjustment that allows the distance between the ankle and knee joint centers to be expanded by up to 3 inches to account for participant growth during the study. The orthoses, actuator, and under foot force sensitive resistor (FSR, Interlink Electronics, Westlake Village, CA) are assembled to form the robotic exoskeleton by the NIH study team.

There are three available exoskeleton control strategies (Fig 3B): one that provides synergistic assistive torque to knee extension during stance and swing phase (Assist Mode), one that applies torque to oppose knee extension during stance and swing (Resist Mode), and one that provides knee extension assistance during stance phase but resists knee extension during late swing (Interleaved Mode). Each mode can be used independently or can be paired with knee extensor FES. The ultimate intervention goal is to increase volitional function following completion of the community intervention training, so incorporating resistive training will be prioritized to aid in rehabilitation of knee extension deficiency [37, 38]. We will secondarily establish at least one assistive-only mode as an option for the participant if they cannot complete the prescribed dosage with the resistive or interleaved modes.

A baseline gait analysis walking with their usual assistive devices (i.e. AFO, walker, crutches) using motion capture and EMG, will be used to set the initial parameters of the device after fabrication, which typically takes 4–6 weeks. After this time, the participant will return to the lab for fit-testing, calibration and tuning of the exoskeleton control parameters. As part of this process, the primary exoskeleton mode will initially be set to the Resist Mode, which applies resistance to knee extension during stance and swing phase. In this tuning phase, the torque profile is customized to each participant by specifying the magnitude and ramp rate of the torque provided during each of the five gait phases (Fig 3B). If the participant is unable to ambulate in this mode, the primary mode will revert to the Interleaved Mode, which assists extension during stance and resists it during swing. Additionally, a secondary Assist Mode of operation will be set for each participant. Prior to taking the device home for the community intervention training, each participant and their caregiver must complete an up to 10 visit accommodation period for walking in the primary and secondary modes. These sessions will consist of walking overground across various surfaces and environments at the hospital, including tile, laminate and carpet indoors, as well as outdoor pathways with inclines and declines and concrete sidewalks. Clinical observation by study physical therapists, as well as analysis of exoskeleton sensor data during each type of walking, will be completed to ensure safe operation and optimal exoskeleton controller performance across the conditions and modes. The accommodation period also includes education of the participant and parent/caretaker on how to don/doff the exoskeleton, how to operate its software to turn it on/off, and how to charge the battery. The participant will be allowed to take the device into the community for 12 weeks of intervention if the user, their caregiver and the study team members are comfortable with their competency using the device. All control parameters will be set before the user takes the device home, so they will not be required to tune the device outside of the clinical setting. The participant will receive a laptop computer with software that enables the user to switch between operational modes as well as complete virtual telehealth visits with the study team. The custom software also enables the user or study team to collect sensor data from the exoskeleton during the weekly virtual visits, which will be used to verify the exoskeleton operation each week and make any changes to the controller parameters, if necessary. The participant will be instructed to utilize their primary mode for exoskeleton training, with a target of 1 hour/day for 5 days/week. They will be permitted to use the secondary Assistive Mode, if desired, after completing the primary mode training for the day, or on days when training is not prescribed. Participants are asked to complete a log of their exoskeleton use during the intervention period, including the number of minutes and mode of operation for each day. During the community intervention period the study team will set up weekly check-ins with the participant. These sessions are utilized to confirm there are not fit or operational issues with the exoskeleton and to review participant compliance with training procedures and provide encouragement to continue or enhance compliance if necessary. If any technical issues with the device were to arise, the user will be instructed to contact the study team immediately rather than wait for the weekly check-in appointment. If repairs or maintenance to the exoskeleton are necessary, the study team will travel to the participant to complete them as soon as possible.

### Study assessments and outcomes

All participants will complete the same study procedures and assessments (S1 Table), only varying in order of completion by Group (Fig 2). Each assessment visit will include collection of all outcome measures as indicated in Table 1. For Group A, assessments will be performed in the following order: at baseline (upon enrollment), initial outcome assessment (after in lab accommodation), intermediate outcome assessment (6 weeks after start of community exoskeleton intervention), final outcome assessment (12 weeks after start of community exoskeleton intervention), follow up outcome assessment (6 weeks after completing community exoskeleton intervention), and standard outcome assessment (12 weeks after starting standard therapy control). For Group B, assessments will be performed as follows: baseline assessment, standard outcome assessment, initial outcome assessment, intermediate outcome assessment, final outcome assessment, and follow up outcome assessment.

**Table 1:** Outcome measures by ICF domain and data collection timepoints.

Outcome Measure	ICF Domain^1^	Baseline Assessment	Exoskeleton Intervention Assessments				Standard Outcome Assessment
			Initial	Intermediate	Final	Follow-Up	
Peak knee extension (deg)	Body structure	X	X	X	X	X	X
Knee extension at initial contact (deg)	Body structure	X	X	X	X	X	X
Step length (m)	Function	X	X	X	X	X	X
Gait speed (m/s)	Function	X	X	X	X	X	X
Knee moment in stance phase (Nm)	Function	X	X	X	X	X	X
Peak activation of knee extensor (mV/mV)	Function	X	X	X	X	X	X
Mean knee extensor muscle activation (mV/mV)	Function	X	X	X	X	X	X
Gross motor function classification system (GMFCS, I-V)	Function	X	X	(X)^2^	X	X	X
Pediatric evaluation of disability inventory–computer adaptive test (PEDI-CAT, scaled 20–80)	Activity	X	X	(X)	X	X	X
Gross motor function measure (GMFM-66, scored 0–198)	Activity	X	X	(X)	X	X	X
6-minute walk test (6MWT, m)	Function	X	X	(X)	X	X	X
Timed up and go (s)	Function	X	X	(X)	X	X	X
Knee extensor spasticity (Modified Ashworth scale, 0–4)	Body structure	X	X	(X)	X	X	X
Knee extensor spasticity (Tardieu scale, deg)	Body structure	X	X	(X)	X	X	X
Knee extensor isometric strength (Nm)	Function	X	X	(X)	X	X	X
Knee flexor isometric strength (Nm)	Function	X	X	(X)	X	X	X
Quebec User Evaluation of Satisfaction with Assistive Technology 2.0 (QUEST 2.0)	Activity				X		

^1^Domains as categorized by International classification of functioning, disability and healthy children and youth version [40].

^2^() indicates assessments are optional at the indicated visit.

### Hypotheses

We hypothesize that regular use of a robotic exoskeleton in the community setting will lead to functional improvement in overground walking without the exoskeleton by (i) increasing peak knee extension angle, (ii) improving overground gait speed, and (iii) improving knee extensor (vastus lateralis and rectus femoris) muscle activation and strength. We hypothesize that these improvements will be greater than changes measured before and after the 12-week standard therapy period. We further hypothesize that community-based exoskeleton training will increase motor function and mobility, which will be assessed using clinically validated scales of activity and mobility. Finally, we hypothesize that observed functional improvements as a result of the 12-week community intervention training period will persist following completion of the training, as measured during the follow-up assessment.

### Statistical considerations

Sample size was determined from pilot data collected during a prior, in-lab protocol performed at NIH Clinical Center (#13-CC-0210) using the same exoskeleton. We used the targeted mean improvement in peak knee extension during midstance, which was approximated as 10.0 degrees, with an observed standard deviation of ± 3 degrees (n = 3) and a safety factor of 4 given the previously observed variability in response to our exoskeleton [26], resulting in a computed effect size of 0.86. At a significance level of 0.05 and 90% power we estimate that 13 participants within each subject pool is required based on power analysis for a paired t-test. We estimated about 80% of enrolled participants would complete the study through the final outcome assessment, and additionally considered 25% would complete the protocol, but elect out of the community use portion. In order to ensure 13 participants have completed the required study components, we determined at most 22 participants would need to be recruited for each subject pool, setting the maximum number of participants as 44.

Our continuous data outcomes will be summarized using descriptive statistics and presented as means with standard deviation. We will summarize our categorical data as a range with percentages. The primary endpoint, peak knee angle at midstance, will be analyzed using a per-protocol analysis to include all participants who completed at least 80% (9 weeks) of the study intervention in the community using a paired t-test between the initial and final outcome assessments. Similarly, a paired t-test will be utilized to compare change in the primary endpoint of peak knee extension with the change observed over the 12-week standard therapy block. Given the potential for carryover effects, this secondary outcome will only be assessed using data from the first intervention period from each group. We will test for normality and if not found, we will use the signed rank test instead of the paired t-test. Given the potential heterogeneity within each participant pool, we anticipate the possibility for a wide range of responses in the endpoints following the intervention. To address this potential variability, we will also perform a post-hoc linear regression analysis to assess potential effects of age, weight/height, and mobility (characterized by functional scale GMFCS I-IV) on each endpoint. We will perform an interim analysis of the primary endpoint when we have reached 50% of the estimated recruitment number to evaluate the effect and the power and revise the total enrollment number if indicated.

The same statistical procedures will be used for the secondary outcome measures and exploratory outcome measures that evaluate continuous data between multiple time points. We will report the 95% confidence intervals, in addition to the mean difference and statistical significance of that difference, for each endpoint. For the exploratory analysis of dosage effect across all four outcome assessments, we will use a repeated measures ANOVA.

### Data management, safety considerations and risks-benefit analysis

The principal investigator (PI) will oversee all data and safety monitoring for this protocol. The research study staff and the PI will meet regularly when participants are actively to discuss each subject and evaluate the safety of study procedures, including review of any adverse events reported to plan for necessary procedural changes in response. A board-certified, NIH RMD physician will regularly review clinical data with the PI to evaluate for patient safety issues. All subjects enrolled in the study will be informed of their ability to discontinue participation at any time.

The NIH-Agilik device used in this study is an approved Class I device under FDA regulations (Regulation Number 890.3475; Establishment Registration & Device Listing (fda.gov)) and is of minimal risk. The orthotic braces used in the exoskeleton are custom fabricated for each individual, minimizing the potential for discomfort or pain during use. The orthosis fit will be inspected during the in-lab accommodation sessions by a physical therapist or physician, and participants and their parent/caretaker will be instructed on how to self-monitor for skin issues during these sessions as well. If any skin issues arise, the intervention will be paused until an appropriate adjustment can be completed. Engineers and clinicians will work closely with the user and their caregiver during the in-lab accommodation period to be sure the settings of the device, including the magnitude of applied torque, are safe and tolerable. The exoskeleton control system includes settings that limit torque application to a specified knee range of motion and the device is also equipped with an emergency stop button that the participant can press at any time discomfort is felt. Each participant will be evaluated individually by the study physician, physical therapist and PI before taking the device home to ensure they can safely operate the exoskeleton outside the clinical setting. There are potential physical side effects of the exoskeleton intervention, including muscle soreness or fatigue. Serious, and unanticipated, side effects would include soft tissue injury, bone fractures, shortness of breath or cardiovascular/respiratory events. We will monitor participants through weekly (minimum) check-ins during the intervention for these anticipated and unanticipated side effects, including monitoring, determination, and reporting of any adverse events.

The findings from our previous protocol show the potential benefit of the robotic exoskeleton as an assistive device to increase knee extension, increase knee range of motion, improve step length and improve muscle activity [22, 25, 26, 33, 34]. In addition to our research, other studies have shown the potential for lower limb gait training devices to be beneficial for our target population. The interleaved assistance and resistance exoskeleton mode has also been piloted, with results suggesting its safety and potential to address lower limb muscular weakness when used in the long-term [36]. Strength training study designs targeting lower extremity muscles are known to be beneficial for improving gross motor function and gait speed in children with cerebral palsy [50]. Our control strategy combines strength training of selective neuromuscular control with assistive torque application to maintain functional ambulation. The target population is ambulatory and likely familiar with other gait training therapies, making them predisposed to the demands on the lower-limbs while using assistive devices. The in-lab accommodation period will ensure user ability to operate the device without study team supervision. We anticipate a possible therapeutic effect on neuromuscular and gait biomechanics at completion of this protocol. Therefore, the potential benefits outweigh the minimal risks associated with participation in this study. Participation in this study will add to the body of knowledge on wearable devices and their application as gait trainers outside of the clinical setting, which has extended benefits to future rehabilitative technology for this target population.

### Status and timeline of study

The study protocol has been approved by the Institutional Review Board of the National Institutes of Health. Participant recruitment was initiated in June 2023 and informed consent and assent will be obtained from all participants prior to enrollment. We expect to enroll 1 participant per month. This goal would allow us to reach our maximum enrollment within 4–5 years, with an additional year for final completion of study procedures and follow-up, making the total study duration 6 years.

## Results

### Pilot data to demonstrate intervention proof of principle

Under our previous safety and efficacy protocol (#13-CC-0210), six participants completed ten visits to the Neurorehabilitation and Biomechanics Research Section at the NIH Clinical Center, which included eight sessions walking in the same wearable robotic exoskeleton that will be used for this protocol. All participants and their guardians completed informed assent and consent, respectively. The data presented here are for a single participant with crouch gait from CP at the mid-study data collection session and are included to demonstrate the intervention proof of principle. All three possible operational modes of the exoskeleton are shown: Assist mode, which assisted knee extension during mid-stance and late swing phase; Resist mode, which resisted knee extension with a flexor torque during mid-stance and late swing; and Interleaved mode, which provided knee extension assistance during midstance and resistance during late swing. The participant (male, 7 years old, 109 cm, 18.1 kg, GMFCS III) tolerated all modes well.

**Fig 4:**
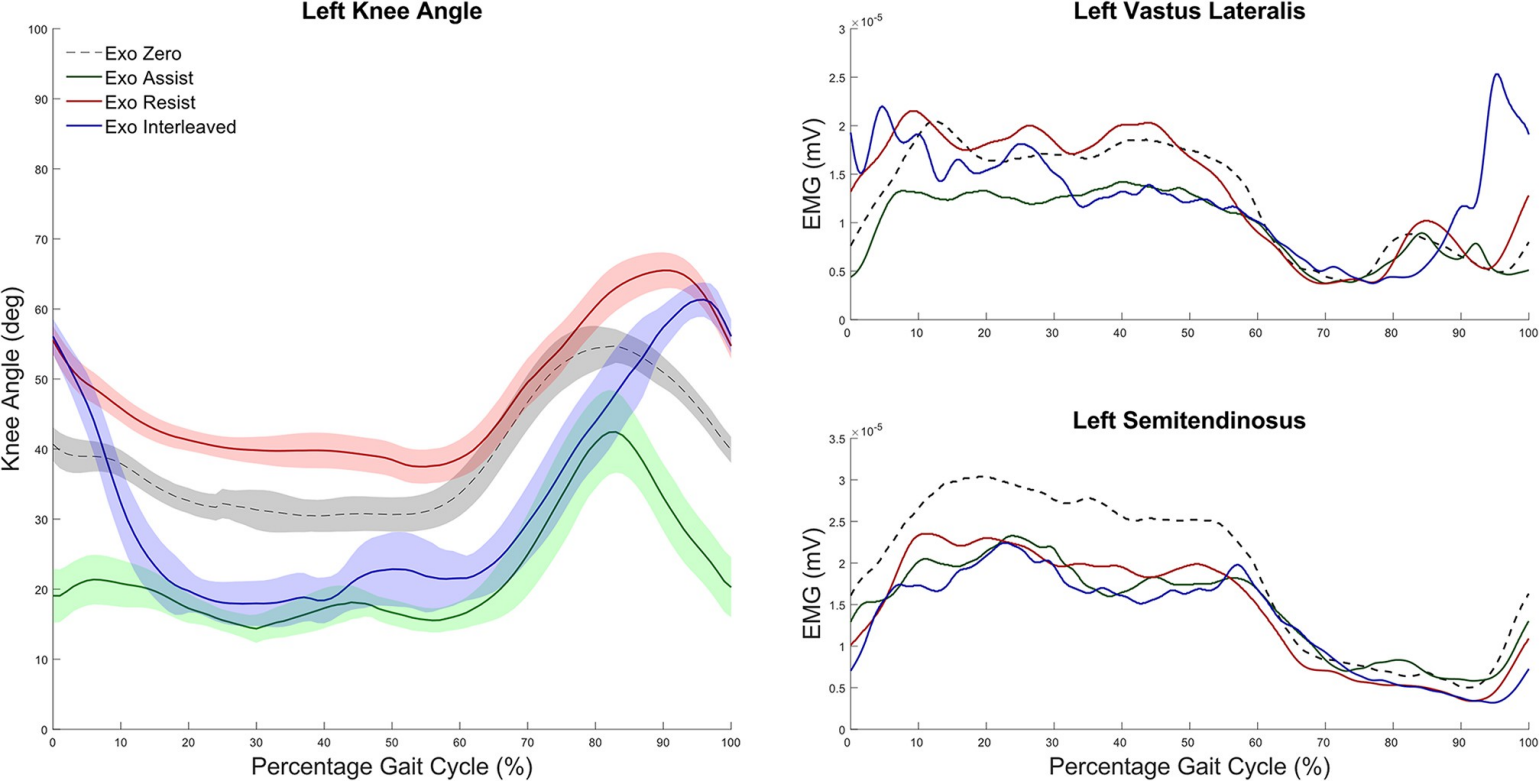
Pilot results showing the effect of the NIH-Agilik exoskeleton on gait biomechanics. (A) Average knee angle across the gait cycle during over ground walking with the exoskeleton using the Zero Mode (black), Assist Mode (green), Resist Mode (red) and Interleaved Mode (blue) for a single participant with crouch gait from CP. Shaded areas indicate 1 standard deviation around the mean. (B) Average vastus lateralis (top) and semitendinosus (bottom) muscle activation for the same strides.

The kinematics plot (Fig 4A) shows improvement in knee angle from baseline (black) while using the Assist mode (green) during overground walking, compared to a slight worsening of crouch in the Resist mode (red). The Interleaved mode demonstrates the combination of these effects, whereby stance phase knee extension is improved due to the assistance but late swing phase extension is diminished by the resistance. Surface EMG data collected from knee extensor (vastus lateralis, VL) and knee flexor (semitendinosus, ST) muscle activation patterns reveal an overall reduction in the Assist mode (Fig 4B), which is expected due to the improved posture which reduces the antigravity demands of the leg muscles to support body weight while using the device. In the left VL, there was increased activation in response to the applied resistive torque in late swing while walking in the Resist (red) and Interleaved (blue) modes. This increased activation carried over through initial stance phase, however, in the Interleaved mode VL activity was reduced back to similar levels as the Assist during midstance phase, likely due to the same improvement in posture, while VL activity remained similar to or greater than baseline throughout stance. Importantly, no increases in knee flexor (ST) muscle activity were observed in any mode.

The results from these pilot data, as well as a detailed analysis of the acute biomechanical and neuromuscular effects of NIH-Agilik exoskeleton operational modes [36], indicate the potential success of implementing this device in a longitudinal intervention protocol for children with neuromuscular disorders. We hypothesize that the observed acute changes in knee extensor muscle activation paired with the improvement in knee extension while walking in the device could allow for a rehabilitative effect when used in the long-term. The prior, lab-based study also included a participant with knee extension deficiency from spina bifida who responded well to the applied assistance and resistance torques during overground walking.

## Discussion

This randomized, cross-over study was designed to evaluate the safety, feasibility, and effectiveness of a gait training intervention delivered outside the clinical environment using a wearable robotic exoskeleton. The cross-over design was chosen to enable each participant to receive the experimental intervention, thereby maximizing the incentive for participation while also allowing each to serve as their own control. Additionally, while there is an emerging body of evidence suggesting that exoskeleton-mediated gait training can be effective, at least in the short term, at improving gait biomechanics and self-selected walking speed [14, 15, 31], these initial studies have all been performed in the clinical setting. This study will address the current gap in the scientific literature regarding the use of these mobile technologies outside that environment, despite their unique design for this precise application. Indeed, wearable exoskeletons are a powerful potential tool for delivering the intensive therapy dosages that are required for successful re-training of movements such as stepping and walking [37, 38], and this study is intended to provide a starting point for future deployment of these technologies.

The ICF children and youth version (ICF-CY) provides a framework to classify an individual’s functioning and disability across multiple domains [39, 40]. The ultimate goal of any rehabilitation intervention, especially in children, is to increase an individual’s activity and participation and therefore the ICF-CY framework is also useful when selecting outcome measures to assess the effects of an intervention [39]. Given the goals of this phase I/II study are to establish safety and initial efficacy of community-based, exoskeleton-mediated gait training, our outcome measures focus primarily on ICF domains of body structure, function, and activity. We will evaluate within and between participant differences in gait biomechanics, including kinematics, kinetics, and electromyography, after the 12-week exoskeleton intervention in comparison with the 12-week standard therapy time period to determine efficacy. To assess gait function, we will utilize gait speed, timed up and go, and the 6-minute walk test as outcome measures, while GMFM-66 and PEDI-CAT will be utilized as measures of activity. Finally, the QUEST 2.0 will assess participant and family satisfaction with the exoskeleton during the home-based intervention [51]. This instrument asks participants to rank their satisfaction on a five-point scale regarding the device dimensions, size and weight as well as ease of use, comfort and durability, while also providing space for comments regarding user experience with each. These metrics will be utilized to identify key areas of the mechanical design and usability that require improvement in future versions. Collectively, we anticipate the outcomes from this study will provide meaningful insights for user-based design of exoskeleton interventions delivered outside the clinical study and provide evidence to support larger future studies to evaluate robotic exoskeleton-based gait training effectiveness at improving function and reducing disability in children.

There are several potential limitations to the current study. First, the study intervention takes place outside the clinical environment. While this approach enables the participants to tap the potential for wearable robotic exoskeletons in providing high dosage of intensive gait therapy, it also requires the participant (and their parent/caretaker) to administer the intervention. We previously completed an initial study comprised of 10 visits using the NIH-Agilik exoskeleton in the clinical setting including treadmill and overground walking and observed increased participant comfort and familiarity with the exoskeleton across the visits [36]. Further, our lab previously performed a home-based clinical trial of elliptical training in this same target population and age range that required training 5 days/week for 12 weeks [52]. We therefore believe the proposed home-based exoskeleton intervention is feasible. Yet, it is possible that compliance in completing the prescribed dosage (1 hr/day, 5 days/week) will be variable. To mitigate this, we will track usage during weekly check-ins and provide positive reinforcement in completing the required training. Post-hoc, our analysis will include adherence to the prescribed dosage as a correlate if necessary to account for variable usage. Second, participant growth during the study could be pose multiple challenges, including to the fit of the exoskeleton device as well as changes in gait biomechanics. To mitigate the former, we will include height adjustment in the custom exoskeleton that will enable lengthening the ankle-to-knee distance by up to 3 inches. Our randomized design also includes a 12-week standard therapy period, which will be utilized to control for growth and development between subjects, but it is possible that growth-linked changes in biomechanics will still be present between the intervention and standard therapy periods which will be controlled for as a covariate in the analysis if necessary. Further, our study design does not include a washout period between the exoskeleton and standard therapy interventions. In theory, a washout period allows participants to return to baseline after treatment, but our target population are children who experience natural growth and development and regularly receive physical therapy to maintain and improve daily functioning, which we chose not to ask participants to stop as would be necessary to ensure washout. As a result, carryover effects are possible and we will limit our comparison between the two interventions to the first intervention period for each group. Finally, there is the potential for technical challenges because intervention delivery requires successful operation of the robotic exoskeleton. While we are confident that the exoskeleton and its associated electronics, actuators and sensors are sufficiently robust for use outside the clinical environment, it is possible that unanticipated technical issues may arise that require interrupting the intervention period to perform repairs or adjust the device and control settings, which may affect protocol adherence.

## Supporting information

S1 Checklist SPIRIT-outcomes 2022 checklist (for combined completion of SPIRIT 2013 and SPIRITOutcomes 2022 items)^a^. (PDF)

S1 Table Description of visit procedures by group. (DOCX)

S1 File (PDF)
