# Supplementary material for: A randomized cross-over study protocol to evaluate long-term gait training with a pediatric robotic exoskeleton outside the clinical setting in children with movement disorders
Source: PLoS One. 2024 Jul 8;19(7):e0304087. doi: 10.1371/journal.pone.0304087 (PMC11230531; doi:10.1371/journal.pone.0304087)
Supplement: S1 Table — (DOCX) [file pone.0304087.s002.docx]

**Supplemental Table 1**. Description of Visit Procedures by Group

| **Visit #** | **Group A Visit Name & Procedures** | **Group B Visit Name & Procedures** |
| --- | --- | --- |
| 0 | ***Initial Evaluation, History and Physical***: provision of separate screening consent/assent, review of relevant records, assessments to determine eligibility (knee and ankle joint range of motion, test hamstring contracture, measure foot-thigh-angle, walking test), if eligible enrollment in subject pool and randomization into Group A or Group B will occur | |
| 1 | ***Baseline Assessment:*** vital signs, physical knee joint assessment (Modified Ashworth and Tardieu scales), validated clinical scales of function (PEDI-CAT, GMFM-66, GMFCS, 6-minute walk test, timed up and go), strength testing (Biodex isometric testing), motion capture gait assessment with EMG sensors for walking task with any assistive devices used in daily life  ***Orthosis Digital Casting:*** for creation of lower limb orthotic shells  **Device fabrication time (~4-6 weeks) | ***Baseline Assessment:*** vital signs, physical knee joint assessment (Modified Ashworth and Tardieu scales), validated clinical scales of function (PEDI-CAT, GMFM-66, GMFCS, 6-minute walk test, timed up and go), strength testing (Biodex isometric testing), motion capture gait assessment with EMG sensors for walking task with any assistive devices used in daily life  **Start Standard Therapy Block (12-weeks) |
| 2 | ***Exoskeleton Set-Up and Calibration***: vital signs, examine custom orthotic braces for proper fit, tune and calibrate control settings of NIH-Agilik exoskeleton during walking practice, optional use of FES | ***Orthosis Digital Casting:*** for creation of lower limb orthotic shells *this visit should occur approximately 6 weeks before completion of standard therapy block  **Device fabrication time (~4-6 weeks) |
| 3 | ***Accommodation/In-Lab Training*** [up to 10 visits over 5-weeks]: each session will include vital signs, orthosis examination, exoskeleton walking practice with observation and instruction of exoskeleton operation, optional use of FES | ***Standard Outcome Assessment:*** [to occur at completion of 12-week Standard Therapy Block], vital signs, physical knee joint evaluation, validated clinical scales of function, strength testing, motion capture and EMG for walking tasks without exoskeleton (same procedures as visit 1) |
| 4 |  | ***Exoskeleton Set-Up and Calibration***: vital signs, examine custom orthotic braces for proper fit, tune and calibrate control settings of NIh-Agilik exoskeleton during walking practice, optional use of FES |
| 5 |  | ***Accommodation/In-Lab Training*** [up to 10 visits over 5-weeks]: each session will include vital signs, orthosis examination, exoskeleton walking practice with observation and instruction of exoskeleton operation, optional use of FES |
| 6 |  |  |
| 7 |  |  |
| 8 |  |  |
| 9 |  |  |
| 10 |  |  |
| 11 |  |  |
| 12 |  |  |
| 13 | ***Initial Outcome Assessment:*** vital signs, physical knee joint evaluation, validated clinical scales of function, strength testing, motion capture and EMG for walking tasks with each exoskeleton mode and walking without exoskeleton (same procedures as visit 1) *additional evaluation of ability to participate in community use portion done at this visit  **Start Community Use Block (12 weeks) for Group A2 |  |
| 14 | ***Intermediate Outcome Assessment:*** [to occur approx. 6 weeks/halfway through community use block], vital signs, motion capture and EMG for walking tasks with each exoskeleton mode and walking without exoskeleton  *optional physical knee joint evaluation, validated clinical scales of function, and strength testing |  |
| 15 | ***Final Outcome Assessment:*** [to occur at completion of 12-week Community Use block], vital signs, physical knee joint evaluation, validated clinical scales of function, strength testing, motion capture and EMG for walking tasks with each exoskeleton mode and walking without exoskeleton (same procedures as visit 1 and visit 13)  *Return exoskeleton at this visit  **Complete Patient Experience Assessment (QUEST 2.0) at this visit  ***Start Standard Therapy Block (12 weeks) | ***Initial Outcome Assessment:*** vital signs, physical knee joint evaluation, validated clinical scales of function, strength testing, motion capture and EMG for walking tasks with each exoskeleton mode and walking without exoskeleton (same procedures as visit 1) *additional evaluation of ability to participate in community use portion done at this visit  **Start Community Use Block (12 weeks) for Group B2 |
| 16 | ***Follow-up Outcome Assessment:*** [to occur 6 weeks after completion of Community Use block], vital signs, physical knee joint evaluation, validated clinical scales of function, strength testing, motion capture and EMG for walking tasks only without exoskeleton (same procedures as visit 1, 13 and 15 except without exoskeleton device) | ***Intermediate Outcome Assessment:*** [to occur approx. 6 weeks/halfway through community use block], vital signs, motion capture and EMG for walking tasks with each exoskeleton mode and walking without exoskeleton  *optional physical knee joint evaluation, validated clinical scales of function, and strength testing |
| 17 | ***Standard Outcome Assessment:*** [to occur at completion of 12-week Standard Therapy Block], vital signs, physical knee joint evaluation, validated clinical scales of function, strength testing, motion capture and EMG for walking tasks only without exoskeleton (same procedures as visit 16) | ***Final Outcome Assessment:*** [to occur at completion of 12-week Community Use block], vital signs, physical knee joint evaluation, validated clinical scales of function, strength testing, motion capture and EMG for walking tasks with each controller exoskeleton mode and walking without exoskeleton (same procedures as visit 1 and visit 15)  *Return exoskeleton at this visit  **Complete Patient Experience Assessment (QUEST 2.0) at this visit  ***Start Standard Therapy Block (12 weeks) |
| 18 | N/A | ***Follow-up Outcome Assessment:*** [to occur 6 weeks after completion of Community Use block], vital signs, physical knee joint evaluation, validated clinical scales of function, strength testing, motion capture and EMG for walking tasks only without exoskeleton (same procedures as visit 1, 15 and 17 except without exoskeleton device) |
