# Supplementary material for: A randomized cross-over study protocol to evaluate long-term gait training with a pediatric robotic exoskeleton outside the clinical setting in children with movement disorders
Source: PLoS One. 2024 Jul 8;19(7):e0304087. doi: 10.1371/journal.pone.0304087 (PMC11230531; doi:10.1371/journal.pone.0304087)
Supplement: S1 File — (PDF) [file pone.0304087.s003.pdf]

Abbreviated Title: Pediatric Exoskeleton for Gait Training

NIH IRB #:

Version Date: January 27, 2023

Title: A Phase I/II Trial evaluating long-term use of a Pediatric Robotic Exoskeleton (P.REX/Agilik) to improve gait in children with movement disorders

Principal Investigator: Thomas C. Bulea, Ph.D. Tenure Track Investigator

NIH Principal Investigator: Neurorehabilitation & Biomechanics Research Section
Rehabilitation Medicine Department
National Institutes of Health
Building 21, Room 136
10 Wilson Dr.
Bethesda, MD 20892

TABLE OF CONTENTS

TABLE OF CONTENTS 2
1 PROTOCOL SUMMARY 8
1.1 Synopsis 8
1.2 Schema 9
1.3 Schedule of Activities (SOA) 11
2 INTRODUCTION 13
2.1 Study Rationale 13
2.2 Background 16
2.3 Risk/Benefit Assessment 17
2.3.1 Known Potential Risks 17
2.3.2 Known Potential Benefits 21
2.3.3 Assessment of Potential Risks and Benefits 22
3 OBJECTIVES AND ENDPOINTS 23
4 STUDY DESIGN 27
4.1 Overall Design 27
4.2 Scientific Rationale for Study Design 34
4.3 Justification for Dosage 34
5 STUDY POPULATION 34
5.1 Inclusion Criteria 34
5.2 Exclusion Criteria 35
5.3 Inclusion of Vulnerable Participants 35
5.3.1 Participation of Children 35
5.3.2 Participation of Children of NIH Staff 36
5.4 Inclusion of Pregnant Women, fetuses or neonates 36
5.5 Lifestyle Considerations 36
5.6 Screen Failures 36
5.7 Strategies for Recruitment and Retention 36
5.7.1 Costs 38
5.7.2 Compensation 38
6 STUDY INTERVENTION 39
6.1 Study Interventions(s) Administration 39

6.1.1 Study Intervention Description 39
6.1.2 Dosing and Administration 41
6.1.3 Dose Escalation 42
6.1.4 Dose Limiting Toxicity 42
6.1.5 Dose Modifications 42
6.1.6 Device Administration 43
6.2 Preparation/Handling/Storage/Accountability 43
6.2.1 Acquisition and Accountability 43
6.2.2 Formulation, Appearance, Packaging, and Labeling 43
6.2.3 Product Storage and Stability 43
6.2.4 Preparation 44
6.3 Measures to Minimize Bias: Randomization and Blinding 44
6.4 Study Intervention Compliance 44
6.5 Concomitant Therapy 44
7 STUDY INTERVENTION DISCONTINUATION AND PARTICIPANT DISCONTINUATION/WITHDRAWAL 45
7.1 Discontinuation of Study Intervention 45
7.2 Participant Discontinuation/Withdrawal from the Study 46
7.3 Lost to Follow-up 46
8 STUDY ASSESSMENTS AND PROCEDURES 47
8.1 Screening Procedures 47
8.1.1 Screening activities performed prior to obtaining informed consent 47
8.1.2 Screening activities performed after a consent for screening has been signed 47
8.2 Study Evaluations & Procedures 47
8.2.1 Biospecimen Evaluations 53
8.2.2 Correlative Studies for Research/Pharmacokinetic Studies 53
8.2.3 Samples for Genetic/Genomic Analysis 53
8.2.3.1 Description of the scope of genetic/genomic analysis 53
8.2.3.2 Description of how privacy and confidentiality of medical information will be maximized 53
8.2.3.3 Management of Results 53
8.2.3.4 Genetic counseling 53
8.3 Safety and Other Assessments 53

8.4 Adverse Events and Serious Adverse Events 54
8.4.1 Definition of Adverse Event 54
8.4.2 Definition of Serious Adverse Events (SAE) 54
8.4.3 Classification of an Adverse Event 54
8.4.3.1 Severity of Event 54
8.4.3.2 Relationship to Study Intervention 55
8.4.3.3 Expectedness 55
8.4.4 Time Period and Frequency for Event Assessment and Follow-Up 55
8.4.5 Adverse Event Reporting 56
8.4.6 Serious Adverse Event Reporting 56
8.4.7 NIH Intramural IRB Reporting of IND Safety Reports 56
8.4.8 Events of Special Interest 56
8.4.9 Reporting of Pregnancy 56
8.5 Unanticipated Problems 57
8.5.1 Definition of Unanticipated Problems (UP) 57
8.5.2 Unanticipated Problem Reporting 57
9 STATISTICAL CONSIDERATIONS 57
9.1 Statistical Hypothesis 57
9.2 Sample Size Determination 58
9.3 Populations for Analyses 59
9.4 Statistical Analyses 59
9.4.1 General Approach 59
9.4.2 Analysis of the Primary Endpoint 59
9.4.3 Analysis of the Secondary Endpoints 60
9.4.4 Safety Analyses 62
9.4.5 Baseline Descriptive Statistics 62
9.4.6 Planned Interim Analyses 62
9.4.7 Sub-Group Analyses 63
9.4.8 Tabulation of individual Participant Data 63
9.4.9 Tertiary/Exploratory Analyses 63
10 REGULATORY AND OPERATIONAL CONSIDERATIONS 64
10.1 Informed Consent Process 64

10.1.1 Consent/Assent Procedures and Documentation 64
10.1.2 Consent for minors when they reach the age of majority 65
10.1.3 Considerations for Consent of NIH staff, or family members of study team members 66
10.1.4 Consent of Subjects who are, or become, decisional impaired 66
10.2 Study Discontinuation and Closure 66
10.3 Confidentiality and Privacy: 67
10.4 Future use of Stored Specimens and Data 68
10.5 Safety Oversight 68
10.6 Clinical Monitoring 68
10.7 Quality Assurance and Quality Control 68
10.8 Data Handling and Record Keeping 69
10.8.1 Data Collection and Management Responsibilities 69
10.8.2 Study Records Retention 69
10.9 Protocol Deviations and Non-Compliance 69
10.9.1 NIH Definition of Protocol Deviation 69
10.10 Publication and Data Sharing Policy 70
10.10.1 Human Data Sharing Plan 70
10.11 Collaborative Agreements 71
10.11.1 Cooperative Agreements 71
10.12 Conflict of Interest Policy 71
11 ABBREVIATIONS 71
12 REFERENCES 73
13 APPENDIX 78

<u>Table/Figure List:</u>

Figure 1: Study Schematic Page 10

Figure 2: P.REX/Agilik Device Page 43

Table 1: Group A Schedule of Activities Page 11

Table 2: Group B Schedule of Activities Page 12

Table 3: Objectives and Endpoints Page 23

Table 4: Robotic Exoskeleton Possible Conditions Page 29

Table 5: Outcome Measures Page 30

STATEMENT OF COMPLIANCE

The trial will be carried out in accordance with International Conference on Harmonisation Good Clinical Practice (ICH GCP) and the following:

- United States (US) Code of Federal Regulations (CFR) applicable to clinical studies (45 CFR Part 46, 21 CFR Part 50, 21 CFR Part 56, 21 CFR Part 312, and/or 21 CFR Part 812)

National Institutes of Health (NIH)-funded investigators and clinical trial site staff who are responsible for the conduct, management, or oversight of NIH-funded clinical trials have completed Human Subjects Protection and ICH GCP Training.

The protocol, informed consent and assent form(s), recruitment materials, and all participant materials will be submitted to the Institutional Review Board (IRB) for review and approval. Approval of both the protocol and the consent and assent forms must be obtained before any participant is enrolled. Any amendment to the protocol will require review and approval by the IRB before the changes are implemented to the study. In addition, all changes to the consent and assent forms will be IRB-approved; an IRB determination will be made regarding whether a new consent or assent needs to be obtained from participants who provided consent or assent, using a previously approved consent or assent form.

1 PROTOCOL SUMMARY

1.1 Synopsis

**Title:**	A Phase I/II Trial evaluating long-term use of a Pediatric Robotic Exoskeleton (P.REX/Agilik) to improve gait in children with movement disorders.
**Study Description:**	This randomized crossover trial will determine whether 12 weeks of overground gait training with a robotic exoskeleton outside of the clinical setting has a beneficial effect on walking ability, muscle activity, and overall gross motor function. Participants will be randomized into two groups, one that receives the exoskeleton therapy first before crossing over to continue standard therapy and one that continues standard therapy before completing the exoskeleton intervention. An in-lab training and accommodation period will be completed prior to the exoskeleton being sent home for use outside the clinical setting. We will monitor exoskeleton use during the intervention period for compliance and safety. Assessments of gait biomechanics, neuromuscular activity and functional mobility will be completed before and after the intervention and at 6 weeks post-intervention. It is hypothesized that the 12-week exoskeleton intervention outside the clinic setting will show greater improvements than the standard therapy.
**Objectives:**	Primary Objective: To evaluate the effectiveness of a longitudinal robotic exoskeleton gait training paradigm in improving crouch gait from CP or knee extension deficiency from SB, iSCI or MD in children, assessed as improvement in knee angle during walking before and after the intervention period.<br>Secondary Objectives: To evaluate changes in muscle strength and gait speed following longitudinal intervention with robotic exoskeleton in children with crouch gait from CP or knee extension deficiency from SB, iSCI or MD. Additionally, to evaluate the primary endpoint of knee joint range of motion at multiple time points to assess for order effect and persistence of any observed effect.<br><br>Exploratory Objectives: To assess the effect of exoskeleton dosage (i.e., time spent using the device) on the primary endpoint. Additionally, to evaluate change in knee extensor and flexor muscle spasticity following longitudinal intervention with a robotic exoskeleton in children with crouch gait from CP or knee extension deficiency from SB, iSCI or MD. Additionally, to asses improvement in gross motor function following the same intervention. Finally, to evaluate the safety and feasibility of a community-based protocol for rehabilitation using a pediatric robotic exoskeleton.
**Endpoints:**	Primary Endpoint: Knee extension as assessed by peak knee angle during midstance phase of walking.<br><br>Secondary Endpoints: Change in knee extensor muscle activation and strength after exoskeleton intervention; Change in average gait speed after exoskeleton intervention; Persistence of the primary endpoint

	(change in knee angle) at 6 weeks post intervention; Effect of order of standard therapy and exoskeleton intervention.<br><br>Exploratory Endpoints: Effect of exoskeleton intervention dosage on change in peak knee angle during walking; Change in knee extensor and flexor spasticity after exoskeleton intervention; Improvement in gross motor function after exoskeleton intervention; Feasibility and safety of exoskeleton use outside clinical setting assessed by participant compliance and occurrence of adverse events, respectively.
**Study Population:**	Total requested accrual (44):<br>(22) Patients with cerebral palsy<br>(22) Patients with other neuromuscular disorders (muscular dystrophy, spina bifida, or incomplete spinal cord injury)
**Phase:**	1, 2
**Description of Sites/Facilities Enrolling Participants:**	A single site outpatient study with participants enrolled by the Neurorehabilitation & Biomechanics Research Section, Rehabilitation Medicine Department, NIH Clinical Center.
**Description of Study Intervention:**	Participants will complete 12 weeks of the robotic exoskeleton (P.REX/Agilik) intervention, which includes walking for 1 hour/day, 5 days/week outside the clinical setting. This intervention will be applied following a 10-visit in-lab accommodation period to the exoskeleton. P.REX/Agilik includes a single actuated degree of freedom on each limb at the knee (flexion/extension) and a passive mechanism at the ankle. It can optionally include up to 16 channels of bilateral surface functional electrical stimulation (FES). P.REX/Agilik uses onboard sensors and an embedded controller to track limb motion and apply assistive or resistive torques (and/or FES) during walking. The control intervention is the continuation of the participant's existing standard physical therapy regimen. Participants will be randomized to either complete the exoskeleton intervention or continue their standard therapy for 12 weeks first, followed by a crossover to the other intervention.
**Study Duration:**	72 months
**Participant Duration:**	Up to 38 weeks

1.2 Schema

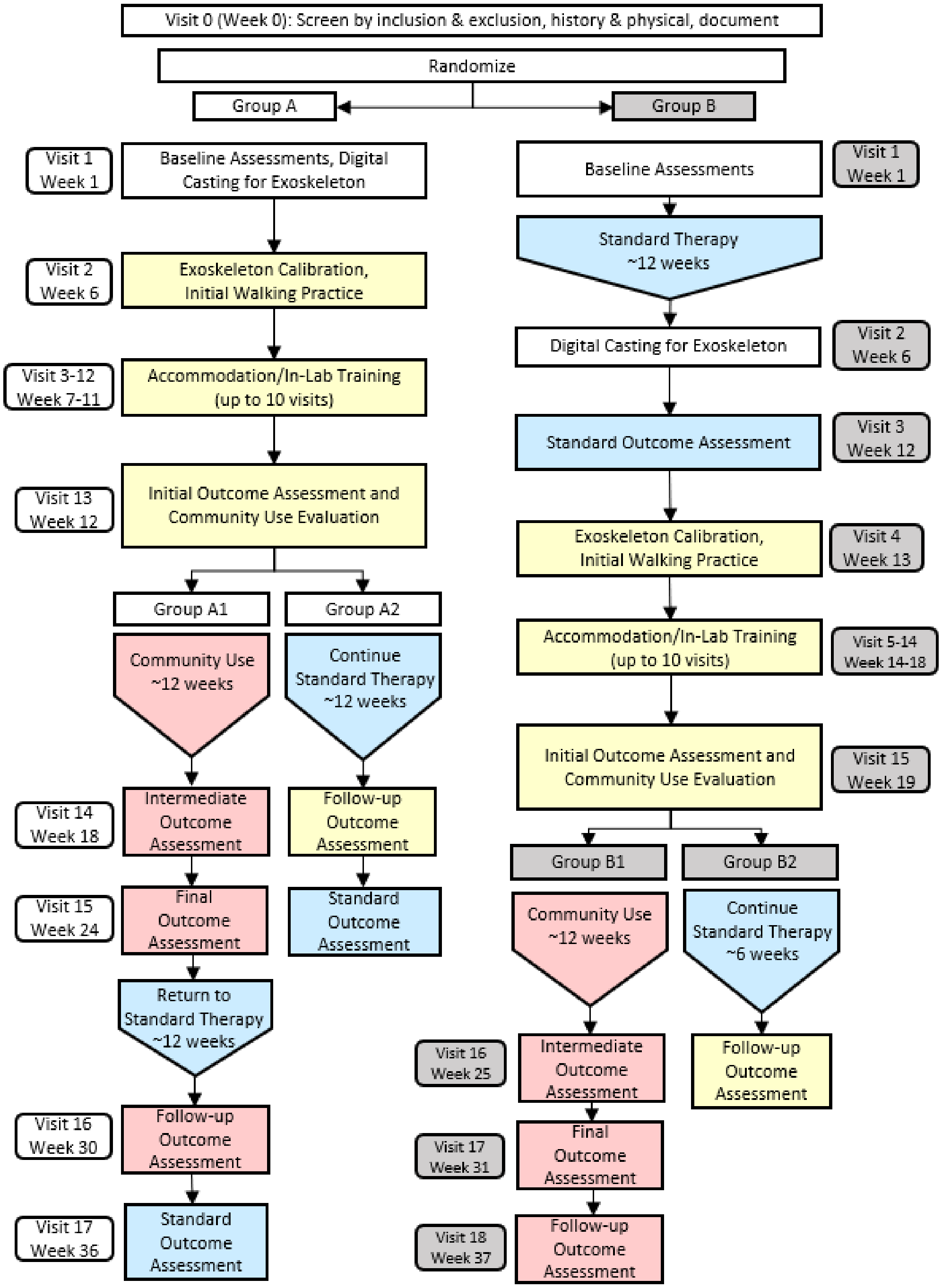

Figure 1. Study Schematic

Yellow indicates 12-week block for exoskeleton set-up and accommodation/training in lab, Blue indicates 12-week block for standard therapy in community, Pink indicates 12-week block for community exoskeleton use finishing with a 6-week follow-up. *Assignment of week number for each visit is subject to change by participant availability. **Two subject pools (CP pool and Neuromotor Disorder pool) will independently complete the above study design, including separate randomization following admission to the protocol, and be analyzed independent of one another.

1.3 Schedule of Activities (SOA)

Table 1. Group A Schedule of Activities

**Procedures**	Screening Visit 0, Week 0	Baseline Visit 1, Week 1	Exoskeleton Set up Visit 2, Week 6	Accommodation/ In-Lab Training Study Visit 3-12 Week 7-11	Initial Outcome Assessment Study Visit 13 Week 12	Community Exoskeleton Use (Group A1) or continue standard therapy (Group A2) Week 13-24	Intermediate outcome assessment Study Visit 14 Week 18	Final Outcome assessment Study Visit 15 Week 24	Return to standard therapy Week 24-36	Follow-up outcome assessment Study Visit 16 Week 30	Standard outcome assessment Study Visit 17 Week 36
Demographics/Medical History	X					-			-		
Physical exam, height/weight	X					-			-		
Pregnancy Test[1]	X					-			-		
Concomitant medication review	X					-			-		
Evaluation of eligibility	X					-			-		
Vital signs[2]	X	X	X	X	X	-	X	X	-	X	X
Informed consent/assent	X					-			-		
Randomization	X					-			-		
Baseline Assessments[3]		X				-			-		
Digital Casting		X				-			-		
Exoskeleton Calibration			X						-		
Overground walking with exoskeleton			X	X	X	(X)	X	X	-	X	X
Outcome Assessment[4]					X	-	(X)	X	-	X	X
EMG		X			X	-	X	X	-	X	X
Motion Capture marker placement		X			X	-	X	X	-	X	X
Photo/Video Recording[5] (optional)		(X)		(X)	(X)		(X)	(X)		(X)	(X)
FES (optional)		(X)	(X)	(X)	(X)	-	(X)	(X)	-	(X)	(X)
Adverse event review	X	X	X	X	X	(X)	X	X	(X)	X	X

[1]Pregnancy test: only required for patients who are able to become pregnant; after screening, individuals will be asked to self-report and re-test as necessary
[2]Vital Signs: blood pressure, pulse oximeter, heart rate, breaths/minute, temperature
[3]Baseline Assessments: [1] Gait analysis (Kinematics – peak knee extension, knee extension at initial contact, step length, gait speed; Kinetics – knee moment; EMG – peak and mean activation of knee extensor and knee flexor muscles), [2] validated clinical scales of function (GMFCS, PEDI-CAT, GMFM-66, 6-minute walk test, timed up and go, modified Ashworth and Tardieu spasticity scales, isometric strength testing on Biodex dynamometer)
[4]Outcome Assessments: the same assessments as baseline except GMFM-66 is only assessed at the baseline, initial and final assessment, at the intermediate assessment - all validated clinical scales of function are optional and at the discretion of the study clinicians
[5]Photo/Video recording separate from the digital cameras involved in the motion capture software will be optional and will require a separate authorization form

Table 2. Group B Schedule of Activities

**Procedures**	Screening<br>Visit 0, Week 0	Baseline<br>Visit 1, Week 1	Continue standard therapy<br>Week 1-12	Digital Casting<br>Visit 2, Week 6	Standard Outcome Assessment<br>Visit 3, Week 12	Exoskeleton Set up<br>Visit 4, Week 13	Accommodation/<br>In-Lab Training<br>Visits 5-14<br>Weeks 14-18	Initial Outcome Assessment and Evaluation<br>Visit 15, Week 19	Community Exoskeleton Use (Group B1) or Continue Standard Therapy (Group B2)<br>Week 19-31	Intermediate Outcome Assessment<br>Visit 16, Week 25	Final Outcome Assessment<br>Visit 17, Week 31	Follow-up Outcome Assessment<br>Visit 18, Week 37
Demographics/Medical History	X		-						-			
Physical exam, height/weight	X		-						-			
Pregnancy Test[1]	X		-						-			
Concomitant medication review	X		-						-			
Evaluation of eligibility	X		-						-			
Vital signs[2]	X	X	-	X	X	X	X	X	-	X	X	X
Informed consent/assent	X		-						-			
Randomization	X		-						-			
Baseline Assessments[3]		X	-						-			
Digital Casting			-	X					-			
Exoskeleton Calibration						X						
Overground walking with exoskeleton			-			X	X	X	(X)	X	X	X
Outcome assessment[4]			-		X			X	-	(X)	X	X
EMG		X	-		X			X	-	X	X	X
Motion Capture marker placement		X	-		X			X	-	X	X	X
Photo/Video Recording[5] (optional)		(X)			(X)		(X)	(X)		(X)	(X)	(X)
FES (optional)		(X)			(X)	(X)	(X)	(X)	-	(X)	(X)	(X)
Adverse event review	X	X	(X)	X	X	X	X	X	(X)	X	X	X

[1]Pregnancy test: only required for patients menstruating; at subsequent visits individual will be asked to self-report an interruption deeming a re-test necessary
[2]Vital Signs: blood pressure, pulse oximeter, heart rate, breaths/minute, temperature
[3]Baseline Assessments: [1] Gait analysis (Kinematics – peak knee extension, knee extension at initial contact, step length, gait speed; Kinetics – knee moment; EMG – peak and mean activation of knee extensor and knee flexor muscles), [2] validated clinical scales of function (GMFCS, PEDI-CAT, GMFM-66, 6-minute walk test, timed up and go, modified Ashworth and Tardieu spasticity scales, isometric strength testing on Biodex dynamometer)
[4]Outcome Assessments: the same assessments as baseline except GMFM-66 is only assessed at the baseline, initial and final assessment, at the intermediate assessment - all validated clinical scales of function are optional and at the discretion of the study clinicians
[5]Photo/Video recording separate from the digital cameras involved in the motion capture software will be optional and will require a separate authorization form

2 INTRODUCTION

2.1 Study Rationale

Cerebral palsy (CP) is the most common pediatric motor disorder (Molnar, 1991), affecting over 17 million people worldwide (Novak, Hines, Goldsmith, & Barclay, 2012). Among the various subtypes of CP, the most prevalent is spastic bilateral CP, or spastic diplegia, occurring in over 50% of the CP population (Yeargin-Allsopp, et al., 2008). In spastic diplegia, motor deficits manifest in various abnormal movement patterns, particularly in walking. Motor impairments lead to a variety of gait patterns including crouch gait, a walking pattern characterized biomechanically by exaggerated stance phase knee flexion; crouch may be accompanied by increased hip flexion, adduction and internal rotation, and a plantarflexed, neutral, or dorsiflexed ankle (Binder & Eng, 1989; Perry, 1990). The cause of crouch is often multifactorial including; overlengthening of the triceps surae, weakness or deficiency in knee or hip extension and or spasticity or contracture in the knee or hip flexors (Hicks, Schwartz, Arnold, & Delp, 2008). While CP is defined as a non-progressive disorder from a neurological standpoint, a crouch gait walking pattern often progresses during a child's motor developmental period resulting in a decline or worsening in mobility and other functional tasks; in fact, it has been reported that approximately 50% of those with CP who are ambulating as adolescents will cease to do so in early or middle adulthood due to worsening musculoskeletal deformities reinforced by crouch gait (Bottos & Gericke, 2003). Musculoskeletal deformities compound with secondary effects of reduced physical activity, strength and endurance (Bottos & Gericke, 2003).

The standard of care for treating knee extension deficiency in CP and other neuromotor disorders includes three approaches: (1) surgical interventions, (2) chemodenervation procedures including botulinum toxin injections, and (3) physical therapy. When crouch gait is caused primarily by muscle contracture, the standard treatment is orthopaedic surgery to increase muscle-tendon length (Olney, MacPhail, Hedden, & Boyce, 1990). Surgical outcomes, however, are often variable (Galey, Lerner, Bulea, Zimbler, & Daminao, 2017), as muscles could become overlengthened or weakened, leading to exacerbation of crouch rather than correction in the long term (Olney, MacPhail, Hedden, & Boyce, 1990; Vuillermin, et al., 2011). Botulinum toxin (Botox) injections to spastic muscles (Thompson, Baker, Cosgrove, Corry, & Graham, 1998), including lower limb flexors have also been utilized to treat crouch gait. Botulinum toxin injections aim to treat crouch by targeting overactivity/spasticity in knee flexion muscles. Botulinum toxin injections may demonstrate some limited improvements in the short term, but long-term deficits frequently persist, regardless of treatment (Dreher, et al., 2012; Rethlefsen, Yasmeh, Wren, & Kay, 2013). Ultimately, Botulinum toxin injection treatments should be used in tandem with physical therapy. Physical therapy for locomotor training, including standard clinical practice and muscle strength training (Damiano, Kelly, Vaugh, Westcott, & Lowes, 1995), as well as gait physical therapy guided by body weight support and robotic-assisted step training on a treadmill (Dobkin & Duncan, 2012) underlies the standard of care for pediatric crouch gait.

In comparison to strength and resistance-based training, interventions focused on gait training are found to be most effective at improving gait speed (Moreau, et al., 2016; Novak, et al., 2020). There is evidence to suggest body weight support (BWS) treadmill training (TT) is an effective rehabilitation intervention to improve gait speed in children with CP, but it is not clear if this

type of intervention would prove more or less effective than TT without BWS or overground training without BWS (Booth, et al., 2018). A study testing overground training with BWS in children with CP found a larger effect on locomotor ability than overground training without BWS, likely due to the stimulation of natural walking, although the study was unable to control for the benefit of unloading the lower limbs on increased gait speed (Emara, El-Gohary, & Al-Johany, 2016).

Ultimately, the limitation of each of the above treatments – or a combination thereof – is a lack of evidence for long-term improvement of crouch gait and its associated clinical consequences. As a result, additional high-quality trials are necessary to assess the impact of the dosage, the intensity and the volume of training necessary to see persistent, long lasting improvements in function. In addition, assessment of devices that can be incorporated into the child's home program, i.e. used outside the clinical setting for overground walking would be ideal as it would provide the capability to test these parameters in a more realistic environment e.g. home, community, school.

Robotic assisted gait trainers' contrast with BWSTT because the robot can be customized for patient specific impairments and can assist in all or part of the individual's gait cycle without requiring assistance from the participant or therapist (Lefmann, Russo, & Hillier, 2017). However, the existing robotic assisted gait trainers present with substantial limitations and shortfalls. First, the use of the robotic-assistance device requires a treadmill to operate. This pairing does not replicate the demands of the lower-extremity during over-ground walking tasks (Dobkin & Duncan, 2012). Secondly, the robotic device and the treadmill belt continue to operate regardless of effort by participant and it may be difficult or impossible to measure the level of the subject's engagement and awareness during the task and to determine whether the device is just passively moving the patient without their participation. (Dobkin & Duncan, 2012). A study by Žarković, et al. (2021), found children with spastic diparesis had decreased volition in motor control and muscle activation of their lower limbs during walking trials when employing a robot-assisted gait training mechanism on a treadmill, further supporting the point that currently available robotic assisted gait trainers are not optimized for motor training in this population.

To address these shortfalls, we designed and tested a novel wearable robotic exoskeleton which executed precisely timed knee extension assistance at specific points in the gait cycle and found the device encouraged volitional muscle activity (Lerner, Damiano, & Bulea, 2016). We applied this device to an observational cohort study under a previous, IRB approved protocol (#13-CC-0210) at the NIH Clinical Center and found it's use to be safe for overground training with success in altering posture, gait biomechanics and muscle activity in a clinical lab setting (Bulea, Lerner, & Damiano, 2018; Lerner, Damiano, & Bulea, 2017a; Lerner, Damiano, & Bulea, 2017b; Lerner, Damiano, & Bulea, 2016; Lerner, Damiano, Park, Gravunder, & Bulea, 2016).

As a result of these initial findings, a second novel wearable robotic exoskeleton (P.REX/Agilik) was developed to reach a wider patient population and to find an eventual application outside of the clinical setting (Chen, et al., 2021). The exoskeleton was developed under a cooperative and research development agreement with Bionic Power, Inc. (CRADA, CC#03240). Briefly, the new system combined the Agilik actuators from Bionic Power with the embedded control system and associated sensors previously developed at NIH. We evaluated P.REX/Agilik with a healthy participant and a subject with bilateral spastic CP (GMFCS III) under the prior protocol (#13-

CC-0210). Prior to its evaluation, we submitted an FDA risk assessment for the P.REX/Agilik and it was found to be a non-significant risk device. Two knee torque profiles were examined and validated as exoskeleton assistance mechanisms in overground walking; constant mode (characterized by constant assistive knee extension and/or flexion during all phases of the gait cycle) and adaptive mode (characterized by assistive torque applied proportional to an individual's estimated knee extension moment in stance phase of the gait cycle). For this participant with CP, the adaptive mechanism showed the best improvement in walking mechanics (Chen, et al., 2021). These preliminary findings suggest a potential to personalize control strategies to each individual with CP and to apply the training over a longer time period (Chen, et al., 2021).

Along with refining the hardware, we also expanded our software control to provide not only assistance to knee extension but also resistance to that motion. The objective is to provide on demand resistance to knee extension during walking that the user must overcome to complete each step; in this way, the device functions as a training aid to strengthen knee extensor muscles. We recently assessed the immediate effects of this interleaved assistance and resistance control strategy on the biomechanics and neuromuscular activity of a single participant diagnosed with crouch gait from CP (GMFCS level III) to establish the initial feasibility of this approach (Bulea, Molazadeh, Thurston, & Damiano, 2022). The control system displayed a high level of accuracy in providing appropriately timed torques to assist and resist knee extension, demonstrating the feasibility and validity of this novel interleaved approach for exoskeleton-based gait training during overground walking in children with CP (Bulea, Molazadeh, Thurston, & Damiano, 2022).

Additionally, we amended the previous protocol to expand the target population. In addition to CP, knee extension deficiency related gait deviations is a common problem in other pediatric neuromuscular disorders, including spina bifida (Moen, Gryfakis, Dias, & Lemke, 2005), muscular dystrophy (Doglio, et al., 2011), and incomplete spinal cord injury (Smith, Hassani, Reiners, Vogel, & Harris, 2004). There are inherent differences in the impairments seen in CP and these other pediatric neuromuscular disorders and the underlying the mechanism of gait pathology. Whereas the gait deviations in CP are typically multifactorial (motor control, weakness, spasticity) (Steele, Seth, Hicks, Schwartz, & Delp, 2010), the gait pathology observed in patients with SB, MD or iSCI are typically a result of muscle weakness (Sandler, 2010; Doglio, et al., 2011; Jayaraman, et al., 2006). A child with gait pathology from spina bifida enrolled in and completed our previous protocol (#13-CC-0210). The results demonstrated the initial safety and effectiveness of a wearable robotic exoskeleton providing knee extension assistance and resistance during overground walking in a child with spina bifida.

Clinical and basic neuroscience of motor learning principles suggest that high intensity and high dosage training is necessary to have successful outcomes for functional improvements to gait locomotion (Hornby, et al., 2011). Thus, promising therapies, such as robotic-assisted gait training, need to be investigated at higher dosages. Wearable exoskeletons suitable for overground walking outside of the clinical setting open the window of possibility for increased dosage and or intensity of training for children with neurological disorders. However, the evidence from research studies using wearable robotics for gait training in natural/community/home settings is extremely limited.

The purpose of this study is to evaluate safety, dosing and initial efficacy of a wearable exoskeleton for overground gait training in home or community environments.

2.2 Background

Wearable devices that provide some type of external assistance and/or support to help a person perform a functional task, often referred to as robotic exoskeletons, are increasingly available as training/assistive devices. Preliminary evaluation of a wearable robotic exoskeleton designed at NIH for children with CP showed that short-term use of an extension assist robotic exoskeleton improves knee extension in those with crouch gait (protocol #13-CC-0210) (Bulea, Lerner, & Damiano, 2018; Lerner, Damiano, & Bulea, 2017a; Lerner, Damiano, & Bulea, 2017b; Lerner, Damiano, & Bulea, 2016; Lerner, Damiano, Park, Gravunder, & Bulea, 2016). The engineering design of this device differs from other available robot-assisted gait trainers, which primarily focus on restoring lost function (Farris, et al., 2014), by addressing the unmet need of training a different walking pattern rather than to restore lost walking capability in children with CP. The majority of successful wearable robotics used for gait rehabilitation have been designed as a tool for physical therapy, and thus are implemented only in the clinical setting (Lefmann, Russo, & Hillier, 2017). The NIH wearable robotic exoskeleton (P.REX/Agilik) however, is designed for overground walking so as to be a powerful tool for high dosage training outside the clinical setting.

Rather than guiding the lower limbs, the exoskeleton dynamically changes limb posture by introducing bursts of knee extension assistance during discrete portions of the walking cycle, a perturbation that resulted in maintained or increased knee extensor muscle activity during exoskeleton use in a prior study (Lerner, Damiano, & Bulea, 2017a). In this preliminary investigational use of the device on children with cerebral palsy, participants exhibited postural improvements equivalent to outcomes reported from invasive orthopaedic surgery, and these improvements in crouch increased over the course of our multiweek exploratory trial (Lerner, Damiano, & Bulea, 2017a). These preliminary findings pave the way for the longer-term investigation of wearable exoskeletons for children with neurological disorders. Accordingly, recent implementation of robotic gait training for long-term neurorehabilitation practice demonstrates positive results (Damiano, Stanley, Ohlrich, & Alter, 2017), but comparisons of rehabilitation outcomes from overground exoskeleton gait training with equivalent dose alternatives or with other control systems have not yet been reported. The initial findings of the immediate locomotor benefits of the NIH exoskeleton, along with other studies from our group at NIH that demonstrated immediate effects of unilateral leg-weighting on spatiotemporal and gait kinematics during treadmill walking (Bulea, Stanley, & Damiano, 2017; Damiano, Stanley, Bulea, & Park, 2017), make it clear that the target population can adapt gait in response to a perturbation. However, for efficacy and long-term retention of these adaptations to occur, higher dosage of treatment is necessary. In fact, inadequate dosing is likely a factor for why highly effective therapies for improving gait and gross motor function remain elusive in the medical care of children with CP (Damiano, Stanley, Ohlrich, & Alter, 2017).

There is evidence to support higher intensity dosage in gait training without the use of devices (Bleyenheuft, Arnould, Brandao, Bleyenheuft, & Gordon, 2015). A study aimed to evaluate the efficacy of high-intensity continuous training by subjecting participants with unilateral spastic cerebral palsy to intensive bilateral upper- and lower-limb training of approximately 90 hours over a 10-day period. When compared with the same dosage of conventional therapy over an

extended period of time, there were significant positive results favoring the high-intensity training as shown by the 6-minute walk test for the lower extremity and the Assisting Hand Assessment (AHA) for the upper extremity (Bleyenheuft, Arnould, Brandao, Bleyenheuft, & Gordon, 2015).

Wearable devices are a promising candidate as a highly effective therapy in locomotor training because they can efficiently and safely facilitate high dosage, high intensity training. Few studies investigate the clinical benefits of a high dose of assistive device control or activity-based neurorehabilitation therapies specifically targeting gait locomotion improvements. Similar to strength training in the lower extremities (Merino-Andrés, García de Mateos-López, Damiano, & Sánchez-Sierra, 2022), robotic-assisted gait training may take far more practice before true functional benefits from the robotic and device therapies become evident. Previous work at NIH investigated a lower training dosage of ≤ 20 hours of elliptical vs. cycle training in children with CP with task specific improvements but no transfer of improvements to gait or functional mobility at the group level (Damiano, Stanley, Ohlrich, & Alter, 2017). The results of that 12-week study suggest that the training dose may have been insufficient to produce appreciably clinical change (Damiano, Stanley, Ohlrich, & Alter, 2017).

In a different device-assisted gait study at NIH, 14 children with cerebral palsy used a functional electrical stimulation device for approximately 6 hours/day for three months during walking. Following intervention period, children displayed increases in muscle size suggesting that repetitive and intensive implementation of device-assisted therapy such as functional electrical stimulation may lead to functional gait improvement over time (Damiano, Prosser, Curatalo, & Alter, 2013).

Other groups have investigated higher-dosage training with different devices aimed at improving gait function in this population, such as a home-based virtual cycling trainer (Chen, et al., 2021). In this 12-week study, children with spastic CP engaged in an at-home, virtual reality based interactive cycling therapy for a total of ~24 hours and demonstrated significant improvements to gross motor function, as measured by the Bruininks-Oseretsky Test of Motor Performance (BOTMP), including running speed and agility, bilateral coordination, and knee extensor strength. The evidence supporting functional improvements and motor rehabilitation following long-term, high dosage interventions is one of the keys to unlocking the potential for functional recovery in children with CP and other neuromuscular disabilities (Damiano, 2006). This, coupled with the preliminary evidence suggesting immediate benefits of a robotic exoskeleton, merits the investigation of regular exoskeleton use over a longer period of time outside of the clinical setting.

2.3 Risk/Benefit Assessment

2.3.1 Known Potential Risks

a. *Screening for inclusion:* The procedures included in the screening visit are a medical history and concomitant medication review, a physical examination, a pregnancy test (if applicable), mobility/range of motion exams and a walking assessment. Each of these procedures poses minimal to no risk to the participant. The pregnancy test will only be performed for patients who are able to become pregnant. This test will require a urine sample. If the test were to return positive, the participant would be excluded from participating in the study. The study procedures do not present more risk to than those of

everyday life. But, the physical changes that occur during pregnancy can affect the gait pattern, which would confound the results of the exoskeleton intervention. Therefore, pregnancy is listed as an exclusion criterion. We will ask individuals to self-report interruption of menstruation to determine if re-testing is necessary at subsequent visits. The review of medical history and physical examination will be performed by a licensed RMD physician and will not present any risk to the participant. The mobility and range of motion testing involves physical manipulation of the lower limbs by a trained RMD clinician. The patient will be stationary, prone on the examination table or seated for most of these procedures. The patient will also be asked to walk across the room to provide validation of walking ability. Each of these tasks present no greater risk than activities of daily living and will be closely monitored by a trained RMD clinician and a study team member at all times.

b. *Vital Signs:* Measurements include temperature, heart rate, respiratory rate, pulse oximeter and blood pressure. The procedures to acquire these measurements present minimal to no risk to the participant and will be performed by a licensed RMD clinician.

c. *Orthotic braces*: Each participant will undergo digital casting to create a custom-fit orthotic brace to accompany their use of the exoskeleton. In the event that digital casting is not possible (due to involuntary movements or patient cooperation) standard casting will be performed. The orthosis will be outfitted with a soft silicone or foam cushion for added comfort between the plastic structure and the user's skin. Stockinette or a sock will be placed on the limb prior to donning the device. Even with these precautions, participants may feel discomfort while wearing the orthosis and there is a small risk of pressure by the orthotic braces with the potential to cause skin problems. A physical therapist or physician will evaluate the skin prior to donning the device, continuously monitor the participant and orthotic braces during in-lab training sessions and again inspect the skin at the completion of testing at the NIH for any signs of skin irritation or pressure. The patient will also be monitored with verbal questions about any discomfort during the device use. If the subject experiences anything beyond minimal discomfort (extra inertia and resistance against movement), testing will be stopped, the skin inspected, and the device adjusted as needed until the problem is resolved. If any problem is observed, the physical therapist will report to the medically responsible individual or another RMD physician for further evaluation. Additionally, during the accommodation/in-lab training period we will instruct the participant and their caretaker(s) on how to self-monitor for skin irritation, breakdown or discomfort during device use. If any skin redness persists for 10 minutes after device doffing or significant discomfort is experienced during the community use portion of the protocol, the family/patient/therapist will be instructed to cease use of the device and return to NIH for an inspection and device adjustment.

d. *Walking with P.REX/Agilik*: The protocol includes up to 10 visits at the NIH clinical center to allow for participant accommodation to overground walking with the P.REX/Agilik. During these visits, engineers will work closely with the participant and their care-taker and/or physical therapists to ensure the settings of the exoskeleton are tolerable and safe for use. Use of the P.REX/Agilik presents several risks. First, there is a risk of falling when participants are walking with P.REX/Agilik. The exoskeleton applies assistive and/or resistive torque to the participant's legs which may cause loss of balance. During the accommodation/in-lab training process, a physical therapist will

closely monitor the participant while walking with the P.REX/Agilik. If the participant appears unstable during any part of this process, we may attach a harness (ZeroG®, Aretech LLC) while using the P.REX/Agilik to protect them from falling. Participants will only proceed to community use of the exoskeleton after the 10 in-lab sessions are completed and the use of the exoskeleton is deemed safe by the medically responsible investigator (physician), the treating physical therapist and the principal investigator. During over-ground training at NIH and in the community using the P.REX/Agilik the participant is allowed to use whatever assistive device (gait trainer, walker, canes, crutches) is used during in home and community ambulation.

The P.REX/Agilik control system will guard against possible malfunction by enabling a preset (operator controlled) maximum torque magnitude. The embedded software of the exoskeleton will monitor the torque applied to the limb to ensure the applied torque does not exceed this preset maximum. Similarly, the knee range of motion can be programmed into the software and the controller will automatically stop the robotic actuator from providing torque if/when knee angle is measured to be outside this range. Finally, the exoskeleton is equipped with an emergency switch that is easily reached by the participant or a physical therapist; pressing this switch cuts the system power. It can be pressed at any time if the device is operating abnormally or if the participant experiences discomfort. When participants are operating the device in the community, they will have access to the same emergency switch at all times with the ability to cut the system power as needed. They will be trained on independent usage of this emergency switch during the accommodation/in-lab training period.

The prior NIH protocol #13-CC-0210 using this robotic exoskeleton (P.REX/Agilik) was evaluated by the FDA and was determined to be non-significant risk (see Appendix Document B). Additionally, the NIH-Agilik device is now an approved class I device under FDA regulations (Regulation Number 890.3475; Establishment Registration & Device Listing (fda.gov)). Given this classification and the previous FDA assessments from our earlier protocols as non-significant risk device studies, we believe the NIH-Agilik device is a non-significant risk device for the purposes of this study.

e. *Use of P.REX/Agilik outside of clinical setting*: P.REX/Agilik is a medical device of minimal risk to its users. The use of P.REX/Agilik outside of a clinical setting has not been studied before, so we cannot anticipate all potential risks of such a task, however we intend to mitigate any potential issues by proper education of and adequate accommodation for the participant and their caretaker(s) prior to taking the device home. All participants will complete up to 10 total visits using the device within the clinical setting before they can decide to participate in the use of the device outside the clinical setting. All subjects may also opt out of using the device in the community/home if they do not wish to participate in this component of the protocol. All subjects participating in the device use outside of the clinical setting will be properly instructed on at-home use and care, which will include donning and doffing the exoskeleton, operating the device through the wireless interface, charging, storage, and cleaning. This instruction will occur during the 5-week accommodation/in-lab training period consisting of up to 10 visits. A brochure detailing the education material of the device components and instruction for operation at home will be provided for the participant. This will include donning and doffing the exoskeleton safely and in inspecting the skin and proper fit of the device while wearing, to ensure the device is comfortable and safe while being worn. These

instructions will also be reviewed at each of the 10 visits during the participants accommodation/in-lab training sessions and the treating physical therapist and principal investigator will test the participant and their caregiver at the final session to be sure they are confident with independent use. Another component of the device is the software interface that must be controlled on a tablet or laptop. If the participant does not have access to a suitable device to run this software, we will provide one for use during the trial. The participant and their caregiver will be properly counseled on connecting and powering the software, as well as checking the software to be sure it is properly running while the device is in use to mitigate the potential for device malfunction while being used without the supervision of our study team. The device must be properly stored and charged in preparation of the exoskeleton use. The participant and their caregiver will be counseled in the proper care of the device, which will include storage and charging, to ensure it functions properly and safely during the devices use.

Wearable exoskeleton use includes inherent risk of user error, such as improper charging of the device or mistakes with donning or doffing the device which may pose minimal risk of injury to the participant when performed unsupervised (He et al., 2017). User error by the caregiver is also a potential risk, such as improper monitoring during use and failure to monitor and support the subject if they lose balance (He et al., 2017). All of these potential risks will be mitigated by proper instruction and adherence to protocols presented during the 5-week, 10 session in-lab training period. The participant will not be responsible for changes to the control settings of the device either at NIH or in the community. The in-lab training period will also function to test the various settings on the device to determine the best fit for each participant. This will be deemed the primary set point and will be programmed for the participant prior to them taking the device into the community. If they find while using the device in the community that they need to change any of the primary set point controls, we can alter these at their intermediate outcome assessment visit in our facility, or they can return prior to this assessment if necessary to have them adjusted.

f. *Motion capture markers*: The adhesive tape placed on the skin to attach the marker set may cause mild skin irritation. All motor assessment data collection will be performed in the Neurorehabilitation and Biomechanics Research Section under closely monitored conditions. A physical therapist or physician will be present at all times during data collection to ensure safety and to assist subjects. Refer to *Walking with the P.REX/Agilik* (Section 2.3.1d) to review potential minimal risk associated with walking trials during data collection sessions.
g. *EMG*: There is minimal medical risk associated with surface EMG. The EMG sensors are placed over the lower limbs using self-adherent cohesive bandages. This material adheres to itself but not human skin and is not intended to cause skin irritation. Throughout each visit and when the sensors are removed, the participants skin will be examined to ensure no irritation occurred.
h. *Functional electrical stimulation (FES) device*: This device may cause discomfort if the stimulation current is too strong. As with clinical use, the current intensity will be increased slowly with continuous feedback from the subject on whether they feel any discomfort. If they experience discomfort, the current intensity will be decreased until discomfort is no longer present. The aim is to reach a stimulation threshold that achieves

fused contraction of the target muscle, not to elicit a maximal or near maximal contraction.

i. *Questionnaires:* The participant will be asked to complete two questionnaires: the PEDI-CAT and the QUEST 2.0. The PEDI-CAT is completed on the computer and asks the patient about their daily activities, mobility and social and cognitive ability. The QUEST 2.0 is a paper survey that will ask about the patients experience with the exoskeleton in the community. There is the potential for certain questions to make the patient upset or feel uncomfortable answering. The patient can skip any questions they do not wish to answer, or stop the questionnaire at any time.

j. *Photo/Video Recording (optional):* Photos and videos of the participant wearing and walking in the P.REX/Agilik device can occur at any visit to the NIH. However, the participant can choose to sign an Authorization for Recording, Filming, and/or Photographing of Patients in the Clinical Center (Appendix Document F) to give permission to use the videos or photographs for publications and public presentations involving this study. On signing this authorization form, the participant can denote whether or not they would like any identifying features (i.e. face) obscured when used in public presentations or publications. If the participant chooses not to sign this authorization, no photographs and videos involving that participant will be used in public presentations of this study's work. With all electronic media, there is a very small risk of a data breach and if someone were to gain access to the media files of the participant, we would not be able to control their use. This will be minimized by securely storing the files in the patients record at the NIH. The authorization for photo and video recording will allow photography and filming for research purposes only.

k. *Potential Side Effects:* The following list is not all inclusive of potential side effects, but includes both anticipated/minor and unanticipated/serious side effects of robotic exoskeleton training. These conditions are listed as a result of previous incidences reported in the literature from prior research involving a robotic exoskeleton.

(1) Anticipated/Minor Side Effects	Muscle soreness, fatigue, skin pressure, skin irritation and/or abrasion injuries
(2) Unanticipated/Serious Side Effects	Soft tissue injury, broken bone, shortness of breath / cardiovascular and/or respiratory events

2.3.2 Known Potential Benefits

a. P.REX/Agilik: Our previous research has focused on assessment of the robotic exoskeleton to be used in this study in a clinical lab setting (Bulea, Lerner, & Damiano, 2018; Lerner, Damiano, & Bulea, 2017a; Lerner, Damiano, & Bulea, 2017b; Lerner, Damiano, & Bulea, 2016; Lerner, Damiano, Park, Gravunder, & Bulea, 2016). The results from these previous cohort studies have shown the potential benefits of the robotic exoskeleton as an assistive device. Specifically, use of the robotic exoskeleton has resulted in increased peak knee extension, increased knee range of motion, improved step length and improved muscle activity. This prior research has focused on children with crouch gait from cerebral palsy, although our prior protocol (13-CC-0210) was amended to include individuals with knee extension deficiency from other neurological disorders including spina bifida, incomplete spinal cord injury or muscular dystrophy. Earlier

studies have shown the potential for lower limb gait training devices to be beneficial for these patient populations (Sarajchi, Al-Hares, & Sirlantzis, 2021). One participant with spina bifida completed the prior protocol (10 total visits) and her results with the exoskeleton showed the same benefits from the assistance as those with CP (increased knee extension during walking and improved muscle activity).

Strength training paradigms targeting lower extremity muscles are known to be beneficial for gait function in cerebral palsy as indicated by improved gait speed and gross motor function (Dodd, Taylor, & Damiano, 2002). Robotic exoskeleton interventions, which apply precisely timed assistive and/or resistive torque during the gait cycle in over ground walking, combine strength training with training of selective neuromuscular control to produce short-term beneficial adaptations in lower-limb mechanics for children with crouch gait from cerebral palsy and other neuromotor disorders. Under our existing protocol (#13-CC-0210) we have piloted the use of the robotic exoskeleton to apply resistive torques to knee extension (Bulea, Molazadeh, Thurston, & Damiano, 2022). This previous study showed the feasibility of individuals with cerebral palsy to overcome resistive torques while maintaining functional ambulation. As expected, we observed increased knee extensor muscle activation without an increase in flexor muscle activation under the interleaved paradigm that provided on demand resistance to knee extension during late swing (Bulea, Molazadeh, Thurston, & Damiano, 2022). The findings of this pilot study suggest the potential for improved strength and selective motor control following an appropriate, longitudinal training intervention, which this new protocol aims to address, given that improvements observed in the short-term must be reinforced over time to translate into long-term benefits on motor control and learning (Roemmich & Bastian, 2018), as well as to see potential therapeutic effects on neuromuscular and gait biomechanics following removal of the exoskeleton (Bulea, Molazadeh, Thurston, & Damiano, 2022). Thus, the potential benefits of the robotic exoskeleton following longitudinal use include improved knee extension and step length, increased knee extensor strength and better selective motor control. Taken together, these may result in improved gait function.

b. *Surface functional electrical stimulation (FES):* Under our existing protocol a neuromuscular electrical stimulation (NMES) system was investigated by a single young participant with bilateral spastic cerebral palsy and was found to immediately improve mean peak knee extension when NMES was applied to the *vastus lateralis* and *rectus femoris* during stance phase (Shideler, Bulea, Chen, Stanley, & Damiano, 2020). An earlier study on children with spastic cerebral palsy also demonstrated the application of electrical stimulation to the quadriceps muscle resulted in a statistically significant increase in stance phase knee extension (Postans & Granat, 2005). We anticipate long-term use of electrical stimulation to the quadriceps muscle during ambulation could enhance the knee extension improvement over time as a result of increased muscle strength and improved spasticity and unassisted gait kinematics (Shideler, Bulea, Chen, Stanley, & Damiano, 2020).

2.3.3 Assessment of Potential Risks and Benefits

The potential benefits outweigh the minimal risks associated with participation in this study. The target population consists of children who are ambulatory, either

independently or with an assistive device such as an orthotic or mobility aids such as canes, crutches or walkers. The children being recruited will also likely have experience with gait training assistive devices, such as body-weight support treadmill gait trainers, from their previous therapy for crouch gait meaning they are predisposed to the demands on the lower-limb while wearing an assistive device and will have experience ambulating with hardware on their lower-limbs.

The Agilik device has been registered as a class I device by the FDA. Given this classification and approval, combined with the two previous FDA assessments of the devices use as non-significant risk in our earlier protocols, we believe the NIH-Agilik exoskeleton does not meet the definition of a significant risk device (as defined in 812.3(m)), and therefore is a non-significant risk device.

The minimal risks described in section 2.3.1 include the potential to fall while ambulating, skin aberrations or irritability and user error. We intend to mitigate the risk of user error by properly counseling participants and their caregiver(s) of device assembly and usage during a 6-week supervised accommodation/in-lab training period in our facility. This in-lab training period will also serve to mitigate the risk of falling during walking trials and with usage outside of the facility. Each device will be custom-fit using digital casting to limit the potential for improper alignment that could result in injury or skin irritation. For added comfort, the patient will put on a long AFO sock or stockinette before donning the device and soft cushioning will be placed between the plastic frame of the orthotic and the skin, if needed.

As described in section 2.3.2, the potential benefits of participation in this study all result in increased function during walking for the participants both during the study and potentially following its completion. There also exists the potential benefit to the body of knowledge that will be gained on wearable exoskeleton device training from this study. There is a gap in the literature surrounding the effectiveness of longitudinal wearable exoskeleton training outside of the clinical setting, especially when compared with the current standard therapy for gait training. We will gain scientific knowledge on compliance with protocols outside of the clinical setting, as well as initial information regarding tolerance to and dosing of regular exoskeleton gait training, which will be fundamental to the design of future long-term training paradigms.

3 OBJECTIVES AND ENDPOINTS

Table 3. Objectives and Endpoints

OBJECTIVES	ENDPOINTS	JUSTIFICATION FOR ENDPOINTS
Primary		
(1) To evaluate the effectiveness of a longitudinal exoskeleton training program in the community, in comparison to standard therapy, to improve crouch gait in children with CP	(1) The primary endpoint is knee extension during walking without the exoskeleton. This will be assessed as change in peak knee angle at midstance between two time points: initial outcome assessment and final outcome assessment which occur immediately before and after the 12-week community use block.	(1) Our previous research has demonstrated the effectiveness of our robotic exoskeleton for gait enhancement in children with CP utilizing peak knee angle as the primary outcome measure. This pilot data indicated, on average, a 2-3°

or knee extension deficiency in children with SB, iSCI or MD.	The objective will be evaluated at the final outcome assessment, which corresponds to visit 15 for Group A and visit 17 for Group B.<br><br>An optimal result would be an observed reduction in the knee flexion angle during walking or standing, indicating improved knee extension deficiency, as a result of robotic exoskeleton training. An improvement in peak knee extension of more than 10° will be considered the threshold for clinical significance based on a study that characterized children with crouch gait as mild, moderate or severe by increments of 10° in knee angle *(Hicks, Schwartz, Arnold, & Delp, 2008)*.	improvement in knee extension following a 3-8-week observational period. We expect to replicate this improvement, if not increase the magnitude of this improvement, with the higher dosage of training implicated in this protocol.<br><br>We also expanded the target population to include children with other neuromuscular disorders, including SB, MD or iSCI, that result in knee extension deficiency. Our pilot study with a child with SB presented initial effectiveness and safety of use of the robotic exoskeleton in overground walking.
Secondary		
(1) To evaluate changes in peak and overall volitional knee extensor muscle (vastii, rectus femoris) activation levels in individuals with crouch gait from CP, or knee extension deficiency from SB, iSCI or MD.	(1) This secondary endpoint is change in knee extensor muscle activation (vastii and rectus femoris) between two time points: initial outcome assessment and final outcome assessment which occur immediately before and after the 12-week community use block. The peak knee extensor activation levels will be measured by peak activation during the stride and the overall knee extensor activation will be measured by area under the normalized EMG curve. Evaluation of this objective will occur at the final outcome assessment, corresponding to visit 15 for Group A and visit 17 for Group B.<br><br>Children with knee extension deficiency typically have decreased peak knee extensor muscle activity. An optimal result would be achievement of increased peak knee extensor muscle activation during stance.	(1) Our previous research using the pediatric robotic exoskeleton has evaluated change in volitional EMG of knee extensor (vastii, rectus femoris) muscles during walking trials.
(2) To evaluate change in knee extensor muscle strength after 12-weeks of community robotic exoskeleton gait training in children with crouch gait from CP, or knee extension deficiency from SB, iSCI or MD.	(2) This secondary endpoint is change in knee extensor muscle strength, assessed as maximum isometric knee extension torque on the Biodex, between two time points: initial and final outcome assessments, which correspond to immediately before and after the 12-week community use block.	(2) Children with knee extension deficiency often have decreased knee extensor strength as an underlying factor. Our previous research has shown immediate increases in knee extensor muscle EMG when exoskeleton resistance is applied to knee extension; we expect repeated training at higher muscle activation levels to increase extensor muscle strength.
(3) To evaluate changes in average gait speed during overground walking as a result of robotic exoskeleton training	(3) This secondary endpoint is change in average gait speed evaluate between two time points: initial outcome assessment and final outcome assessment which occur immediately	(3) Our previous research using the pediatric robotic exoskeleton has shown that overground walking speed increases across visits with the

in children with crouch gait from CP, or knee extension deficiency from SB, iSCI or MD.	before and after the 12-week community use block. This endpoint will be assessed using the Vicon Motion Capture system as well as with the 6-min walk test. Evaluation of this objective will occur at the final outcome assessment corresponding to visit 15 for Group A and visit 17 for Group B.	NIH/Agilik exoskeleton. The feasibility study on the interleaved robotic exoskeleton mode found the resistance mechanism hinders initial progress on increasing gait speed, but increased muscle activation. Therefore, we expect gait speed to increase while walking without the exoskeleton to increase after the higher dosage of robotic exoskeleton in the community.
(4) To evaluate the primary endpoint at six weeks post completion of robotic exoskeleton training for persistence of improvement in peak knee extension during midstance in all participants who return for follow-up.	(4) This secondary endpoint will be knee extension range of motion assessed using change in peak knee extension during midstance between two time points: final outcome assessment and follow-up outcome assessment. The objective will be evaluated at the follow-up visit for each group, to occur six-weeks after completion of robotic exoskeleton training. This corresponds to visit 17 for Group A and visit 18 for Group B.<br><br>Previous use of the device in the clinical-setting based protocols has indicated the possibility of persistence of effect, but our research has not evaluated this phenomenon following the longitudinal use of the device. An optimal result would be persistence of improvement in peak knee extension during midstance after completion of therapy.	(4) Our previous research has supported the use of the pediatric robotic exoskeleton as a rehabilitative strategy but has not evaluated the use of the device in a longitudinal community-based setting until now. This objective serves to evaluate the persistence of effects over the course of a six-week follow-up from completion of robotic exoskeleton training.
(5) To evaluate any effect of the order of standard therapy and exoskeleton intervention on peak knee extension during midstance by comparing the primary endpoint between Group A and Group B.	(5) To control for time and order differences between groups, the primary endpoint (peak knee extension at midstance) will be assessed separately within Group A and Group B between two time points: initial outcome assessment and final outcome assessment which occur immediately before and after the 12-week community use block. Evaluation of this objective will occur at the final outcome assessment corresponding to visit 15 for Group A and visit 17 for Group B.<br><br>An optimal outcome would be no significant difference between groups.	(5) Our previous research has reliably used peak knee extension at midstance to evaluate improvement in knee extension. This objective will apply the primary endpoint within the two groups of a cohort to evaluate for effect of timing or order of standard therapy and exoskeleton intervention on any improvements found as a result of this protocol.
Tertiary/Exploratory		
(1) To evaluate change in peak knee extension at midstance as a function of exoskeleton intervention dosage by examining this parameter at multiple time points.	(1) This endpoint is peak knee extension at midstance. We will evaluate this objective between multiple time points as follows:<br>Baseline to Initial Assessment (12 weeks)<br>Baseline to Final Assessment (24 weeks)<br>Baseline to Follow-up Assessment (30 weeks)	(1) Our previous research has evaluated the pediatric robotic exoskeleton and observed significant improvements in knee extension over the course of a 3 to 8-week, 9 visit protocol within the clinical setting. This objective aims to quantify the expected improvement

	The Initial Assessment corresponds to visit 13 for Group A and visit 15 for Group B. The Final Assessment corresponds to visit 15 for Group A and visit 17 for Group B. The Follow-up Assessment corresponds to visit 16 for Group A and visit 18 for Group B.<br><br>An optimal outcome would be a significant improvement in knee extension immediately following completion of the intervention block that increases with higher dosage and persists at six weeks follow up.	in knee extension as a result of a much higher dosage, spanning 6 weeks in the clinical setting and 3 months of daily use in the community.
(2) To evaluate change in knee extensor and flexor muscle spasticity following use of a robotic exoskeleton in children with crouch gait from CP, or knee extension deficiency from SB, iSCI or MD.	(2) This endpoint is change in flexor and extensor muscle spasticity between two time points: initial outcome assessment and final outcome assessment which occur immediately before and after the 12-week community use block. This will be measured using the Modified Ashworth and Tardieu spasticity scales. This will be evaluated at the final outcome assessment corresponding to visit 15 for Group A and visit 17 for Group B.<br><br>In children with lower-limb muscle weakness resulting in knee extension deficiency, there can be co-activation of agonist and antagonist muscles to knee extension which inhibits proper gait mechanics. The optimal result of robotic exoskeleton training would be to inhibit or reduce spasticity of antagonist knee extensor muscles. Muscle spasticity will be measured by the modified Ashworth scale and the Tardieu scale. This will occur at visit 15 for Group A and visit 17 for Group B, but will be analyzed together. Analysis of spasticity will be an exploratory aim because we have not previously evaluated spasticity before and after exoskeleton gait training.	(2) Traditional bracing to compensate for muscle weakness or spasticity by providing passive support or blocking unwanted motion can often lead to greater weakness in muscles over time due to disuse atrophy (Rogozinski, Davids, Davis, Jameson, & Blackhurst, 2009). Our prior research study evaluated the robotic exoskeleton as an alternative training strategy to preserve or augment strength over a continual rehabilitation training strategy and showed initial safety and feasibility of the device's use. Here we will examine the effect on muscle spasticity for the first time.
(3) To evaluate improvement in gross motor function immediately following completion of robotic exoskeleton training period in children with crouch gait from CP, or knee extension deficiency from SB, iSCI or MD.	(3) This endpoint is change in gross motor function evaluated between two time points: initial outcome assessment and final outcome assessment which occur immediately before and after the 12-week community use block. This is measured using two validated functional instruments, GMFM-66 and PEDI-CAT, which are described in Table 5. The objective will be evaluated at the final outcome assessment which corresponds to visit 15 for Group A and visit 17 for Group B.<br><br>Children with knee extension deficiency have a characterizable decrease in gross motor function during overground walking. An optimal result would be to observe an	(3) The GMFM-66 PEDI-CAT are validated clinical scales of function to classify pediatric patients with knee extension deficiency by gross motor function. Here we will explore the effect of robotic exoskeleton gait training on participant scoring on these scales.

	improvement in gross motor function classification immediately following the 12-week intervention period.	
(4) To evaluate the safety and feasibility of a community-based protocol for rehabilitation using a pediatric robotic exoskeleton among children with crouch gait from CP or knee extension deficiency from SB, iSCI or MD.	(4) This endpoint is a measure of safety and compliance to the protocol by pediatric participants. Compliance will be assessed at the completion 12-week community use block (corresponding to visit 15 for Group A and visit 17 for Group B) by determining whether they met the suggested dosage of device use while in the community.<br><br>Safety will be assessed at the completion of the study for each participant by evaluating the occurrence of any adverse events. Each participant will be evaluated for potential side effects falling under two categories; (1) minor/anticipated or (2) serious/unanticipated. Please refer to Section 2.3.1.k for a list of potential side effects as a result of training with the P.REX/Agilik. Safety will be evaluated throughout the duration of the study, but this safety objective will be assessed at the final visit for each participant, corresponding to visit 17 for Group A and visit 18 for Group B.	(4) Our previous research implementing the pediatric robotic exoskeleton in the clinical setting showed the initial feasibility and safety of its use by the target population. The device is now classified as a class I device and is deemed of minimal risk to users by the FDA. Nevertheless, we will explore feasibility and safety of the device during use outside the clinical setting.

4 STUDY DESIGN

4.1 Overall Design

This is a study of pediatric outpatients with crouch gait from cerebral palsy (CP) or knee extension deficiency from another neuromuscular disorders such as muscular dystrophy (MD), spina bifida (SB), and incomplete spinal cord injury (iSCI). Individuals with CP walk in a crouched posture due to multiple impairments with motor control deficits being an important factor (Steele, Seth, Hicks, Schwartz, & Delp, 2010), whereas patients with SB, MD or iSCI tend to exhibit gait pathology as a result of muscle weakness (Sandler, 2010; Doglio, et al., 2011; Jayaraman, et al., 2006). Our study design will account for these identifiable differences underlying the mechanism of gait pathology, by separating participants by diagnosis into two subject pools for analysis: the CP subject pool and the Neuromotor Disorder subject pool (consisting of patients with SCB, iSCI or MD). These two subject pools will complete the study design independent of each other including separate randomization into Groups A and B following inclusion in the protocol at visit 0. Both the CP and Neuromotor Disorder subject pools will complete the same study design detailed in this protocol, but will be analyzed independent of each other.

The aim of the study is to further investigate the safety and effectiveness of a robotic exoskeleton for overground gait training targeting improvements to knee angle profile, posture, and gait speed. As previous studies at the NIH have demonstrated the orthotic effects and immediate benefits of an extension-assist robotic exoskeleton on the target population of children with CP (protocol #13-CC-0210), this study will investigate longer-term benefits using this technology

outside of the clinical setting and in application to a broader patient population including children and adults with knee extension deficiency from a diagnosis of SB, MD or iSCI.

We hypothesize that use of a robotic exoskeleton for 1 hour/day, 5 days/week over a 12-week training period will lead to functional improvements in overground walking without the exoskeleton by (i) increasing peak knee extension angle, (ii) improving overground gait speed, and (iii) improving knee extensor, including the vastus lateralis and rectus femoris, muscle activation and strength. In addition, we hypothesize that regular use of a robotic exoskeleton over a 12-week training period will also improve volitional gait speed and the observed functional improvements in overground walking will persist following completion of the exoskeleton intervention training.

This study is a single site outpatient study with testing to occur at the National Institutes of Health Clinical Center, Rehabilitation Medicine Department, Neurorehabilitation and Biomechanics Section. This is a study of a novel device. It is a pivotal study, analogous to phase 1, designed to assess both safety and effectiveness of the intervention. No sub-studies will be included in this protocol.

The robotic exoskeleton used as the intervention in this study consists of two orthotic braces, one for each limb, with each device containing a single robotically actuated degree of freedom at the sagittal plane knee joint (flexion/extension direction) and the associated embedded electronics and sensors to power and control the device. Additionally, and optionally, the robotic exoskeleton can incorporate a commercially available surface electronic stimulation device for stimulation of lower extremity muscles; the activation and timing of the electrical stimulation is integrated with the embedded control system of the exoskeleton.

The study intervention device has 6 possible control modes for each participant (Table 4). With the ultimate intervention goal being increased volitional function following device use outside the clinical setting, our primary focus will be to establish a resistive or interleaved assistive/resistive mode for each patient to use for training to aid in rehabilitation of the knee extension deficiency. We aim to secondarily establish at least one assistive-only mode for training in the case the patient cannot operate under the resistive mode for the prescribed dosage of training.

To ensure safe operation and identify the optimal operational mode for each participant, a 10-visit accommodation/in-lab training period is included at the beginning of the intervention period for each group. Prior to this in-lab training period, a baseline gait analysis will be performed using motion capture and EMG. The initial operational mode for each participant will be identified based on analysis of the biomechanical results during this baseline assessment. The device will then be fit, adjusted and calibrated at the subsequent exoskeleton walking practice visit, and throughout the following 10 accommodation/in-lab training sessions if necessary. A primary mode, and up to three secondary modes, of operation will be determined for the robotic exoskeleton for each participant, as described previously. When the device is taken home for community use, the participant will utilize their primary and secondary mode(s) for exoskeleton training, with a target of 1 hour/day, 5 days/week. The control parameters for each of these modes will be preset before the device is sent home. While the participants will not be required to fine tune the device outside of the clinical setting, adjustments are possible through the tablet-based software that operates the exoskeleton. Any adjustments to these settings will be discussed

with our research staff, who will check in regularly with the participants during the community use portion of the study. Additionally, all participants will be given the option to visit our clinical lab to facilitate adjustments to the device settings if needed. These adjustments may also take place during the intermediate outcome assessment visits.

Table 4. Robotic Exoskeleton Possible Conditions

Condition Number	Joint Mechanism		
	Knee	Knee Extensor FES Applied	Ankle
1a	Assistive Torque	No	Typical* (free or locked)
1b		Yes	
2a	Resistive Torque	No	
2b		Yes	
3a	Interleaved Assist/Resist	No	
3b		Yes	

*Typical indicates that the ankle mechanism will be set to either free or locked based on the daily walking condition of the user, or alternatively, the mode that is the safest and most effective for use with the exoskeleton as identified during clinical gait analysis. There will only be one ankle mode used for the duration of the study.

The design of the study will be structured as a randomized, crossover study. The target intervention dosage will be constant across all participants (5 days/week, 1 hour/day). A schematic of the study design is represented in Figure 1 for reference. For all participants, visit 0 will consist of consent/assent, eligibility evaluation, review of medical history and physical examination which will take up to 4 hours. After the cohort is established, the participants will be randomly placed into one of the two study arms of this protocol with no stratification: (i) exoskeleton intervention start, deemed Group A or (ii) control start, deemed Group B. All participants will then return for visit 1, to gather initial gait and functional mobility assessments. Optionally, visit 1 can take place on the same day as visit 0. This is a multi-visit study with outcome assessments of the same measures at various points in the protocol. Each time outcome measures are referenced, it is referring to the list of measures outlined in Table 5. One of the functional scales, GMFM-66, will only be evaluated at the Baseline Assessment, the Initial Outcome Assessment and the Final Outcome Assessment following completion of exoskeleton use (visit 15 for Group A and visit 17 for Group B), but not during any other outcome assessments. If the spasticity outcome measures (modified Ashworth and Tardieu scales) indicate spasticity is not present at baseline, they will not be repeated at subsequent assessments. Additionally, if the participants do not complete more than half of the 10 in-lab accommodation sessions, only motion capture and EMG outcomes will be assessed at the Initial Outcome Assessment. At the Intermediate Outcome Assessment after 6 weeks of the 12-week community use block, the participant will optionally, at the discretion of the study clinicians, undergo assessment of the functional scales including the PEDI-CAT, GMFM-66, the 6-minute walk test, the Timed Up and Go, the Modified Ashworth and Tardieu Scales, and the isometric test on the

Biodex dynamometer. This Intermediate Assessment will still require the participant to wear the motion capture markers and EMG sensors during walking trials to allow for the calculation of the first nine parameters in Table 5 below.

Table 5. Outcome Measures

**Outcome Measure**	**Description**	**Units**
Peak knee extension	Minimum knee angle during stance phase	Degrees
Knee extension at initial contact	Knee at foot contact	Degrees
Step length	The distance covered by one step	Meters
Gait speed	Mean overground velocity	Meters/sec
Knee moment	Net torque about the knee joint during stance phase	Nm
Peak activation of knee extensor muscles	Knee extensor muscles = *vastus lateralis, vastus medialis, vastus intermedius, rectus femoris*<br>Maximum muscle activity normalized by resting value within the stride	Normalized recordings (mV/mV)
Peak activation of knee flexor muscles	Knee flexor muscles = *semitendinosus, semimembranosus*<br>Maximum muscle activity normalized by resting value within the stride	Normalized recordings (mV/mV)
Mean (via area under the curve) activation of knee extensor muscles	Numerical integration of the normalized EMG curve over the stride	Normalized recordings (mV/mV)
Mean (via area under the curve) activation of knee extensor muscles	Numerical integration of the normalized EMG curve over the stride	Normalized recordings (mV/mV)
Gross Motor Function Classification System (GMFCS)	Categorization system of gross motor skills of children with CP	5 levels<br>(I, II, III, IV, V)
Pediatric Evaluation of Disability Inventory-Computer Adaptive Test (PEDI-CAT)	Computer assessment completed by participant (or parent) to assess ability in three functional domains: daily activities, mobility and social/cognitive.	Scaled score range of 20-80
Gross Motor Function Measure (GMFM-66)	A quantitative clinical scale to assess five dimensions of lower extremity functioning with total of 66 items	Each item scored (0-3), total score range (0-198)
6-minute walk test (6MWT)	Total distance covered during overground walking for 6 minutes	Meters
Timed up and go	Time to move from seated position, walk 10 feet, turn, walk 10 feet back to chair, and sit	Seconds

Modified Ashworth Scale	Tests resistance to passive movement about a joint against varying velocities	Score range: 0, 1, 1+, 2, 3, 4
Tardieu Scale	Measures spasticity by accounting for resistance to passive movement at both slow and fast speeds	Full range of motion and angle of catch at fast and slow speeds
Isometric Test on Biodex Dynamometer	Performed at full extension and 30 degrees of flexion	Nm

Group A will also undergo digital casting for their exoskeleton fitting at visit 1. After visit 1, each study arm will begin the first of three 12-week blocks. Group B will continue with any conventional physical therapy they are currently receiving for the first 12-week block. Group B will be required to return at the halfway point, 6 weeks before the finish of the first 12-week block to undergo digital casting for their exoskeleton fitting. It is imperative Group B wait until this point of their standard therapy 12-week block to ensure the custom device fits appropriately for when they crossover and participate in the exoskeleton intervention portion using their custom orthotic at the end of the first block.

Fabrication of the custom orthotic components of the exoskeleton for each participant requires approximately 4-6 weeks. Upon manufacturing completion of the custom brace, Group A will return for visit 2 around week 6 of their first 12-week block. This visit will consist of an initial setup, tuning, and testing of the exoskeleton during walking, lasting a maximum of 4 hours. During the tuning, an individual’s exoskeleton settings will be determined to best fit the individual’s needs and comfort, including the extension-assist control mode, level of extension assistance, and electrical stimulation waveform parameters such as amplitude, frequency, and pulse width (if applicable to the participant). Group A participants will then spend visits 3-12 over up to 5 weeks returning to the facility for exoskeleton walking practice to ensure each participant can safely execute overground walking with the device. During this portion of the intervention, all Group A participants will also complete an instruction for at-home use and care to prepare for their next 12-week block. This instruction will include putting on and taking off the exoskeleton, charging, storage, controlling, and cleaning. Before participants will be allowed to take the device home for use, the following criteria will need to be met:

i. Participants will have completed a maximum 10-visit exoskeleton accommodation and training period in our facility. A skill level safe for at home and community use will be assessed by the medically responsible investigator (physician), the treating physical therapist and the principal investigator.
ii. Participants are required to have a caregiver attend the accommodation/in-lab training sessions with them to learn alongside the trained therapists. This allows the caregiver to be fully trained in the skillset required to safely and effectively operate the exoskeleton independently outside of the clinical setting. This skillset will include the ability to monitor donning and doffing the device, sit-to-stand, and walking, as well as proper care and storage as described in the risk analysis (Section 2.3.1 c-e).

An additional 2 weeks of in-lab training will be allowed, if necessary to allow scheduling of up to 10 visits for this portion of the protocol. This extension will only be utilized in the event of scheduling difficulties. Ultimately, it will be the decision of the medically-responsible

investigator (MRI) and the principal investigator (PI) to sign-off on use of the device outside of the lab.

Following an adequate accommodation/in-lab training period (visits 3-12), Group A participants will return for visit 13 to undergo an outcome assessment with and without their exoskeleton. The outcome assessment measures are described in Table 5 above. This assessment will include motion capture and EMG outcome measures for all participants. It will include the other outcome measures in Table 5 if more than half of the 10 in-lab visits were completed. This will include the GMFM-66 to accommodate the secondary objective.

To finish the 12-week standard therapy intervention block for Group B, participants will return for visit 3 to undergo the same outcome assessments as Group A detailed above, only under the condition of without their exoskeleton.

At this point, a second 12-week intervention block will initiate for both groups. Group A will have the option to take their custom brace and exoskeleton home for at-home implementation where subjects will be instructed to use their robotic exoskeleton a minimum of 5 days per week for a minimum of 1 hour per day with their primary mode of operation as previously established during the in-lab portion of the training. If they are unable to complete the specified dose in the primary training mode (that will be targeted to be the resistive mode), they will be permitted to complete any remaining time with the secondary mode(s) which either combine assistance and resistance or are assistive. Group A participants who decide to participate in the community exoskeleton use will be deemed Group A1 and Group A participants who elect to not take their custom exoskeleton home (or are not permitted to do so based on the decision of the MRI or PI) will be deemed Group A2.

In the case a participant decides not participate in the community use portion, they will not bring the device home and will skip the community use block resulting in a return to standard therapy and earlier study completion. In this case, A2 participants will be asked to return for a Follow-up Outcome Assessment in 6 weeks and a final Standard Outcome Assessment in 12 weeks to complete the final assessment in the standard therapy block. All outcome measures listed in Table 5 above will be assessed at both the Follow-up and Standard Outcome Assessments, but only without their robotic exoskeleton.

All Group A1 participants will return to our facility for visit 14 after approximately 6 weeks following the start of this 12-week community intervention block to undergo an Intermediate Outcome Assessment. As previously described, the first nine outcome measures in Table 5 above will occur at this visit, with the optional (at the discretion of the study clinician) addition of the remaining outcome measures (See Table 5 above). At this assessment, Group A1 will test both with and without their exoskeleton. All Group A1 participants will return to our facility for visit 15 after approximately another 6 weeks (12 weeks of total intervention) to undergo a Final Outcome Assessment (see Table 5 above). At this Final Outcome Assessments, Group A1 will test with and without their exoskeleton.

All Group A1 participants will then enter their third and final 12-week block consisting of only standard therapy, without the exoskeleton, in the community, indicating the point where Group A participants have now crossed over from the intervention to the control arm. Group A1 will return approximately 6 weeks after initiation of this 12-week block to complete the Follow-Up assessment (see Table 5) without the exoskeleton. Group A1 will return another 6 weeks later to

complete their final Standard Outcome Assessment (see Table 5) without their exoskeleton, marking completion of the 12-week standard therapy intervention.

Group B will have returned (visit 2) for a digital casting for their custom exoskeleton orthotics at approximately week 6 of their first 12-week block to ensure enough time for fabrication of their custom components. Therefore, they may return for visit 4 at the start of their second 12-week block with a custom orthotic exoskeleton already complete. This visit will mark the start of Group B involvement with the intervention device, therefore indicating a crossover. Visit 4 will consist of an initial setup, tuning, and testing of the exoskeleton during walking, lasting a maximum of 4 hours. This will be followed by visits 5-14 over a course of 5 weeks for exoskeleton walking practice to ensure each participant can safely execute overground walking with the device and to complete instruction for at-home use and care including putting on and taking off the exoskeleton, charging, storage, controlling, and cleaning. Before participants will be allowed to take the device outside of the clinical setting, they must meet the same criteria as described for Group A at this point in the study.

In the same fashion as Group A, following an adequate accommodation/in-lab training period, Group B participants will return for visit 15 to undergo an outcome assessment with and without their exoskeleton (see Table 5 above). This assessment includes an evaluation of the GMFM-66 to accommodate the secondary objective. Group B participants will then have the option to take their custom brace and exoskeleton home for at-home implementation where subjects will wear the robotic exoskeleton a minimum of 5 days per week for a minimum of 1 hour per day. Dosage instructions will be the same as for Group A. Group B participants who decide to participate in the community exoskeleton use will be deemed Group B1 and Group B participants who elect to not take their custom robotic exoskeleton home will be deemed Group B2.

As mentioned previously for Group A2 participants, in the case a Group B participant decides not participate in the community use portion, they will skip the community use block resulting in a return to standard therapy and earlier study completion. In this case, all B2 participants will be asked to return for a Follow-up Outcome Assessment in 6 weeks and a final Standard Outcome Assessment in 12 weeks to complete the final assessment in the standard therapy block. All outcome measures listed in Table 5 above will be assessed at both the Follow-up and Standard Outcome Assessments described here, but only under the condition of without their robotic exoskeleton.

All Group B1 participants will return for visit 16 approximately 6 weeks after initiation of their community exoskeleton use for an Intermediate Outcome Assessment. As previously described and to mirror Group A1, the first nine outcome measures in Table 5 above will occur at this visit, with the optional (at the discretion of the study clinician) addition of the remaining outcome measures (See Table 5 above). At this assessment, Group B1 will test both with and without their exoskeleton.

All Group B1 participants will return for visit 17 after approximately another 6 weeks from their previous visit to undergo a Final Outcome Assessment (see Table 5 above) at the completion of the final 12-week block. Group B1 will test with and without their exoskeleton. A final Follow-Up Outcome Assessment without their exoskeleton will occur for all Group B1 members at visit 18, approximately 6 weeks after completion of the final 12-week intervention block.

In summary, Group A and Group B participants will undergo the same outcome assessments and dosage of the intervention, but have been organized into a cross over study to randomize the order of intervention and control. This study design aims to minimize any potential effect the child's developmental stage could have on the results. This is a valid consideration for a nine-month research study involving children who could experience physical and physiological changes over a time period of nine months.

No criteria of interim analyses should lead to the early termination of an intervention period. To minimize bias in the baseline, interim, and post-treatment analyses, spatiotemporal gait parameters and gait kinematics will be analyzed by laboratory researchers blinded to the participant's exoskeleton mode of operation.

4.2 Scientific Rationale for Study Design

The study design was chosen to be structured as a crossover study in order to maximize participation in the study. With this type of design, all participants will participate in the study intervention (i.e. all participants will have the opportunity to use their own custom exoskeleton for gait training). The crossover design also allows for the investigation of any loss of improvement following the 12-week exoskeleton intervention by following Group A through 12-weeks of standard therapy after completion of their exoskeleton therapy.

Continuation of the participants' existing standard physical therapy was chosen as the control for this study as it is widely-used by the target populations.

4.3 Justification for Dosage

As mentioned, a previous high-dosage gait training study investigated the 6-minute walk test and the ABILOCO functional mobility measure from intensive bilateral upper- and lower-limb training of approximately 80 hours compared with the same dose of conventional therapy (Bleyenheuft, Arnould, Brandao, Bleyenheuft, & Gordon, 2015). The goal of our study is to emphasize activity and gait training with the exoskeleton resistance and/or assistance with the highest possible dosage while allowing for some flexibility within groups for individuals' comfort of usage. The dosage of exoskeleton gait training treatment will be for a minimum of 5 days per week for a minimum of 1 hour per day during the 12-week community use exoskeleton intervention. This will allow for at least 84 hours of total exoskeleton gait training, while emphasizing regular use for a 3-month time period. The goal is to maximize total training time during the exoskeleton intervention for each individual participant.

5 STUDY POPULATION

5.1 Inclusion Criteria

In order to be eligible to participate in this study, an individual must meet all of the following criteria:

1. Provision of signed and dated separate informed consent and assent forms for screening purposes. Upon inclusion in the protocol, provision of signed and dated informed consent and assent forms to begin participation in the study will be necessary.
2. Stated willingness to comply with all study procedures and availability for the duration of the study, or alternatively, ability to do so based on parent report and physician observation during history and physical examination.

3. Age 3 to 17 years old.
4. Have a gait pathology involving the knee joint, from a diagnosis of cerebral palsy, muscular dystrophy, spina bifida, or incomplete spinal cord injury.
5. Knee joint range of motion of at least 25 degrees in the sagittal plane (knee extension/flexion) assessed with hip extended in supine position. Hamstring contracture as assessed by straight leg raising test does not limit ability to participate in the study.
6. Ankle joint range of motion of at least 15 degrees in the sagittal plane (dorsi-plantar flexion) with the foot in neutral alignment.
7. A measured foot-thigh angle of -15 to 30 degrees in prone position.
8. Able to walk at least 10 feet without stopping with or without a walking aid.

5.2 Exclusion Criteria

An individual who meets any of the following criteria will be excluded from participation in this study:

1. Any neurological, musculoskeletal or cardiorespiratory injury, health condition, or diagnosis other than cerebral palsy, muscular dystrophy, spina bifida, or incomplete spinal cord injury that would affect the ability to walk as directed with the robotic exoskeleton.
2. A history of uncontrolled seizure in the past year.
3. Pregnancy. A urine test will be performed for all participants who are able to become pregnant at the initial screening visit and in the case of a positive test, the participant will be excluded from participation. Further monitoring will rely on self-reporting of interruption in menstruation that would require re-testing for pregnancy at the next visit.
4. Any acute cardiopulmonary condition which limits exercise to less than 60 minutes per session or less than 5 days per week.

5.3 Inclusion of Vulnerable Participants

5.3.1 Participation of Children

This study will involve children who have not attained the legal age for consent to treatments or procedures involved in the research. This protocol will act according to NIH HRPP Policy 402 to ensure sufficient information is provided as well as appropriate practices are in place to minimize the children's susceptibility to undue influences or unnecessary risks (physical, psychological, etc.) as research subjects. This will include, but is not limited to, obtaining parental permission and child assent. In the case a child is the responsibility of parents who share joint custody, we will require both parents to give their permission as stated in NIH policy. If the child reaches age of majority during their participation in the study, we will seek legally effective informed consent for the child to continue with the study intervention, now under the classification of adult.

The target population of this study is children between the age of 3-17 years old. We plan to specifically target children with a neurological disorder, such as cerebral palsy, because the associated gait pathology is known to progress toward loss of ability to walk with age. Currently available treatments, such as physical therapy, botulinum toxin injections, and or surgery may lead to short term improvements, but despite these interventions an individual's ambulation is likely to decline over time and/or they may lose the ability to ambulate by the time they are adults (Wein, Bryant, & Hicklin, 2017;

Lerner, Damiano, & Bulea, 2017a). This study will be used to determine the efficacy of exoskeleton gait training in the community i.e. outside of the clinical laboratory setting which makes this mode of intervention more accessible to children in our target population. Our previous study has already shown the feasibility and safety of using this novel wearable technology in gait training for children. Study participants had immediate improvements in knee kinematics while wearing the device, supporting the efficacy of this device in the clinical setting (Bulea et al., 2022; Bulea, Lerner, & Damiano, 2018; Lerner, Damiano, & Bulea, 2016, 2017a, 2017b; Lerner, Damiano, Park, Gravunder, & Bulea, 2016).

5.3.2 Participation of Children of NIH Staff

Children of NIH staff, excluding study team family members, may be enrolled in this study as long as this population meets the study entry criteria. Neither participation nor refusal to participate as a subject in the research will have an effect, either beneficial or adverse, on the participant's employment or position at NIH.

Every effort will be made to protect participant information, but such information may be available in medical records and may be available to authorized users outside of the study team in both an identifiable and unidentifiable manner.

The *NIH Frequently Asked Questions (FAQs) for Staff Who are Considering Participation in NIH Research* will be made available. Please see section **10.1.3** for consent of NIH Staff.

5.4 Inclusion of Pregnant Women, fetuses or neonates

Not applicable.

5.5 Lifestyle Considerations

Not applicable.

5.6 Screen Failures

Screen failures are defined as participants who consent/assent to participate in the clinical trial but are not subsequently assigned to the study intervention or entered in the study. A minimal set of screen failure information is required to ensure transparent reporting of screen failure participants, to meet the Consolidated Standards of Reporting Trials (CONSORT) publishing requirements and to respond to queries from regulatory authorities. Minimal information includes demography, screen failure details, eligibility criteria, and any serious adverse event (SAE).

Individuals who do not meet the criteria for participation in this trial (screen failure) because of an insufficient knee joint range of motion, ankle joint range of motion or foot-thigh angle or inability to walk at least 10 feet without stopping may be rescreened. Rescreened participants should be assigned the same participant number as for the initial screening. We require a new informed consent and assent and six months before another clinical trial screening for a participant to attempt to participate again following a screen failure.

5.7 Strategies for Recruitment and Retention

The maximum anticipated number of participants to be screened for this study is 44 participants composed of two subject pools, each with 22 individuals. The first subject pool includes children with cerebral palsy. The second subject pool includes children with muscular dystrophy, spina bifida, or incomplete spinal cord injury. There will be no discrimination by gender or race. The age range of the target population in both subject pools is 3 to 17 years old. The target study sample size is 13 individuals in each subject pool, totaling 26 participants in the overall study sample size. This number accounts for the expectation that 80% of participants will complete the study through the Final Outcome Assessment, and 25% of participants will complete the initial, in-lab phase of the study but elect not to complete the community use portion of the study, which is necessary for analysis of the primary and secondary objectives.

This is a single site study with an outpatient component whereby the intervention is performed in the community, which can include the home, school, or other location (i.e., gym, during physical therapy sessions, etc.). We anticipate participants to be enrolled from the U.S and internationally. A flyer has been created for recruitment information (see Appendix Document C). The flyer briefly describes the study, the inclusion and exclusion criteria, and whom to contact for more information. In order to recruit participants for this study, we will utilize the NIH list-serv to spread awareness of the clinical trial within the NIH community and its networks. We also plan to inform community physicians who may treat our target population of the potential enrollment of their own patients into the study if they meet study inclusion criteria. To do so, the recruitment flyer will be mailed to the offices of clinical care providers, physicians or physical therapists in the area. Subjects already known through participation in current or past protocols who have expressed interest in future studies and may qualify for this protocol may be contacted. We may also recruit patients who have expressed interest in the past in participating in our research protocols but who did not meet the eligibility criteria for those or were unable to come in during the study period. In addition, we will utilize the NIH Office of Patient Recruitment to advertise the study on appropriate social media outlets including YouTube, Facebook, and Twitter. We will also post flyers and advertisements on the pages of special interest groups of the same social media sites for individuals with cerebral palsy, spina bifida, incomplete spinal cord injury, and/or muscular dystrophy. Also, the advertisement flyers may be displayed throughout hospitals, physicians' offices, rehabilitation settings, in the community (i.e., churches, local support groups), or posted in local newspapers after permission for posting is obtained.

Prospective patients will be contacted by phone and/or email following their interest in participating. Upon inquiring about the study, potential candidates can be screened over the phone for basic components of the inclusion criteria such as diagnosis of CP, SB, MD, or iSCI. To the extent necessary, current medical records of the participant may be reviewed before bringing the potential candidate into the NIH facility for the eligibility evaluation. For pre-screening, inclusion/exclusion criteria will be reviewed with the parent/guardian of each prospective participant using the pre-screening phone script (see Appendix Document D).

The company that built the robotic exoskeleton under our current CRADA, and which will serve as the intervention device in this study, has created a brochure that details the device and its component parts. This brochure will be made available to all potential candidates, as well as provided for all participants who continue through the community use portion of the study.

This study will require long-term participation of patients. To enhance participant retention, the research staff will check in with participants and families weekly to ensure they are able to operate the device safely and that it is working as intended.

Owing to the functionally progressive nature of crouch gait in children, we will permit participants with cerebral palsy and other movement disorders to reenroll in this study after a minimum of 1 year of time has passed since the completion of their prior participation.

5.7.1 Costs

The participant will not be held responsible for any costs associated with their enrollment and participation in this study, including the cost of the custom exoskeleton device. If the participant experiences any emergent and/or unexpected complications due to their use of the exoskeleton outside of NIH, they will be advised to seek immediate care at a non-NIH facility and will be responsible for any incurred costs from such visit. If the participant experiences any non-emergent complication while using the exoskeleton in the community, they will be advised to return to NIH for an evaluation by an NIH clinician. If the participant requires treatment outside of the scope of care of the NIH physician or due to an issue unrelated to study participation, the patient should refer to an external provider, of which the costs will be the responsibility of the participant.

5.7.2 Compensation

Parents will not be compensated for their time and effort; all compensation will be paid to the participant. Children/participants will be paid for time and research-related inconveniences for each visit to the NIH Clinical Center as follows: $20 for the first hour, and $10 for all additional hours, for a maximum of $50 per day. Each visit will last no longer than 4 hours. Participants will also receive compensation each visit for the inconvenience of using functional electrical stimulation ($20), wearing the P.REX/Agilik ($20), using the motion capture software ($10) and using the muscle activation sensors to participate in the EMG assessment ($10). The minimum possible compensation for participation is $20, should the participant or investigator choose to stop the study within the first hour. Since each type of visit has slightly different parameters, the maximum compensation per day varies as follows:

- **$20 for the initial screening visit** (visit 0, 1 hour for review and signing of screening consent and assent forms and evaluation of inclusion and exclusion criteria to determine eligibility)
- **$70 for the initial baseline assessment** ($50 for 4 hours of participation, $10 for motion capture, $10 for EMG)
- **$80 for the initial P.REX/Agilik set up and walking practice** ($50 for 4 hours of participation, $10 for motion capture, and $20 for wearing the P.REX/Agilik)
- **$90 for each accommodation/in-lab training session wearing the P.REX/Agilik** ($50 for 4 hours of participation, $20 for wearing the P.REX/Agilik, $20 for using FES)
- **$110 for each outcome assessment visit** ($50 for 4 hours of participation, $20 for wearing the P.REX/Agilik, $10 for motion capture, $10 for EMG, $20 for using FES)

- **$70 for outcome assessment following standard therapy and follow-up assessment** (without exoskeleton) ($50 for 4 hours of participation, $10 for motion capture, $10 for EMG,)

The maximum possible compensation following inclusion in the study and provision of consent and assent, to include completion of visit 1 through visit 17-18 (depending on Group assignment), includes $1,520 per participant.

Participants will not be compensated for using the robotic exoskeleton in the community. All participants will be provided with their custom orthotic components of the P.REX/Agilik at the conclusion of their participation.

The participant will be paid by setting up a direct deposit or U.S. debit card depending on participant preference after each visit. We will collect a personal email address in order to set up the secure payment.

If you are unable to finish the study, you will receive a prorated amount of the total compensation for the parts you completed. If you have unpaid debt to the federal government, please be aware that some or all of your compensation may be automatically reduced to repay that debt on your behalf.

With few exceptions, study compensation is considered taxable income that is reportable to the Internal Revenue Service (IRS). A "Form 1099-Other Income" will be sent to you if your total payments for research participation are $600 or more in a calendar year.

No travel compensation will be provided. Our target population is classified as minors, aged 3-17. There is no escort payment for parents.

6 STUDY INTERVENTION

6.1 Study Interventions(s) Administration

6.1.1 Study Intervention Description

The use of the P.REX/Agilik Exoskeleton in this study is considered investigational as it is not approved by the FDA to treat crouch gait or knee extension deficiency. The definition of a Significant Risk (SR) Device (21CFR812.3(m)) is one that:

a) is intended as an implant and does not present a potential for serious risk to the health, safety, or welfare of a subject,
b) is not purported or represented to be for use supporting or sustaining human life and does not present a potential for serious risk to the health, safety, or welfare of a subject,
c) is for a use of substantial importance in diagnosing, curing, mitigating, or treating disease, or otherwise preventing impairment of human health and presents a potential for serious risk to the health, safety, or welfare of a subject
d) otherwise present a potential for serious risk to the health, safety, or welfare of a subject

The use of the P.REX/Agilik in this protocol does not meet the above criteria for a SR device, and therefore supports a non-significant risk (NSR) determination for use in this protocol. Specifically, the P.REX/Agilik exoskeleton is not an implanted device (a) nor is

it intended for use in supporting or sustaining human life (b). The device will be used to treat and potentially improve a gait pathology due to a neuromuscular disorder, however its use does not present a potential for serious risk to the health, safety, or welfare of a subject. This determination is supported by our clinical experience and experimental results using the P.REX/Agilik exoskeleton, both in its current form and its prior generations, for multiple years in our clinical laboratory under our existing IRB approved protocol (#13-CC-0210). This includes use of the device during overground walking and walking on a treadmill at various speeds in children as young as 5 years of age. There have been no adverse events regarding the safety of the device under this protocol nor has there been any safety incidents or other issues which would present a risk to the health or welfare of our participants. The use of the exoskeleton device under the existing IRB approved protocol #13-CC-0210, including the active motorized assist joint module and the controllable resistance module (formerly called the PowerWalk® by Agilik Technologies, now named Agilik®) was reviewed by the FDA and was determined be non-significant risk (see Appendix Document B). Further, since approval of the existing protocol using the Agilik® device (#13-CC-0210), it has been registered by Bionic Power and approved by the FDA as a Class I device. We note that this device is not yet commercially available. The device will be labelled “For investigational use only” as required under the abbreviated IDE requirements for NSR device studies.

The current protocol proposes to use the device during walking both in the clinic environment and also in the home or community environment. We will take multiple steps to ensure the risk of P.REX/Agilik exoskeleton use does not significantly increase when outside the laboratory setting. Prior to use outside the clinical laboratory, all participants will complete up to 10 total visits using the device within the clinical setting. The PI and study physician will assess the safety of the device use outside of the clinical setting before each participant can proceed with use in the home/community use. All subjects may also opt out of using the device in the community/home if they do not wish to participate in this component of the protocol. All subjects participating in the device use outside of the clinical setting will be properly instructed on at-home use and care, which will include donning and doffing the exoskeleton, operating the device through the wireless interface, charging, storage, and cleaning. This instruction will occur during the 5-week accommodation/in-lab training period consisting of up to 10 visits.

The design details of the NIH pediatric robotic exoskeleton (P.REX/Agilik) have been previously described (Bulea, Molazadeh, Thurston, & Damiano, 2022; Chen, et al., 2021). Briefly, the P.REX/Agilik design is based on a knee-ankle-foot orthosis with a single actuated degree of freedom in the sagittal plane at the knee and a passive ankle joint. An encoder is integrated with the actuator to measure knee angle and angular velocity via differentiation of motor position. The applied motor torque is computed from current. The actuator is mounted to an individually customized orthosis (Ultraflex Systems) with two attachment points (each with a minimum of two fasteners) on the lateral thigh and one attachment point (3 fasteners) between the drive arm of the actuator and the shank upright. The attachment points are customized to center the actuator on the knee at the lateral femoral epicondyle. The footbed contains an exterior shell and, optionally, a supramalleolar orthosis (SMO) insert. A force sensitive resistor (FSR, Interlink Electronics, Westlake Village, CA) is placed between the SMO and exterior

shell extending from the heel to the base of the metatarsal bones to measure ground contact and toe-off. Embedded electronics in the upper portion of the actuator contain a microprocessor and associated circuitry for real-time collection and processing of sensor data and control of motor torque output.

The motor, sensors and onboard circuitry are powered by a lithium ion battery which can either be carried on a pack on the torso for untethered operation or connected via an extension cable for tethered use (during use in the lab setting only). A connector box integrates sensor and motor data from the actuators on each limb and connects them to the battery and the analog to CANbus converter, which also accepts a trigger input to enable synchronization of exoskeleton data with outside systems, including motion capture and electromyography. The connector box also contains an emergency stop button that cuts power to the actuators when pressed. Additionally, the circuitry of each exoskeleton actuator contains a Bluetooth modem (Silicon Labs, San Jose, CA) that wirelessly connects to a control laptop running a custom, open source Python-based GUI (Tucker, Chen, Hammel, Damiano, & Bulea, 2020). The GUI allows an operator (an engineer or physical thereapist) to set and update the exoskeleton configuration parameters and thresholds for the finite state controller and the applied torques, calibrate the angles and FSR sensors, stream and display data for controller tuning, enable and disable torque application, and save data (Tucker, Chen, Hammel, Damiano, & Bulea, 2020).

The operational settings of the P.REX/Agilik control system will be established during the in lab use portion of the study, following the same procedures used in our current study (#13-CC-0210). A key setting is the preset maximum torque magnitude, which imposes a limit on the amount of torque (force) that can be applied by the exoskeleton to the user’s legs. The embedded software of the exoskeleton will monitor the torque applied to the limb to ensure the applied torque does not exceed this preset maximum. Similarly, the knee range of motion can be programmed into the software and the controller will automatically stop the robotic actuator from providing torque if/when knee angle is measured to be outside this range. These settings, and all other parameters of the device, are configured by an NIH operator (engineer or physical therapist) during the in-lab use portion of the study to ensure the device can be used safely during overground walking at various speeds. When participants use the device in the community, they will be able to turn the exoskeleton on and off but they will not be able to adjust these settings. If adjustments are required, they will need to contact the study staff to perform them. Finally, the exoskeleton is equipped with an emergency switch that is easily reached by the participant, their caretaker, or a physical therapist; pressing this switch cuts the system power. It can be pressed at any time if the device is operating abnormally or if the participant experiences discomfort. When participants are operating the device in the community, they will have access to this emergency switch at all times with the ability to cut the system power as needed. They will be trained on independent usage of this emergency switch during the accommodation/in-lab training period.

6.1.2 Dosing and Administration

After provision of informed consent and assent and assessment of eligibility, all participants enrolled in the study protocol will be randomized between Group A and

Group B. This is a crossover study. Group A will receive the study intervention first while Group B members receive the control first. After completion of the 12-week standard therapy period, cross over will occur for Group B participants, and they will begin another 12-week block consisting of in-lab training with the intervention. Following completion of the in-lab training, Group B participants will begin the last 12-week block consisting of community use of the device, or skip the community use block resulting in a return to standard therapy and an earlier study completion. Determination of whether to participate in the community use portion will be made by the patient, their caregiver and the study team.

For Group A participants, after 12 weeks of in-lab training, they will either enter into the 12-week community-use portion of the protocol or skip the community use block resulting in a return to standard therapy and an earlier study completion. Determination of which training will occur for Group A at this point is determined by the patient, their caregiver and the study team. Following completion of the 12-week community use portion for those who chose to participate, Group A will cross-over to participate in the control arm, consisting of 12 weeks of standard therapy without the device. This design allows all participants to have the same dosage of intervention but varies the order they are received between groups.

The dosing of the intervention device will vary slightly between blocks of the protocol. During the accommodation/in-lab training sessions held in the lab at NIH, patients are will complete visits lasting up to 4 hours in which the exoskeleton device will used under the direct supervision of the physical therapist. The primary purpose of this phase is to practice using the device in preparation for taking the device outside the clinical setting without supervision by study team members. The dosage of these practice sessions is justified by our previous studies which have found up to 4-hour sessions are appropriate for pediatric patients.

During the community-based phase of the protocol, patients are prescribed a target of 1 hour per day for 5 days per week, with a maximum dosage of 4 hours of total usage on any given day. The purpose of this phase of the primary protocol is to evaluate the effectiveness of a robotic exoskeleton as a long-term rehabilitation strategy for pediatric patients with knee extension deficiency. The higher training dose in this study is supported by previous long-term rehabilitation strategies that warranted application to device-augmented strategies of similar high dosages (Bleyenheuft, Arnould, Brandao, Bleyenheuft, & Gordon, 2015).

6.1.3 Dose Escalation

This is not applicable because this protocol is for a device-based intervention with a specified minimum and maximum dosage that will not change over the course of the study.

6.1.4 Dose Limiting Toxicity

Not applicable.

6.1.5 Dose Modifications

Not applicable.

6.1.6 Device Administration

During the 10 accommodation/in-lab training sessions each patient will participate in before using the device independently outside of the clinical setting, the patient will be instructed on how to safely don/doff the exoskeleton as well as operate the device using the GUI interface.

6.2 Preparation/Handling/Storage/Accountability

Storage of the P.REX/Agilik device will be handled by study team members for all visits preceding the community intervention. The device will be completely assembled by the time the participant encounters it for use. When participants take the device home, they will have been properly counseled on storage and handling. The participant will be advised to store the device at room temperature (between 10-40^{oC}) in a safe location within their home. The device should only be used by the participant and should only be handled by the participant and/or a trained caregiver. The device has a battery that is required to be charged prior to use. To ensure the device is ready to use each day, the participant will be advised to charge the device every evening at completion of that day's use. They will be informed to be sure to turn the battery off by pressing and holding the button upon completion of walking practice and before travelling with the device. The manufacturers pamphlet detailing device components and instructions on how to operate and handle them will also be provided to the participant.

6.2.1 Acquisition and Accountability

The intervention device is composed of multiple parts. The actuator, aluminum upright and associated parts will come from the manufacturer, Bionic Power. The custom knee-foot-ankle orthotic will be digitally casted in our facility during a visit. The finished orthotic will be shipped from Ultraflex Systems to be assembled with the actuator to form the robotic exoskeleton by the NIH study team. The force sensitive resistor (FSR, Interlink Electronics, Westlake Village, CA) will be shipped from Interlink Electronics to be included in the exoskeleton. The NIH study team will assemble the complete device once all components have been received. For a more detail description of the robotic exoskeleton system, please refer to section 6.1.1 on the study intervention device description

6.2.2 Formulation, Appearance, Packaging, and Labeling

Please see the image to the right for a visualization of the product appearance.

The metal uprights, actuator and associated embedded electronics will come from Bionic Power. The customized thermoplastic components will be coming from Ultraflex. Our study team members will assemble the two, along with FSRs from Interlink Electronics, at our NIH facility.

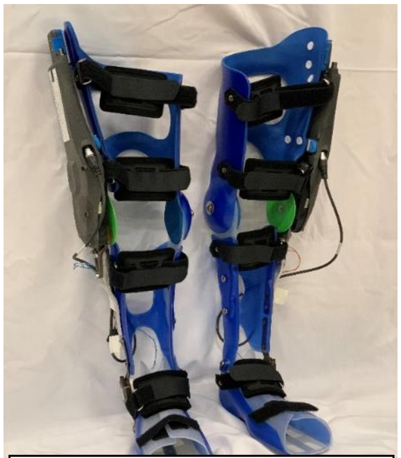

Figure 2. P.REX/Agilik Device

6.2.3 Product Storage and Stability

The device must be stored between 10-40 degrees Celsius. The device should not be used near any source of pure oxygen.

6.2.4 Preparation

All preparation of the device will be completed by study staff, and the device will not need to be disassembled at any point for storage, unless readjusting or part replacement needs to occur. To assemble the exoskeleton, the robotic exoskeleton (composed of the actuator and aluminum upright) needs to be aligned with the lateral femoral epicondyle (LFE) so that the central pivot point of the robot is adjacent to the LFE. The knee-ankle-foot orthosis (KAFO) shells should be constructed to match the lower-limb and coordinated with the robotic exoskeleton to ensure the surfaces match when attaching each actuator at the KAFO thigh shell. Upper and lower spacers will be provided by the Agilik manufacturers to allow for personalization of the device to the lower limb of each participant. Upon placement of the spacers, they can be grinded to match the surface of the KAFO shells. Study staff may also trim shells so they do not cover parts of the Agilik actuators or irritate the participant when worn. The spacers should be adhered to the actuator and KAFO shells by drilling, being sure not to screw bolts into the inserts past 7 mm. The next part of the assembly involves the aluminum upright, which attaches to the drive arm of the actuator by bolts, which need to be tightened to 4 Nm. This aluminum upright can be bent to match the lower shell of the KAFO.

The foot sensor and cable will be installed by the study staff at NIH. The foot sensor cable can be run through the plastic cable clips on the aluminum upright and the drive arm. The cable should be connected to the actuator by the connector box. See the image above for the complete set up of the device with cables.

6.3 Measures to Minimize Bias: Randomization and Blinding

After visit 0 where eligibility is determined, participants will be randomized within the cohort to either start as the study intervention group, deemed Group A or to start as the control group, deemed Group B.

To minimize bias in the baseline, interim, and post-treatment analyses, spatiotemporal gait parameters and gait kinematics will be analyzed by laboratory researchers blinded to the participants' exoskeleton mode of operation.

6.4 Study Intervention Compliance

Adherence to this protocol, including use of the intervention device during the community-based portion of the study design, will be assessed by study team members at conclusion of the study to attend to our safety and feasibility tertiary objective listed in Table 3. Throughout the protocol, study staff will follow-up weekly with participants to document their compliance with the study procedures and dosages. The device itself saves usage data and will allow us to track compliance in terms of total hours of use. The data saved locally on the embedded electronics does not include any personal identifiable information (PII).

6.5 Concomitant Therapy

For this protocol, a prescription medication is defined as a medication that can be prescribed only by a properly authorized/licensed clinician. Medications to be reported in the Case Report Form

(CRF) are concomitant prescription medications, over-the-counter medications and supplements. There is no anticipated effect on the outcome of participants continuing the use of previously established medications. The control for this study will be standard therapy for neurological disorders resulting in crouch gait or knee extension deficiency. We aim to examine the effect of our treatment on long-term retention of improvements in gait mechanics as compared to the same duration of standard therapy.

7 STUDY INTERVENTION DISCONTINUATION AND PARTICIPANT DISCONTINUATION/WITHDRAWAL

7.1 Discontinuation of Study Intervention

Discontinuation from the exoskeleton intervention does not mean discontinuation from the study, and remaining study procedures should be completed as indicated by the study protocol, especially to meet safety and efficacy endpoints. If a clinically significant finding is identified after enrollment (including, but not limited to changes from baseline), the investigator or qualified designee will determine if any change in participant management is needed. Any new clinically relevant finding after discontinuation will be reported as an adverse event (AE).

The data to be collected at the time of study intervention discontinuation will include the following:

- Physical Knee Joint Assessment – to evaluate spasticity of the knee by a physical therapist using the modified Ashworth and Tardieu Scales
- Validated Clinical Scales of Function – PEDI-CAT, GMFM-66, GMCFS, 6-minute walk test, timed up and go
- Strength Testing – Biodex Isometric Testing
- Walking Task (baseline) – the participant will complete the walking task under their normal, daily walking conditions while motion capture and EMG data are collected.
- Skin Assessment – to evaluate for skin abrasions, pressure points or irritations from device use
- Quebec User Evaluation of Satisfaction with assistive Technology (QUEST 2.0) – if the patient participated in the community use portion of the protocol, they will complete a survey as a patient reported outcome measure of 12 satisfaction items (dimensions, weight, adjustments, safety, durability, ease of use, comfort, effectiveness, service delivery, repairs/servicing, professional service and follow-up services) at their Final Outcome Assessment visit, corresponding to visit 15 for Group A and visit 17 for Group B (See Appendix Document E for a copy of this satisfaction assessment)

Participants are free to withdraw from participation in the study at any time upon request.

An investigator may discontinue or withdraw a participant from the study intervention for the following reasons:

- Completion of study intervention
- Disease progression which requires discontinuation of the study intervention
- If any clinical adverse event (AE), laboratory abnormality, or other medical condition or situation occurs such that continued participation in the study would not be in the best interest of the participant

- Investigator discretion
- Positive pregnancy test

A participant is unable to re-enroll in the exoskeleton intervention study for 1 year.

Participants will not be given any written report at the completion of this study. Research assessments will be shared with participants if relevant to their health and well-being. All procedures and evaluation in this study protocol are for research purposes, and there is no anticipated direct benefit from participation in the study. The history and physical is done to ensure that prospective study participants meet the inclusion criteria.

7.2 Participant Discontinuation/Withdrawal from the Study

Participants are free to withdraw from participation in the study at any time upon request. Prior to removal from study, effort must be made to have all subjects complete a final evaluation visit within approximately 3 weeks of the last robotic exoskeleton training session.

An investigator may discontinue or withdraw a participant from the study for the following reasons:

- Significant study intervention non-compliance
- If the participant meets an exclusion criterion (either newly developed or not previously recognized) that precludes further study participation
- Subject has completed the study follow-up period
- Death
- Screen Failure

The reason for participant discontinuation or withdrawal from the study will be documented in the patient's medical record. Subjects who sign the informed consent/assent form and are randomized but do not receive the study intervention may be replaced. Subjects who sign the informed consent/assent form, and are randomized and receive the study intervention, and subsequently withdraw, or are withdrawn or discontinued from the study, will not be replaced and will not count towards the overall accrual numbers.

7.3 Lost to Follow-up

A participant will be considered lost to follow-up if he or she fails to be contacted for 4 consecutive weeks. To mitigate the potential for lost to follow-up, our study staff will contact participants on a weekly basis.

The following actions must be taken if a participant fails to return to the clinic for a required study visit:

- The site will attempt to contact the participant and reschedule the missed visit within 1 week and counsel the participant on the importance of maintaining the assigned visit schedule and ascertain if the participant wishes to and/or should continue in the study.
- Before a participant is deemed lost to follow-up, the investigator or designee will make every effort to regain contact with the participant (including where possible, 3 telephone calls and, if necessary, a certified letter to the participant's last known mailing address or local equivalent). These contact attempts will be documented in the participant's medical record or study file.

- Should the participant continue to be unreachable, he or she will be considered to have withdrawn from the study with a primary reason of lost to follow-up.

8 STUDY ASSESSMENTS AND PROCEDURES

8.1 Screening Procedures

8.1.1 Screening activities performed prior to obtaining informed consent/assent

Minimal risk activities that may be performed before the subject has signed a consent/assent include the following:

- Email, written, in person or telephone communications with prospective subjects to review inclusion and exclusion criteria that can be assessed without manipulating the patient's limbs (such as age, gait pathology diagnosis, ability to walk 10 feet unassisted, history of seizures or other health condition affecting walking ability).
- Review of existing medical records to include H&P, laboratory studies, or therapy notes that may indicate walking function, knee and ankle range of motion, foot-thigh angle and hamstring contracture
- Review of existing MRI, x-ray, or CT images.
- Review of existing photographs or videos.

8.1.2 Screening activities performed after a consent/assent for screening has been signed

The following activities will be performed only after the subject has signed the separate screening consent and assent for this study, as described in Section 10.1.1. Assessments performed at outside facilities or on another NIH protocol within the timeframes below may also be used to determine eligibility once a participant has signed the screening consent and assent.

All screening tests and procedures must be performed within 28 days prior to enrollment. All screening activities listed below are of minimal risk to the patient and will be performed at visit 0 to determine eligibility for the study:

- Knee joint range of motion (at least 25° in sagittal plane to meet eligibility)
- Straight leg raising test to assess hamstring contracture (does not limit ability to participate)
- Ankle joint range of motion (at least 15° in sagittal plane to meet eligibility)
- Measure of foot-thigh angle (-15 to 30° in prone position to meet eligibility)
- Walk test without stopping or use of walking aid (must be able to walk at least 10 feet to meet eligibility)
- History and physical evaluation by licensed RMD physician in the absence of existing medical records, to include medication review and diagnosis of CP, MD, SB or iSCI

8.2 Study Evaluations & Procedures

Visit 0 – Initial Evaluation, History and Physical

Section 8.1.1 and section 8.1.2 detail the evaluation procedures to occur at visit 0 to determine eligibility of the patient for this protocol, which includes provision of separate screening consent and assent forms. Once eligibility is determined and the participant is enrolled in the study, they must review and sign the standard informed consent and assent forms for the study under supervision of the PI and a clinical team member, as described in Section 10.1.1. After provision of informed consent/assent, the participant will be enrolled into one of the two subject pools by diagnosis. After subject pool assignment at visit 0, that participant will remain with their assigned subject pool for the rest of the study and all data analysis will occur within subject pool. Randomization into Group A or Group B will occur separately within subject pools.

Visit 1 – Group A and Group B

Following inclusion, randomization, and provision of a signed standard consent/assent, an initial assessment will take place at the motion analysis laboratory in the NIH Clinical Center for all participants. This visit will include a baseline assessment consisting of the following procedures and analyses:

1. *Vital Signs*
   The following measures will be collected at the start of every visit by a licensed RMD physician or physical therapist; heart rate, blood pressure, pulse oximeter, breathes/minute and temperature. At this initial visit, height and weight will also be obtained using the meter stick and scale respectively.
2. *Physical Knee Joint Assessment, Validated Clinical Scales of Function, and Strength Testing*
   A physical therapist will evaluate passive range of motion, strength and spasticity on both knees of the participant. Spasticity will be measured by the modified Ashworth scale and the Tardieu scale. The participant will also undergo the 6-minute walk test and the Timed Up and Go to evaluate motor control. To classify the participants gross motor function, the PEDI-CAT will be completed by the participant or parent and the physical therapist will determine GMFM-66 and GMFCS. Knee flexion and extension strength will be measured during an isometric test on a Biodex dynamometer
3. *Motion Capture*
   Prior to gait assessment, passive reflective markers will be taped on the skin on each segment of the lower limb. These markers will be tracked using a VICON motion capture system (VICON, Lake Forest, CA), which also records a digital video of the participant standing or moving in 3-dimensional space. In-ground force plates (AMTI, Watertown, MA) will measure the ground reaction forces/joint moments while participants walk at self-selected and fast speeds along the straight 10m long distance. If they are observed to be unstable, participants will wear a harness (ZeroG®, Aretech LLC, Ashburn, VA) during the motion capture and gait assessment. The ZeroG® system monitors participants while they walk to protect them from contacting the ground in the event of a fall by supporting their body weight if they become unstable.
4. *EMG*
   As part of the gait assessment, surface EMG electrodes will be used to measure muscle activity during walking. Adhesive surface electrodes (Delsys Inc., Boston, MA) will be placed on the skin over the muscle bellies following skin preparation with an alcohol pad. Electrodes will be placed bilaterally on knee extensor muscles, knee flexor muscles,

ankle dorsiflexor muscles, and ankle plantar flexor muscles by a trained therapist. EMG electrode pads placed directly on the skin will not interfere with the brace. During trials when FES is used, surface EMG amplifiers will be blanked during periods of stimulation to prevent amplifier saturation. FES is an optional addition to this protocol. The decision to use FES will be up to the physician and the patient participating in the study.

5. *Baseline Walking Task*
   After equipment setup and subject preparation is complete, data collection will begin. Participants will be asked to complete a walking task for gait assessment. During the initial assessment at visit 1, the walking task will be completed in bare feet for a full biomechanical analysis. All walking tasks in subsequent visits – including baseline walking condition with the device – and associated analyses and comparisons will be completed while wearing shoes. Walking can occur on a treadmill or overground, and may occur within the motion capture laboratory or within the corridor of the Rehabilitation Medicine Department. For overground, the participant will walk from one end of the motion capture area to the other, repetitively, at their self-selected pace. For the treadmill, the participant will walk on a split belt treadmill (Bertec, Columbus, OH) at their self-selected pace. They will be instructed to walk continuously but if they report fatigue or are unable to complete a step the treadmill will be stopped. Participants may wear a harness (ZeroG®, Aretech LLC, Ashburn, VA) for safety during the walking task. Note that a walking aid that is routinely used in daily activities of locomotion (e.g. a walker, crutches, or ankle-foot orthosis) can be used during the walking task. The corridor of the Rehabilitation Medicine Department can be used for longer continuous walking trials as needed. The participant will be shadowed by a trained physical therapist and closely monitored by study staff during all walking trials.
6. *Gait Analysis*
   Following the conclusion of the visit, spatiotemporal gait parameters and gait kinematics will be analyzed by laboratory researchers blinded to the participants' exoskeleton mode of operation.
7. *Orthosis Digital Casting*
   This will occur at visit 1 for Group A and as a separate visit 2 solely for the purpose of orthosis digital casting for Group B. After the gait assessment, the principal investigator will perform digital casting of each lower limb for creation of the orthotic shells. One at a time, the participant's limb will be placed in a clear plastic stand to position the limb for a 360° image from the upper thigh to the base of the foot collected by an iPad operating the digital casting app. If digital costing is not feasible, traditional casting may be substituted. Casting will be performed at the NIH clinical center by a certified orthotist/prosthetist/ped-orthotists. A set of braces for both legs will be made for the P.REX/Agilik from these casts. If the participant walks with a locked ankle-foot orthosis under normal conditions, the ankle of the custom orthosis may also be locked.
8. *Photo/Video Recording (optional)*
   The motion capture software takes a digital video of the participant while recording gait analysis, but as standard procedure, digital photos and videos on a study team members iPhone or camera may be taken of the participant wearing and/or walking in the device during any visit to the NIH for research purposes only. The participant may choose to sign the Authorization for Recording, Filming, and/or Photographing of Patients in the Clinical Center (Appendix Document F), which would give permission to use any

photographs or videos obtained in conjugation with motion analysis of gait for publications and public presentations of this study. If the participant decides to sign the authorization, they can necessitate obscuring of identifying features (i.e. face) based on their preference. If they do not sign the authorization, any photos or videos taken of them will be used internally for research purposes only and will not be used in any public presentation or publication of this study.

Exoskeleton Set-up and Calibration – Visit 2 for Group A and Visit 4 for Group B

Prior to this visit, customized thermoplastic leg braces for the P.REX/Agilik will be fabricated for each subject. The participant and parent/guardian will be notified to bring their own shoes suitable for the device (i.e. tennis or comfortable walking shoe that can accommodate the slightly increased width and length of the thermoplastic inside of the shoe). The user-specific P.REX/Agilik will be created by assembling the custom braces with an adjustable ankle joint (free or locked) and the Agilik® knee actuator.

This visit will begin with examination of the custom fabricated leg braces to assure proper fit. Next, the control settings of the P.REX/Agilik including the level and timing of the robotic assistance will be calibrated and tuned. Settings will be incrementally adjusted as the participant walks with the P.REX/Agilik in the respective conditions (Table 4).

1. *Vital Signs*
   Heart rate, blood pressure, pulse oximeter, breaths/minute, and temperature.
2. *Orthosis Examination*
   The biomechanical fit and alignment of the custom P.REX/Agilik will be examined by the principal investigator, physical therapist and/or the medically responsible investigator (MRI) when the subject first dons the device during this exoskeleton set-up visit. In the event the fit of the orthosis is not acceptable the session will be cancelled and the limb will be re-measured using the digital casting procedure to allow for modifying and recasting the orthosis to correct the fit. At the beginning of each subsequent visit during which the participant will wear the custom P.REX/Agilik the fit of the orthosis will be visually and physically inspected for safety to assure minimal risk for skin problems.
3. *Calibration and Tuning of P.REX/Agilik*
   All participants will start with initial settings based on the ankle and knee positions at mid-stance determined by the initial gait analysis at visit 1. The amount of assistance or resistance can be varied (versus all-or-none) depending on the individual patient's abilities. This is why "tuning" is needed. The goal is to allow patients to use whatever muscle activity they have that helps them be more upright, reduce activity that makes them more crouched, or provide support when they cannot activate their muscles enough on their own. Typically, in assistive modes, the tuning strategy is to achieve a neutral position at the ankle (0° of both dorsiflexion and plantarflexion), then to focus on increasing knee extension as much as possible. Once the maximal extension is achieved, the amount of assistance will be incrementally decreased to the lowest level possible while still maintaining the same amount of knee extension. These incremental adjustments are based on visual inspection of the knee angle during gait by the study staff. The amount of resistance is similarly tuned; the timing (during stance or late-swing) and level of resistance during walking will be incrementally increased until the baseline knee extension angle during stance and swing phase can no longer be reached during walking (resistance value 1). The resistance will be increased further until walking is no

longer feasible (resistance value 2), at which point the final resistance amount will be lowered to approximately the midway point between these two values.

4. *Functional Electrical Stimulation (FES) – optional*
   This procedure is optional and subject participation will be determined by the licensed RMD physician present at the trial and subject agreement. The surface FES system for knee extension assist will be calibrated for each subject. The calibration may either be performed during standing and walking, or alternatively while the participant is supine on a mat with his/her legs placed on a wedge. FES electrodes will be placed over the belly of the quadriceps muscles. Starting from 30 Hz frequency, 50 microseconds pulse width, and current amplitude at the threshold that the participant first detects the stimulation (as indicated by a mild tapping sensation in the muscle), we will systematically increase amplitude of FES in slow increments to participant tolerance. If the stimulation is uncomfortable at any increment, we will decrease it to the previous level before the participant felt discomfort. If the contraction produced is not smooth (muscles twitches are not close enough to summate), we will increase the frequency by 5 Hz until it is smooth, up to a maximum of 45 Hz. This may or may not necessitate a concomitant decrease in amplitude, depending on participant tolerance. Our priority is to keep the pulse width at a low level to maximize comfort, but slight adjustments (down to 35 µsec or up to 200 µsec) to obtain a more effective or more tolerable contraction in some individuals may be needed. The subjects may also be asked to extend their knee while the stimulation is on. We will observe how the FES affects knee extension. Even if the maximum tolerable FES does not increase knee extension, configurations with FES will proceed at those stimulation levels. Many accommodate to stimulation after a few sessions, so it is possible that the intensity could be increased over additional visits as tolerance improves. Location of FES electrodes on the quadriceps will be recorded for consistent placement across sessions and will be placed over the middle (largest portion) of the muscle belly. If the location interferes with surface EMG electrodes on the knee extensors, EMG electrodes will be moved to an adjacent location as identified by an experienced therapist.
5. *Initial Walking Practice*
   Walking practice will be performed overground or optionally on the treadmill. This activity will mirror the walking task described at baseline, but will be performed while wearing the exoskeleton. During this practice, the initial operational mode(s) of the exoskeleton will be identified (Table 4), with a priority on identifying a resistive mode as the primary and then an interleaved and/or assistive mode(s) as secondary. In addition, the participant will have the option to walk continuously over longer distances by utilizing the hallways of the Rehabilitation Medicine Department at NIH. This could better reflect the potential environment for community use. Study team members can elect for the participant to complete walking practice in this manner if it would be beneficial for the patient and the patient agrees. The participant will be shadowed by a trained physical therapist and closely monitored by study staff during all walking trials.

Accommodation/In-Lab Training– Visit 3-12 for Group A and Visit 5-14 for Group B

For up to 10 visits in the 5 weeks following the exoskeleton calibration, patients will return to the NIH motion capture lab to participate in accommodation/in-lab training sessions to practice using the exoskeleton device. Each session will consist of exoskeleton walking practice with observation and instruction of exoskeleton operation by our study team.

1. *Vital Signs*
   Will be performed as detailed in Exoskeleton Set-Up and Calibration.
2. *Orthosis Examination*
   Will be performed as detailed in Exoskeleton Set-Up and Calibration.
3. *Functional Electrical Stimulation (FES) – optional*
   Will be performed as detailed in Exoskeleton Set-Up and Calibration.
4. *Walking Practice and Instruction*
   Will be performed as detailed in Exoskeleton Set-Up and Calibration - Initial Walking Practice. The focus will be on practicing with the primary mode first, followed by any secondary modes as applicable.

Outcome Assessment – Visit 13-17 for Group A and Visit 3, 15-18 for Group B

Each outcome assessment will consist of the same procedures listed below.

1. *Vital Signs*
   Will be performed as detailed in Exoskeleton Set-Up and Calibration.
2. *Physical Knee Joint Assessment, Validated Clinical Scales of Function, and Strength Testing*
   Will be performed as detailed in Visit 1. Procedures and analyses include the Modified Ashworth and Tardieu Scales to measure spasticity, clinical scales (PEDI-CAT, GMFM-66, GMCFS, 6-minute walk test, timed up and go) to measure motor control and function, and Biodex isometric testing to measure muscle strength. The GMFM-66 will only be performed at the Baseline, Initial and Final Outcome Assessments. All of these measures will be optionally performed at the Intermediate Outcome Assessment at the discretion of the study staff.
3. *Orthosis Examination*
   Will be performed as detailed in Exoskeleton Set-Up and Calibration.
4. *Motion Capture*
   Will be performed as detailed in Visit 1.
5. *EMG*
   Will be performed as detailed in Visit 1. Only muscles necessary for muscle control of the active brace will be instrumented.
6. *Functional Electrical Stimulation (FES) – optional*
   Will be performed as detailed in Exoskeleton Set-Up and Calibration.
7. *Walking Task*
   Will be performed as detailed in Exoskeleton Set-Up and Calibration - Initial Walking Practice.
8. *Gait Analysis*
   Will be performed as detailed in Visit 1.
9. *Photo/Video Recording (optional)*
   Will be performed as detailed in Visit 1.
10. *Patient Experience Assessment*
    At the patient’s final visit, likely the Follow-Up Outcome Assessment if the protocol is completed in full, any patient who participated in the community use portion of the protocol will be asked to complete the QUEST 2.0 as a self-reported outcome measure assessment on 12 items related to device use and function (dimension, weight, adjustment, safety, durability, ease of use, comfort, effectiveness, service delivery, repairs/servicing, professional service, follow-up service). The survey will be completed

on paper at the patient's Final Outcome Assessment to conclude exoskeleton use. A copy of this survey is included as a PDF in Appendix Document E. Previous use of this assessment found it was a valuable tool for assessing satisfaction of assistive technology use (Demers, Weiss-Lambrou, & Ska, 2002).

8.2.1 Biospecimen Evaluations

Not applicable.

8.2.2 Correlative Studies for Research/Pharmacokinetic Studies

Not applicable.

8.2.3 Samples for Genetic/Genomic Analysis

8.2.3.1 Description of the scope of genetic/genomic analysis

Not applicable.

8.2.3.2 Description of how privacy and confidentiality of medical information will be maximized

Not applicable.

8.2.3.3 Management of Results

Not applicable.

8.2.3.4 Genetic counseling

Not applicable.

8.3 Safety and Other Assessments

Subjects will be monitored during all study procedures for any adverse effects during the experiment by the PI, an associate investigator or a medically responsible individual. If adverse effects are noted or the subject is not comfortable during any study, experiments will be stopped.

The following procedures/evaluations are planned as part of the protocol:

- **Physical examination**: A complete physical examination will be performed by a licensed RMD physician at visit 0 for the sole purpose of determining eligibility in the study. This examination will include height and weight, organ systems, motor or vision assessment, musculoskeletal and neurological assessments and other functional abilities discussed in section 8.1.2. Each patient must meet the parameters outlined in the inclusion and exclusion criteria for this study in order to participate.
- **Vital signs**: At the start of every visit to the Neurorehabilitation and Biomechanics Lab at NIH, vital signs will be taken. These measures will include heart rate, blood pressure, pulse oximeter, breathes/minute, and temperature. All values will need to be within normal range for the patient to complete the visit due to the physical activity requirements of each visit.
- **Counseling procedures on activity considerations**: Participants involved in the community intervention portion of the protocol are recommended to walk in the exoskeleton device for at least 1 hour per day, 5 days per week for 12 weeks to meet the

minimum dosage required of this protocol. There are no other counseling procedures including dietary restrictions or activity considerations outside of this requirement.

- **Assessment of study intervention adherence**: Please refer to Study Intervention Compliance, section **6.4**.
- **Administration of questionnaires or other instruments**: At the completion of the study, each participant will complete the QUEST 2.0 regarding their use of assistive technology in this study. One purpose of this document is to review each participant's comments on the safety and overall experience of using the wearable robot in the community setting while being involved in this research study. See Appendix Document E for a copy of this assessment. If non-English speakers are enrolled, assessments related to the primary objectives will be validated in their languages.
- **Assessment of adverse events**: Please refer to section 8.4.4 for a detailed description of assessment and follow-up of adverse events.

Participants will not be given any written report at the completions of this study. Research assessments will be shared with participants if relevant to their health and well-being. All procedures and evaluations in this study protocol are for research purposes, and there is no anticipated direct benefit from participation in the study. The history and physical is done to ensure that prospective study participants meet the inclusion criteria.

8.4 Adverse Events and Serious Adverse Events

8.4.1 Definition of Adverse Event

Adverse event means any untoward medical occurrence associated with the use of an intervention in humans, whether or not considered intervention-related (21 CFR 312.32 (a)).

8.4.2 Definition of Serious Adverse Events (SAE)

An adverse event (AE) or suspected adverse reaction is considered "serious" if, in the view of either the investigator or sponsor, it results in any of the following outcomes: death, a life-threatening adverse event, inpatient hospitalization or prolongation of existing hospitalization, a persistent or significant incapacity or substantial disruption of the ability to conduct normal life functions, or a congenital anomaly/birth defect. Important medical events that may not result in death, be life-threatening, or require hospitalization may be considered serious when, based upon appropriate medical judgment, they may jeopardize the participant and may require medical or surgical intervention to prevent one of the outcomes listed in this definition. An example of such medical event includes seizures that do not result in inpatient hospitalization.

8.4.3 Classification of an Adverse Event

8.4.3.1 Severity of Event

All AEs will be assessed by the study clinician using a protocol defined grading system.

For adverse events (AEs) not included in the protocol defined grading system, the following guidelines will be used to describe severity.

- **Mild** – Events require minimal or no treatment and do not interfere with the participant's daily activities.
- **Moderate** – Events result in a low level of inconvenience or concern with the therapeutic measures. Moderate events may cause some interference with functioning.
- **Severe** – Events interrupt a participant's usual daily activity and may require systemic drug therapy or other treatment. Severe events are usually potentially life-threatening or incapacitating. Of note, the term "severe" does not necessarily equate to "serious".

8.4.3.2 Relationship to Study Intervention

All adverse events (AEs) must have their relationship to study intervention assessed by the investigator who examines and evaluates the participant based on temporal relationship and his/her clinical judgment. The degree of certainty about causality will be graded using the categories below. In a clinical trial, the study product must always be suspect.

- **Related** – The AE is known to occur with the study intervention, there is a reasonable possibility that the study intervention caused the AE, or there is a temporal relationship between the study intervention and event. Reasonable possibility means that there is evidence to suggest a causal relationship between the study intervention and the AE.
- **Not Related** – There is not a reasonable possibility that the administration of the study intervention caused the event, there is no temporal relationship between the study intervention and event onset, or an alternate etiology has been established.

8.4.3.3 Expectedness

The principal investigator (PI) will be responsible for determining whether an adverse event (AE) is expected or unexpected. An AE will be considered unexpected if the nature, severity, or frequency of the event is not consistent with the risk information previously described for the study intervention.

8.4.4 Time Period and Frequency for Event Assessment and Follow-Up

The occurrence of an adverse event (AE) or serious adverse event (SAE) may come to the attention of study personnel during study visits and interviews of a study participant presenting for medical care, or upon review by a study monitor.

All AEs including local and systemic reactions not meeting the criteria for SAEs will be captured on the appropriate case report form (CRF). Information to be collected includes event description, time of onset, clinician's assessment of severity, relationship to study product (assessed only by those with the training and authority to make a diagnosis), and time of resolution/stabilization of the event. All AEs occurring while on study must be documented appropriately regardless of relationship. All AEs will be followed to adequate resolution.

Any medical condition that is present at the time that the participant is screened will be considered as baseline and not reported as an AE. However, if the study participant's condition deteriorates at any time during the study, it will be recorded as an AE.

Changes in the severity of an AE will be documented to allow an assessment of the duration of the event at each level of severity to be performed. AEs characterized as intermittent require documentation of onset and duration of each episode.

The principal investigator (PI) will record all reportable events with start dates occurring any time after informed consent/assent is obtained until 7 (for non-serious AEs) or 30 days (for SAEs) after the last day of study participation. At each study visit, the investigator will inquire about the occurrence of AE/SAEs since the last visit. A vital sign assessment will be performed at the start of every visit to monitor the individual for any change from baseline measures taken at the first visit that may indicate an AE is of concern. At the start of each visit to the NIH, the participant will be asked to provide any updates since their previous encounter to include falls, injuries, or other health events. Communication will occur weekly with the participant via phone or email during the community use or standard therapy portions of the protocol. An AE review will occur at least every 2 weeks during the weeks of community use/standard therapy to ensure the patient is monitored for the occurrence of an AE/SAE while outside of the clinical setting. Events will be followed for outcome information until resolution or stabilization.

8.4.5 Adverse Event Reporting

Reportable events will be tracked and submitted to the IRB as outlined in Policy 801.

8.4.6 Serious Adverse Event Reporting

The study investigator shall complete an Unanticipated Adverse Device Effect Form and submit to the study sponsor as soon as possible, but in no event later than 7 calendar days after the investigator first learns of the effect. The study sponsor is responsible for conducting an evaluation of an unanticipated adverse device effect and shall report the results of such evaluation to the Food and Drug Administration (FDA) and to all reviewing IRBs and participating investigators within 10 working days after the sponsor first receives notice of the effect. Thereafter, the sponsor shall submit such additional reports concerning the effect as FDA requests.

In consultation with the PI, a trained member of the study team will be responsible for conducting an evaluation of all adverse events and shall report the results of such evaluation to the NIH Institutional Review Board (IRB) as per Policy 801.

8.4.7 NIH Intramural IRB Reporting of IND Safety Reports

Only IND Safety Reports that meet the definition of an unanticipated problem or is new information that might affect the willingness of subjects on the NIH study to enroll or remain in the study will need to be reported to the NIH Intramural IRB.

8.4.8 Events of Special Interest

Not applicable

8.4.9 Reporting of Pregnancy

Not applicable.

8.5 Unanticipated Problems

8.5.1 Definition of Unanticipated Problems (UP)

Any incident, experience, or outcome that meets **all** of the following criteria:

- Unexpected in terms of nature, severity, or frequency given (a) the research procedures that are described in the protocol-related documents, such as the Institutional Review Board (IRB)-approved research protocol and informed consent and assent documents; and (b) the characteristics of the participant population being studied; and
- Related or possibly related to participation in the research ("possibly related" means there is a reasonable possibility that the incident, experience, or outcome may have been caused by the procedures involved in the research); and
- Suggests that the research places participants or others (which many include research staff, family members or other individuals not directly participating in the research) at a greater risk of harm (including physical, psychological, economic, or social harm) than was previously known or expected.

8.5.2 Unanticipated Problem Reporting

The investigator will report unanticipated problems (UPs) to the NIH Institutional Review Board (IRB) as per Policy 801.

9 STATISTICAL CONSIDERATIONS

9.1 Statistical Hypothesis

- *Primary Endpoint*:
  We will test the hypothesis that 12 weeks of community exoskeleton therapy will improve knee extension deficiency (i.e., crouch gait) during walking, as measured by peak knee extension during midstance. Peak knee extension during midstance is the primary outcome measure for this trial and thus was used to complete our power analysis for sample size required to achieve a significant change (See Sample Size Determination, Section 9.2 below). The outcome measure for the primary endpoint will be measured at the initial outcome assessment and following the 12-week community intervention (final outcome assessment).
- *Secondary Endpoints*:
  We will evaluate the hypothesis that 12 weeks of community exoskeleton therapy will improve peak and overall volitional knee extensor activation levels measured by peak activation during the stride and mean area under the curve, respectively. These outcome measures will be assessed at the initial and final outcome assessment visits, which correspond to immediately before and after the 12-week community use block.
  We will test the hypothesis that 12 weeks of community exoskeleton therapy will increase knee extensor muscle strength measured by maximum isometric knee extension torque on the Biodex dynamometer. This outcome measure will be assessed at the initial and final outcome assessments.

We will test the hypothesis that 12 weeks of community exoskeleton therapy will increase volitional gait speed (i.e., without the exoskeleton), assessed as the difference between the initial and final outcome assessments.
We will test the hypothesis that the effect of 12 weeks of community exoskeleton therapy on peak knee extension during midstance will persist after completion of the therapy. This hypothesis will be evaluated between measurements at the final outcome assessment and the follow up outcome assessment.
We will test the hypothesis that the order of standard therapy and exoskeleton intervention does not affect the primary outcome of peak knee angle during midstance. We will assess this hypothesis by comparing the difference in primary outcome measure at the initial and final assessments between Group A and Group B.

- *Tertiary/Exploratory Endpoints*:
  We will evaluate the hypothesis that change in peak knee extension is affected by dosage of the exoskeleton intervention by examining the peak knee extension at midstance at multiple time points, including: baseline, the initial assessment (after 6 weeks of in-lab use), the final assessment (after 12 weeks of community use), and at follow up (6 weeks after community use).
  We will test the hypothesis that knee extensor and flexor muscle spasticity will be reduced following 12 weeks of community exoskeleton therapy. The measure for this hypothesis will be the Modified Ashworth and Tardieu Scales and it will be evaluated at the initial and final outcome assessments.
  We will test the hypothesis that 12 weeks of community exoskeleton therapy will improve gross motor function. The measures for this hypothesis will be PEDI-CAT and GMFM-66 taken at the initial and final outcome assessments.
  We will test the hypothesis that community exoskeleton use is safe and feasible. The outcome measure for feasibility will be compliance with the study procedures and whether or not each participant met the study dosage instructions. The outcome measure for safety will be the occurrence of any adverse events. Each of these will be tracked through all study visits and procedures.

9.2 Sample Size Determination

The sample size is calculated based on the primary outcome of peak knee extension at midstance, which is a measure of sagittal plane knee angle during walking. Based on preliminary findings with the planned study intervention under our existing in-lab protocol (#13-CC-0210) we estimate the mean improvement of peak knee extension to be 3.0 ± 3 degrees (n = 3). With a significance level (alpha) of 0.05, 90% power, and lumped analysis between the two groups, a sample size of 13 participants completing the community exoskeleton intervention is required. Sample size was determined by power analysis for a paired t-test.

We therefore set our recruitment goal for two separate subject pools. We will aim for 13 participants with cerebral palsy, and 13 participants with knee extension deficiency from the weakness based neuromotor disorders (spina bifida, muscular dystrophy, and incomplete spinal cord injury). We expect 80% of participants to complete the study through the final outcome assessment. Additionally, we expect 25% of participants to complete the initial, in-lab phase of the study but elect not to complete the community use portion of the study. Therefore, 22

participants with cerebral palsy and 22 participants with other neuromotor disorders will be recruited.

We expect that 1-2 participants can be enrolled per month, and therefore the target enrollment can be achieved within 4-5 years, with an additional year for final completion of study procedures and follow up (i.e., total study duration of 6 years).

9.3 Populations for Analyses

A Per-Protocol analysis will be used wherein participants that completed at least 80% (9 weeks) of the study intervention in the community, as well as the Initial and Final Outcome Assessment visits, will be included in the data set for all outcome measures evaluated between these two assessments.

For outcome measures assessing persistence of intervention effects, a second Per-Protocol dataset will be established to include only those participants who completed at least 80% (9 weeks) of the study intervention in the community, as well as the Initial, Final and Follow-up Outcome Assessment visits.

For the exploratory analysis of safety and feasibility, the dataset will include all participants who have completed at least one visit with the exoskeleton intervention.

9.4 Statistical Analyses

9.4.1 General Approach

For descriptive statistics, continuous data will be presented as means with standard deviation. Categorical data will be presented as a range with percentages. The study will be powered under the assumption that no significant covariates are identified, and using the peak knee extension during midstance as the primary outcome and a paired t-test as the primary evaluation. The assumption for normality will be assessed as appropriate, and if normality is not found, we will use the signed rank test to evaluate. The same procedures will be utilized for analysis of secondary outcome measures evaluated between two time points, i.e., the Initial and Final Outcome Assessments and the Final and Follow-up outcome assessments. For exploratory analyses analyzing the dosage affect across all four outcome assessments, we will use repeated measures ANOVA. The effect of covariates will be analyzed using repeated measures ANCOVA.

9.4.2 Analysis of the Primary Endpoint

The primary endpoint is change in peak knee extension at midstance between two time points: Initial Outcome Assessment and Final Outcome Assessment. These time points correspond to immediately before and after the 12-week community use intervention period. We will quantify this endpoint as a longitudinal, repeated measure because it will evaluate the change in peak knee extension after 12 weeks of regular (5 days/week, 1 hour/day) use of the exoskeleton in the community. It will be reported descriptively as mean +/- standard deviation, with significant differences across time points described using p-values.

Assuming normality, the primary endpoint will be analyzed using a paired t-test to evaluate the difference in knee angle between the Initial and Final Outcome Assessments. The covariates of the primary endpoint include age, height, weight and mobility (defined

by functional scale GMFCS I-IV). Given the varying stages of development among children, the covariates identified here will account for physical limitations that may skew the results if unaccounted for and their effects will be evaluated using an ANCOVA modeling approach.

A Per-Protocol analysis will be used to assess the primary endpoint. This includes all participants who are enrolled in this study and have completed at least 80% (9 weeks) of the study intervention in the community. The subject pools will be analyzed separately. Within each subject pool, the two groups (Group A and Group B) will be analyzed together. All participants across both subject pools, who elect to participate in the community use, will receive the same dosage of intervention device, the only change being the order of the intervention and control arms between Groups A and B. The effect of the order between groups will be assessed within a secondary objective.

Only the participants who complete at least 9 weeks or 80% of the sessions between the designated time points for the primary objective will be included in the analysis. Owing to the relatively small sample size, we will not remove outliers. Those who are non-compliant or lost to follow up will be omitted from the analyses.

9.4.3 Analysis of the Secondary Endpoints

There are four secondary endpoints listed below. The first and second secondary endpoints are independent of the primary endpoint, while the third and fourth secondary endpoints are dependent on the primary endpoint.

(1) *Change in knee extensor muscle activation*

The first secondary endpoint is change in activation knee extensor muscles (vastii and rectus femoris). It will be assessed between two time points: Initial Outcome Assessment and Final Outcome Assessment which occur immediately before and after the 12-week community use block. This endpoint will be measured using peak and mean muscle activation. Muscle activation is determined using adhesive surface EMG electrodes placed on the skin over the belly of each muscle. For this outcome measure, we will assess the surface EMG data measured from knee extensors, including the vastii and rectus femoris. The peak knee extensor activation levels will be measured by peak activation during the stride and the mean knee extensor activation will be measured by area under the normalized EMG curve.

We will quantify this endpoint as a longitudinal, repeated measure because it will evaluate the change in peak/mean knee extensor muscle activation after 12 weeks of regular use of the exoskeleton in the community. It will be reported descriptively as mean +/- standard deviation, with significant differences across time points described using p-values.

Assuming normality and no significant covariates, this secondary endpoint will be analyzed using a paired t-test to evaluate difference in peak and mean knee extensor muscle activation between the Initial and Final Outcome Assessments. This secondary endpoint will fall under the Per-Protocol analysis as described in section 9.3 and discussed in section 9.4.2 for the primary endpoint as well.

(2) *Change in knee extensor muscle strength*

The second secondary endpoint is change in knee extensor muscle strength. It will be assessed between two time points: Initial Outcome Assessment and Final Outcome Assessment which occur immediately before and after the 12-week community use block. This endpoint will be measured during seated knee extension performed on a Biodex dynamometer. Knee extension strength will be quantified as the mean maximum extension torque exerted during 3 repetitions of isometric knee extension. It will be quantified at two knee angles: full knee extension (0°) and 30° of knee flexion. We will quantify this endpoint as a longitudinal, repeated measure because it will evaluate the change in knee extensor muscle strength after 12 weeks of regular (5 days/week, 1 hour/day) use of the exoskeleton in the community. It will be reported descriptively as mean +/- standard deviation, with significant differences across time points described using p-values.

Assuming normality and no significant covariates, this secondary endpoint will be analyzed using a paired t-test to evaluate difference in peak and mean knee extensor muscle activation between the Initial and Final Outcome Assessments. This secondary endpoint will fall under the Per-Protocol analysis as described in section 9.3 and discussed in section 9.4.2 for the primary endpoint as well.

(3) *Change in average gait speed*

The third secondary endpoint is change in average gait speed. It will be evaluated between two time points: Initial Outcome Assessment and Final Outcome Assessment which occur immediately before and after the 12-week community use block. Gait speed will be measured in meters per second. To calculate gait speed, the 6-minute walk test will be performed. This test allows for gait speed calculation using meters covered in 6 minutes. The motion capture system used during walking trials also has the capacity to record gait speed and will be used to in corroboration with the 6-minute walk test.

We will quantify this endpoint as a longitudinal, repeated measure because it will evaluate the change in average gait speed after 12 weeks of regular use of the exoskeleton in the community. It will be reported descriptively as mean +/- standard deviation, with significant differences across time points described using p-values.

Assuming normality and no significant covariates, this secondary endpoint will be analyzed using a paired t-test to evaluate difference in average gait speed between the Initial and Final Outcome Assessments. This secondary endpoint will fall under the Per-Protocol analysis as described in section 9.3 and discussed in section 9.4.2 for the primary endpoint as well.

(4) *Persistence of primary endpoint at Follow-up Assessment*

The fourth secondary endpoint will be an assessment of the primary endpoint, change in peak knee extension during midstance, between two different time points: final outcome assessment and follow-up outcome assessment. The objective will be evaluated at the follow-up visit to occur six-weeks after completion of the community use intervention period. We will quantify this endpoint as a longitudinal, repeated measure because it will evaluate for any change in peak knee extension between two time points, 6 weeks apart. It will be reported descriptively as mean +/- standard deviation, with significant differences across time points described using p-values.

If no significant change in peak knee extension is recorded between the specified time points above, then we will claim that persistence of effect has occurred.

Assuming normality and no significant covariates, this secondary endpoint will be analyzed using a paired t-test to evaluate for any difference in peak knee extension during midstance between the Final and Follow-up Outcome Assessments.

A second Per-Protocol dataset will be used for this secondary endpoint. It will include only those participants who completed at least 80% (9 weeks) of the study intervention in the community, as well as the Initial, Final and Follow-up Outcome Assessment visits.

(5) *Effect of order of exoskeleton intervention and control arms on primary endpoint*

To control for time and order differences within subject pools but between groups A and B, the primary endpoint (peak knee extension at midstance) will be assessed separately within Group A and Group B between two time points: Initial Outcome Assessment and Final Outcome Assessment which occur immediately before and after the 12-week community use block. This objective aims to evaluate the primary endpoint while controlling for the difference of order between groups. Group A will have started with the intervention portion and finished on the control (standard therapy) arm, while Group B will have started with the control (standard therapy) arm and finished with the intervention arm.

We will quantify this endpoint as a longitudinal, repeated measure because it will evaluate for any change in peak knee extension between two time points, 12 weeks apart. It will be reported descriptively as mean +/- standard deviation, with significant differences across time points described using p-values

This secondary endpoint will be analyzed using a repeated measures ANOVA to evaluate the difference in peak knee extension between Initial and Final Outcome Assessments while controlling for order of intervention and control arms between Groups A and B.

9.4.4 Safety Analyses

The safety and feasibility endpoint will be evaluated as a tertiary objective. This endpoint will be analyzed as a summary of statistics during treatment to include a binary analysis of side effects (see Section 2.3.1.k) from intervention use and any report of adverse events (AEs). AEs will be coded in the participants medical record, counted once only for a given participant. We will present the severity, frequency and relationship of AEs to the intervention by System Organ Class (SOC) and preferred term groupings. We will report the start date, stop date, AE severity, relationship to intervention, expectedness, outcome and duration for each incident. If the AE leads to premature termination of study involvement, this will be recorded in the participants medical record along with reason for discontinuation and plan for follow-up.

9.4.5 Baseline Descriptive Statistics

Participants will be compared on baseline characteristics, including demographics and laboratory measurements, using descriptive statistics. The planned baseline descriptive statistics include the outcome measures as presented in Table 5.

9.4.6 Planned Interim Analyses

For interim analysis of the primary endpoint, change in peak knee extension at midstance as a result of the community use of the intervention, we will this parameter when we reach 50% of the estimated recruitment number. This will allow us to estimate the effect and the power.

9.4.7 Sub-Group Analyses

The primary endpoint will be analyzed in conjunction with the covariates age, weight/height and mobility (as characterized by functional scale GMFCS I-IV) using ANCOVA. Although the intervention is only in use for children, the age range is 3-17 years old which encompasses a broad range of developmental ability as well as a range of mobility based on severity of the participants movement disorder diagnosis. The same covariates will apply to the secondary endpoints.

9.4.8 Tabulation of individual Participant Data

Individual participant data will be listed by measure and time point. Average measures to evaluate each objective will be constructed based on individual data and presented as a representation of the entire cohort.

9.4.9 Tertiary/Exploratory Analyses

(1) *Effect of increasing dosage of intervention on primary endpoint*

The first tertiary endpoint is an assessment of the primary endpoint, change in peak knee extension during midstance, between multiple endpoints to investigate the effect of increasing dosage (i.e. time using the device in a training program). The time points include: Baseline Assessment to Initial Assessment (12 weeks), Baseline Assessment to Final Assessment (24 weeks) and Baseline Assessments to Follow-up Assessment (24 weeks device use + 6 weeks after completion).

(2) *Improvement in Knee Extensor and Flexor Muscle Spasticity*

The second tertiary endpoint is change in extensor and flexor muscle spasticity between two timepoints: Initial Assessment and Final Assessment, which correspond to the start and finish of the community use block. This endpoint will be measured using the Modified Ashworth and Tardieu Scales.

(3) *Improvement in Gross Motor Function*

The third tertiary endpoint is change in gross motor function between two timepoints: Initial Assessment and Final Assessment, which correspond to the start and finish of the community use block. This endpoint will be measured using two functional scales, the GMFM-66 and the PEDI-CAT.

(4) *Safety and Feasibility*

The fourth tertiary endpoint is safety and feasibility of the community intervention protocol within our target population. Occurrence of any adverse events will be recorded in the participants medical record at the time of occurrence, but will be reported in summary at the final visit as a measure of the safety of the study design. Feasibility will

be measured by compliance to the study procedures and whether or not each participant met the study dosage instructions. Each of these endpoints will be tracked through all study visits and procedures.

10 REGULATORY AND OPERATIONAL CONSIDERATIONS

10.1 Informed Consent Process

10.1.1 Consent/Assent Procedures and Documentation

A separate screening consent and assent will be used for the purpose of this study. The screening consent/assent will cover discussion of inclusion and exclusion criteria, review of pertinent medical records, a physical examination to include vital signs, height and weight measurements, and discussion of medical history, as well as kinematic measurements required for inclusion in the study (i.e. knee joint range of motion, ankle joint range of motion, hamstring contracture, foot-thigh angle, assessment of walking ability).

All participants will receive a verbal explanation in terms suited to their level of comprehension of the purposes, procedures and potential risks of the study and of their rights as research participants. Participants' guardians will have the opportunity to carefully review the written consent form and ask questions regarding this study prior to signing. Only one parent is required to provide parental permission. However, in cases of divorce where parents have joint legal custody for medical decisions for a child (e.g., by custody agreement or court order), both parents must give their permission regardless of the level of risk of the research. Exceptions may be made if one parent has since died, become incompetent, or is not reasonably available (e.g. incarcerated). If the second parent is unable to attend the consent process conference in person, the telephone/videoconference process will be used to obtain written consent from the second parent. Minor assent will be obtained where deemed appropriate by the clinician and the child's parent(s) or guardian, the child will also be included in all discussion about the trial and age appropriate language will be used to describe the procedures and tests involved in this study, along with the risk, discomforts and benefits of participation. Verbal assent will not be obtained in children younger than 6 because they typically do not have the ability to fully understand the nature of research. Children age 6-7 will provide verbal assent. Children between the age of 8 and 13 will be asked to sign the assent line on the separate assent form to indicate their assent. Children age 14-17 who are assessed by the principal investigator as having the capacity to understand the long form consent should sign the long form consent, using the signature line for assent. For those children who are age 14-17, for whom the principal investigator determines would not understand the long consent form, can sign the separate assent form. Assent will not be obtained for children older than 7 if the subject has an intellectual disability which precludes understanding the concept of voluntary assent so they cannot reasonably be expected to have the ability to provide meaningful assent due to their intellectual disability. Minor dissent will be considered to be refusal to participate after having the protocol explained (verbally, textually, and through demonstration by the consenting investigator), and after having all questions answered. If the minor provides dissent, enrollment in the protocol will not proceed. Dissent will be respected in children of all

ages. The original signed consent and assent forms will be placed in the medical record. The consent/assent procedure will take place in the motion capture lab at the NIH facility without interruption by other staff members to account for privacy.

10.1.2 Consent for minors when they reach the age of majority

When a pediatric subject reaches age 18, continued participation (including ongoing interactions with the subject or continued analysis of identifiable data) will require that consent be obtained from the now adult with the standard protocol consent document to ensure legally effective informed consent has been obtained. A legal guardian or representative may give consent for adult patients who do not have the capacity to give consent after the age of 18. Consent may be obtained either in person or over the phone. In person consent will proceed as described for the parents in the above paragraph. For the telephone consent process, we will provide the patient with the consent prior to discussing it, either by email scanned attachment, mail or fax. When sending by mail or fax, we will send two copies. After he or she has had an opportunity to review the consent, the investigator will contact the subject by telephone. At the time of discussion, we will use the consent document itself to discuss each section, confirm understanding and allow the participant to ask questions. If the subject chooses to continue to participate, the subject will sign and date one of the copies of the consent form. Signed consents will be returned by pre-paid return envelope. The Principal or Associate Investigator who led the discussion will sign and date and mail back one fully executed copy of the signed consent for the subject’s records. The original consent will be put into the NIH medical record. The informed consent process, including its time and date, will be documented on a progress note which will be entered into the NIH medical record. While waiting for the fully executed copy, the subject will be able to call the PI or refer back to the consent by either viewing the email scanned attachment or the 2nd copy that was sent by mail or fax, whichever is applicable.

If reconsent is not feasible, we request waiver of informed consent to continue to use data and/or specimens for those individuals who become lost to follow up or who have been taken off study prior to reaching the age of majority.

Requirements for Waiver of Consent consistent with 45 CFR 46.116(f)(3):

(1) The research involves no more than minimal risk to the subjects.
   a. Analysis of samples and data from this study involves no additional risks to subjects.

(2) The research could not practicably be carried out without the waiver or alteration.
   a. Considering the length of time between the minor’s last contact with the research team and their age of majority, it will likely be very difficult to locate them again. A significant reduction in the number of samples analyzed is likely to impact the quality of the research.

(3) As the research involves using identifiable private information or identifiable biospecimens, the research could not practicably be carried out without using such information or biospecimens in an identifiable format.

   a. Though the purpose of future studies cannot yet be known, they often involve the correlation of clinical outcomes and clinical interventions with laboratory studies. Such information would be unavailable if access to medical record numbers was unavailable.

(4) The waiver or alteration will not adversely affect the rights and welfare of the subjects.

   a. Retention of these samples or data does not affect the welfare of subjects.

(5) Whenever appropriate, the subjects will be provided with additional pertinent information after participation.

   a. We only request a waiver of consent for those subjects who have been lost to follow-up or who have been taken off study prior to reaching the age of majority.

10.1.3 Considerations for Consent of NIH staff, or family members of study team members

Consent for NIH staff will be obtained as detailed above with following additional protections:

Consent from staff members will be obtained by an individual independent of the staff member's team whenever possible. Otherwise, the consent procedure will be independently monitored by the CC Department of Bioethics Consultation Service in order to minimize the risk of undue pressure on the staff member.

10.1.4 Consent of Subjects who are, or become, decisional impaired

If a decisionally impaired minor were to reach the age of adult (18+ years old) during their participation in this study, then that subject would be re-consented according to the following procedure. The PI or AI will contact the NIH Ability to Consent Assessment Team (ACAT) for evaluation as needed for an independent assessment of whether an individual has the capacity to provide consent. If any participant is found not to have the capacity to consent at any point in the study, we will obtain consent from a legal guardian or other appointed representative; if this is not possible or cannot be obtained, the participant will not be enrolled, or will be withdrawn.

10.2 Study Discontinuation and Closure

This study may be temporarily suspended or prematurely terminated if there is sufficient reasonable cause. Written notification, documenting the reason for study suspension or termination, will be provided by the suspending or terminating party to study participants, investigator, and regulatory authorities. If the study is prematurely terminated or suspended, the Principal Investigator (PI) will promptly inform study participants, the Institutional Review Board (IRB), and sponsor and will provide the reason(s) for the termination or suspension. Study participants will be contacted, as applicable, and be informed of changes to study visit schedule.

Circumstances that may warrant termination or suspension include, but are not limited to:

- Determination of unexpected, significant, or unacceptable risk to participants

- Demonstration of efficacy that would warrant stopping
- Insufficient compliance to protocol requirements
- Data that are not sufficiently complete and/or evaluable
- Determination that the primary endpoint has been met
- Determination of futility

Study may resume once concerns about safety, protocol compliance, and data quality are addressed, and satisfy the sponsor, IRB and, as applicable, the Food and Drug Administration (FDA).

10.3 Confidentiality and Privacy:

Participant confidentiality and privacy is strictly held in trust by the participating investigators, their staff, and the sponsor(s). This confidentiality is extended to cover the clinical information relating to participants. Therefore, data, results, and all other information generated will be held in strict confidence. No information concerning the study results or the data will be released to any unauthorized third party without prior written approval of the sponsor.

All research activities will be conducted in as private a setting as possible. Following the introduction and consent/assent process at the start of visit 0, only relevant clinical staff, consisting of a licensed RMD physician and/or physical therapist, will remain with the patient and caregiver during the patient's physical examination. All other study staff will exit the exam area and return upon completion to resume study eligibility discussion. During all visits, the research lab will be scheduled and marked as occupied for the duration of the visit to avoid unnecessary personnel from entering the room.

Pictures and videos will be recorded of participants in this study in conjugation with motion analysis of gait. Participants can choose to sign an Authorization for Recording, Filming, and/or Photographing of Patients in the Clinical Center (see Appendix Document F) to give permission to use these videos for publications and public presentations of this work, with or without obscuring identifying features.

The study monitor, other authorized representatives of the sponsor, representatives of the Institutional Review Board (IRB), and/or regulatory agencies may inspect all documents and records required to be maintained by the investigator, including but not limited to, medical records (office, clinic, or hospital) and pharmacy records for the participants in this study. The clinical study site will permit access to such records.

The study participant's contact information will be securely stored at each clinical site for internal use during the study. At the end of the study, all records will continue to be kept in a secure location for 7 years after completion of the study as dictated by NIH policy.

Study participant research data, which is for purposes of statistical analysis and scientific reporting, will be stored at the NIH Clinical Center. This will not include the participant's contact or identifying information. Rather, individual participants and their research data will be identified by a unique study identification number assigned by the study team. The study data entry and study management systems used by clinical sites and by NIH Clinical Center research staff will be secured and password protected. At the end of the study, all study databases will be de-identified and archived at the NIH Clinical Center. Hard copies of data will be kept in locked storage which only study investigators have access to.

To further protect the privacy of study participants, a Certificate of Confidentiality has been issued by the National Institutes of Health (NIH). This certificate protects identifiable research information from forced disclosure. It allows the investigator and others who have access to research records to refuse to disclose identifying information on research participation in any civil, criminal, administrative, legislative, or other proceeding, whether at the federal, state, or local level. By protecting researchers and institutions from being compelled to disclose information that would identify research participants, Certificates of Confidentiality help achieve the research objectives and promote participation in studies by helping assure confidentiality and privacy to participants.

10.4 Future use of Stored Specimens and Data

The data collected from this study will be stored for future use for up to 7 years after completion and closing of the protocol. All data will be stored in a coded fashion. Codes to re-identify the data will be stored in password-protected computers as well as under hard copy in locked storage. The data collected as part of this study may be shared with collaborating laboratories at the NIH or outside of NIH; data sharing and transfer agreements will be executed if necessary. The de-identified data may be submitted to NIH-designated repositories and databases.

10.5 Safety Oversight

The Principal Investigator is responsible for data and safety monitoring for this protocol. Subjects will be informed that they can ask to stop the experiments at any time.

10.6 Clinical Monitoring

Clinical site monitoring is conducted to ensure that the rights and well-being of trial participants are protected, that the reported trial data are accurate, complete, and verifiable, and that the conduct of the trial is in compliance with the currently approved protocol/amendment(s), with International Conference on Harmonisation Good Clinical Practice (ICH GCP), and with applicable regulatory requirement(s).

Research study staff and the PI will meet on a regular basis when participants are actively receiving the intervention as part of the study protocol to discuss each participant. Study investigators will evaluate the safety of study participants during these meetings and also throughout the entirety of the protocol. Study investigators will respond to adverse events in a timely manner. The PI and study investigators will review any serious adverse outcomes and advise on whether or not changes in the research plan or procedures are necessary.

Additionally, NIH RMD Physician Dr. Katharine Alter will review all clinical data collected every 6 months to evaluate for patient safety issues.

10.7 Quality Assurance and Quality Control

The NIH CC's Quality Assurance Program will conduct study monitoring at least annually or more frequently. Monitoring visits will include a review of participant consent and assent documents, primary outcomes, and clinical notes which will be monitored for accuracy, correct dating, and agreement between documents. All regulatory reports, reviews and amendments, adverse events and problem reports related to the study, along with investigator credentials, training records and the delegation of responsibility log will also be reviewed during monitoring visits. Any major findings will be summarized in writing and reported to the study PI who will

be responsible for submitting the monitoring report to the IRB. Research study staff will perform internal quality management of study conduct, data collection, documentation and completion, throughout the study.

10.8 Data Handling and Record Keeping

10.8.1 Data Collection and Management Responsibilities

Biomechanical and electromyography data will be collected using appropriately calibrated computer-aided instrumentation and commercial software programs. Clinical scales of function and patient-reported outcome questionnaires will be recorded on data collection sheets, which will be stored in physical study binders in locked cabinets.

Upon enrollment, all participants will be assigned a subject code to be used as the patient identifier for all files and data collection sheets. Data will be stored securely using these assigned codes on password protected computers. Only study investigators will have access to the code key, which will be stored on the secure drive in a password protected file. All paper data collection sheets will be kept in de-identified (coded) participant folders in locked cabinets. Participant consent and assent forms and other medical records with names or patient identifiers will be kept in separate files in locked cabinets.

Data collection is the responsibility of the research staff under the supervision of the Principal Investigator. All study investigators are responsible for ensuring the accuracy, completeness, legibility, and timeliness of the data reported. All source documents will be completed in a neat, legible manner to ensure accurate interpretation of data.

Hardcopies of the study visit worksheets will be provided for use as source document worksheets for recording data for each participant enrolled in the study. Data recorded in the electronic case report form (eCRF) derived from source documents should be consistent with the data recorded on the source documents.

Clinical data (including adverse events (AEs) and concomitant medications) and clinical laboratory data will be entered into the secure Clinical Research Informatics System (CRIS) or Biomedical Translational Research Information System (BTRIS). All these data capture systems are provided by the NIH. The data system includes password protection and internal quality checks, such as automatic range checks, to identify data that appear inconsistent, incomplete, or inaccurate.

10.8.2 Study Records Retention

Study documents will be retained as per the NIH Intramural Records Retention schedule and requirements.

10.9 Protocol Deviations and Non-Compliance

It is the responsibility of the Principle Investigator (PI) to use continuous vigilance to identify and report deviations and/or non-compliance to the NIH Institutional Review Board as per Policy 801. All deviations must be addressed in study source documents. The PI is responsible for knowing and adhering to the reviewing IRB requirements.

10.9.1 NIH Definition of Protocol Deviation

A protocol deviation is any changed, divergence, or departure from the IRB-approved research protocol.

- Major deviations: Deviations from the IRB approved protocol that have, or may have the potential to, negatively impact the rights, welfare or safety of the subject, or to substantially negatively impact the scientific integrity or validity of the study.
- Minor deviations: Deviations that do not have the potential to negatively impact the rights, safety or welfare of subjects or others, or the scientific integrity or validity of the study.

10.10 Publication and Data Sharing Policy

10.10.1 Human Data Sharing Plan

This study will be conducted in accordance with the following publication and data sharing policies and regulations:

National Institutes of Health (NIH) Public Access Policy, which ensures that the public has access to the published results of NIH funded research. It requires scientists to submit final peer-reviewed journal manuscripts that arise from NIH funds to the digital archive PubMed Central upon acceptance for publication.

This study will comply with the NIH Data Sharing Policy and Policy on the Dissemination of NIH-Funded Clinical Trial Information and the Clinical Trials Registration and Results Information Submission rule. As such, this trial will be registered at ClinicalTrials.gov, and results information from this trial will be submitted to ClinicalTrials.gov. In addition, every attempt will be made to publish results in peer-reviewed journals. Data from this study may be requested from other researchers after the completion of the primary endpoint by contacting the Principal Investigator or his designee.

Data from this study may be shared with other NIH protocols, other investigators, database, or repositories under the following guidelines:

a) Data and samples may be shared with collaborating laboratories at NIH or submitted to NIH-designated repositories and databases. Submission to non-NIH sponsored or supported databases and repositories will be submitted for prospective IRB approval.
b) Data will be stripped of identifiers and may be coded ("de-identified") or unlinked from an identifying code ("anonymized") when sharing. When coded data is shared, the key to the code will not be provided to collaborators, but will remain at NIH.
c) Data may be shared with investigators and institutions outside of NIH with a Federalwide Assurance (FWA) or operating under the Declaration of Helsinki (DoH) following execution of a data sharing agreement between institutions.
d) Sharing with investigators without an FWA or not operating under the DoH will be submitted for prospective IRB approval.
e) Applicable identified data (e.g., generated via CRIS) will be shared following standard NIH CC operating procedures including BTRIS data access policies. Data to

be shared include baseline characteristics as well as key study outcome variables in a tabulated format, after adequate data cleaning, processing, and quality control.

10.11 Collaborative Agreements

Dr. Hao Su, PhD is an associate professor of mechanical and aerospace engineering at North Carolina State University. He will consult on analysis and interpretation of data. Specifically, he will assist in analyzing motion capture and electromyography data collected during walking with and without the exoskeleton intervention, as well as analyzing data from the onboard sensors of the exoskeleton itself to assist with evaluation of device performance and its effects on walking biomechanics. These data will be de-identified and he will not have a way to re-identify it. An applicable data transfer agreement will be executed. Dr. Su will not obtain consent.

10.11.1 Cooperative Agreements

There is a Cooperative Research and Development Agreement (CRADA) with the manufacturer of the exoskeleton device (Bionic Power) to be utilized in this study that was executed under an existing IRB protocol (#13-CC-0210). It is CRADA agreement #03240, and Amendment 1 to the original agreement is in force. Under the terms of the existing CRADA, Bionic Power supplies NIH with all equipment necessary to complete the intervention under the study protocol. Under this agreement, a maximum of 2 sets of exoskeleton hardware was provided.

We anticipate another CRADA agreement will be negotiated between the two parties under this protocol. Given the expanded number of devices required, the responsibilities of the parties are expected to shift such that some NIH IRP funds may be necessary to acquire exoskeleton hardware used for the intervention of under this protocol study.

10.12 Conflict of Interest Policy

Any actual conflict of interest of persons who have a role in the design, conduct, analysis, publication, or any aspect of this trial will be disclosed and managed. Furthermore, persons who have a perceived conflict of interest will be required to have such conflicts managed in a way that is appropriate to their participation in the design and conduct of this trial. The study leadership in conjunction with the Clinical Center has established policies and procedures for all study group members to disclose all conflicts of interest and will establish a mechanism for the management of all reported dualities of interest.

NIH guidelines on conflict of interest have been distributed to all investigators. There are no conflicts-of-interest to report.

11 ABBREVIATIONS

ACAT	Ability to Consent Assessment Team
AE	Adverse Event
AHA	Assisting Hand Assessment
ANCOVA	Analysis of Covariance
ANOVA	Analysis of Variance
BOTMP	Bruininks-Oseretsky Test of Motor Performance
BTRIS	Biomedical Translational Research Information System
BWS	Body Weight Support

CFR	Code of Federal Regulations
COC	Certificate of Confidentiality
CONSORT	Consolidated Standards of Reporting Trials
CP	Cerebral Palsy
CRADA	Cooperative Research and Development Agreement
CRF	Case Report Form
CRIS	Clinical Research Informatics System
DHHS	Department of Health and Human Services
DoH	Declaration of Helsinki
DSMB	Data Safety Monitoring Board
eCRF	Electronic Case Report Forms
EMG	Electromyography
FDA	Food and Drug Administration
FDAAA	Food and Drug Administration Amendments Act of 2007
FES	Functional Electrical Stimulation
FSR	Force Sensitive Resistor
FWA	Federal-Wide Assurance
GCP	Good Clinical Practice
GMFCS	Gross Motor Function Classification System
GMFM-66	Gross Motor Function Measure - 66
HIPAA	Health Insurance Portability and Accountability Act
ICH	International Conference on Harmonisation
IDE	Investigational Device Exemption
IRB	Institutional Review Board
IRS	Internal Revenue Service
iSCI	Incomplete Spinal Cord Injury
KAFO	Knee-Ankle-Foot Orthosis
LFE	Lateral Femoral Epicondyle
MD	Muscular Dystrophy
MRI	Medically Responsible Investigator
NIH	National Institutes of Health
NIH IC	NIH Institute or Center
NMES	Neuromuscular Electrical Stimulation
NSR	Non-Significant Risk
OHRP	Office for Human Research Protections
PEDI-CAT	Pediatric Evaluation of Disability Inventory-Computer Adaptive Test
PI	Principal Investigator
P.REX	Pediatric Robotic Exoskeleton
QA	Quality Assurance
QC	Quality Control
QUEST 2.0	Quebec User Evaluation of Satisfaction with assistive Technology 2.0
RMD	Rehabilitation Medicine Department
SAE	Serious Adverse Event
SB	Spina Bifida

SMO	Supramalleolar Orthosis
SOA	Schedule of Activities
SOC	System Organ Class
SOP	Standard Operating Procedure
TT	Treadmill Training
UP	Unanticipated Problem
US	United States
6MWT	6-Minute Walk Test

12 REFERENCES

Alotaibi, M., Long, T., Kennedy, E., & Bavishi, S. (2014). The efficacy of GMFM-88 and GMFM-66 to detect changes in gross motor function in children with cerebral palsy (CP): A literature review. *Disability and Rehabilitation, 36*(8), 617-627.

Binder, H., & Eng, G. (1989). Rehabilitation management of children with spastic diplegic cerebral palsy. *Archives of Physical Medicine and Rehabilitation, 70*(6), 482-489.

Bleyenheuft, Y., Arnould, C., Brandao, M., Bleyenheuft, C., & Gordon, A. M. (2015). Hand and Arm Bimanual Intensive Therapy Including Lower Extremity (HABIT-ILE) in Children With Unilateral Spastic Cerebral Palsy: A Randomized Trial. *Neurorehabilitation and Neural Repair, 29*(7), 645-657.

Booth, A. T., Buizer, A. I., Meyns, P., Oude Lansink, I. L., Steenbring, F., & van der Krogt, M. M. (2018). The efficacy of functional gait training in children and young adults with cerebral palsy: a systematic review and meta-analysis. *Developmental Medicine and Child Neurology, 60*(9), 866-883.

Bottos, M., & Gericke, C. (2003). Ambulatory capacity in cerebral palsy: prognostic criteria and consequences for intervention. *Developmental Medicine and Child Neurology, 45*(11), 786-790.

Bulea, T. C., Lerner, Z. F., & Damiano, D. L. (2018). Repeatability of EMG activity during exoskeleton assisted walking in children with cerebral palsy: Implications for real time adaptable control. *Proceedings of the Annual International Conference of the IEEE Engineering in Medicine and Biology Society, EMBS, 2018-July*, 2801-2804.

Bulea, T. C., Molazadeh, V., Thurston, M., & Damiano, D. L. (2022). Interleaved assistance and resistance for exoskeleton mediated gait training: Validation, feasibility and effects. *Proceedings of IEEE International Conference on Biomedical Robotics and Biomechatronics, BioRob*.

Bulea, T. C., Stanley, C. J., & Damiano, D. L. (2017). Part 2: Adaptation of Gait Kinematics in Unilateral Cerebral Palsy Demonstrates Preserved Independent Neural Control of Each Limb. *Frontiers in Human Neuroscience, 11*.

Chen, J., Hochstein, J., Kim, C., Tucker, L., Hammel, L. E., Daminao, D. L., & Bulea, T. C. (2021). A Pediatric Knee Exoskeleton With Real-Time Adaptive Control for Overground Walking in Ambulatory Individuals With Cerebral Palsy. *Frontiers in Robotics and AI, 8*, 173.

Damiano, D. (2006). Activity, Activity, Activity: Rethinking Our Physical Therapy Approach to Cerebral Palsy. *Physical Therapy, 86*(11), 1534-1540.

Damiano, D. L., Kelly, L., Vaugh, C., Westcott, S., & Lowes, L. (1995). Effects of quadriceps femoris muscle strengthening on crouch gait in children with spastic diplegia. *Physical Therapy, 75*(8), 658-671.

Damiano, D. L., Stanley, C. J., Bulea, T. C., & Park, H. S. (2017). Motor learning abilities are similar in hemiplegic cerebral palsy compared to controls as assessed by adaptation to unilateral leg-weighting during gait: Part I. *Frontiers in Human Neuroscience, 11*, 49.

Damiano, D. L., Stanley, C. J., Ohlrich, L., & Alter, K. (2017). Task-Specific and Functional Effects of Speed-Focused Elliptical or Motor-Assisted Cycle Training in Children with Bilateral Cerebral Palsy: Randomized Clinical Trial. *Neurorehabilitation and Neural Repair, 31*(8), 736-745.

Damiano, D., Prosser, L. A., Curatalo, L. A., & Alter, K. E. (2013). Muscle plasticity and ankle control after repetitive use of a functional electrical stimulation device for foot drop in cerebral palsy. *Neurorehabilitation and Neural Repair, 27*(3), 200-207.

Demers, L., Weiss-Lambrou, R., & Ska, B. (2002). The Quebec Use Evaluation of Satisfaction with Assistive Technology (QUEST 2.0): An overview and recent progress. *Technology and Disability, 14*, 101-105.

Dobkin, B., & Duncan, P. (2012). Should Body Weight–Supported Treadmill Training and Robotic-Assistive Steppers for Locomotor Training Trot Back to the Starting Gate? *Neurorehabilitation and Neural Repair, 26*(4), 308-317.

Dodd, K., Taylor, N., & Damiano, D. L. (2002). A systematic review of the effectiveness of strength-training programs for people with cerebral palsy. *Archives of Physical Medicine and Rehabilitation, 83*(8), 1157-1164.

Doglio, L., Pavan, E., Pernigotti, I., Petralia, P., Frigo, C., & Minetti, C. (2011). Early signs of gait deviation in Duchenne muscular dystrophy. *European Journal of Physical and Rehabilitation Medicine, 47*(4), 587-594.

Dreher, T., Vegvari, D., Wolf, S. I., Geisbüsch, A., Gantz, S., Wenz, W., & Braatz, F. (2012). Development of knee function after hamstring lengthening as a part of multilevel surgery in children with spastic diplegia: A long-term outcome study. *The Journal of Bone and Joint Surgery, 94*(2), 121-130.

Emara, H., El-Gohary, T., & Al-Johany, A. (2016). Effect of body-weight suspension training versus treadmill training on gross motor abilities of children with spastic diplegic cerebral palsy. *European Journal of Physical and Rehabilitation Medicine, 52*(3), 356-363.

Farris, R., Quintero, H. A., Murray, S. A., Ha, K. H., Hartigan, C., & Goldfarb, M. (2014). A preliminary assessment of legged mobility provided by a lower limb exoskeleton for persons with paraplegia. *IEEE transactions on neural systems and rehabilitation engineering : a publication of the IEEE Engineering in Medicine and Biology Society, 22*(3), 482-490.

Galey, S. A., Lerner, Z. F., Bulea, T. C., Zimbler, S., & Daminao, D. L. (2017). Effectiveness of surgical and non-surgical management of crouch gait in cerebral palsy: A systematic review. *Gait & Posture, 54*, 93-105.

Hanna, S. E., Bartlett, D. J., Rivard, L. M., & Russell, D. J. (2008). Reference curves for the Gross Motor Function Measure: percentiles for clinical description and tracking over time among children with cerebral palsy. *Physical Therapy, 88*(5), 596-607.

Hicks, J. L., Schwartz, M. H., Arnold, A. A., & Delp, S. L. (2008). Crouched postures reduce the capacity of muscles to extend the hip and knee during the single limb stance phase of gait. *Journal of Biomechanics, 41*(5), 960-967.

Hornby, T., Straube, D., Kinnaird, C., Holleran, C., Echauz, A., Rodriguez, K., . . . Narducci, E. (2011). Importance of specificity, amount, and intensity of locomotor training to improve ambulatory function in patients poststroke. *Topics in Stroke Rehabilitation, 18*(4), 293-307.

Jayaraman, A., Gregory, C., Bowden, M., Stevens, J., Shah, P., Behrman, A., & Vandenborne, K. (2006). Lower extremity skeletal muscle function in persons with incomplete spinal cord injury. *Spinal Cord, 44*(11), 680-687.

Kwon, S. P., Stanley, C. J., Kim, J., Kim, J., & Damiano, D. L. (2012). A Practical Strategy for sEMG-Based Knee Joint Moment Estimation During Gait and Its Validation in Individuals with Cerebral Palsy. *IEEE Transactions on Biomedical Engineering, 59*(5), 1480-1487.

Lefmann, S., Russo, R., & Hillier, S. (2017). The effectiveness of robotic-assisted gait training for paediatric gait disorders: Systematic review. *Journal of NeuroEngineering and Rehabilitation*.

Lerner, Z. F., Damiano, D. L., & Bulea, T. C. (2016). A robotic exoskeleton to treat crouch gait from cerebral palsy: Initial kinematic and neuromuscular evaluation. *Proceedings of the Annual International Conference of the IEEE Engineering in Medicine and Biology Society, EMBS, 2016-October*, 2214-2217.

Lerner, Z. F., Damiano, D. L., & Bulea, T. C. (2017a). A lower-extremity exoskeleton improves knee extension in children with crouch gait from cerebral palsy. *Science Translational Medicine, 9*(404).

Lerner, Z. F., Damiano, D. L., & Bulea, T. C. (2017b). The effects of exoskeleton assisted knee extension on lower-extremity gait kinematics, kinetics, and muscle activity in children with cerebral palsy. *Scientific Reports, 7*(1), 13512.

Lerner, Z. F., Damiano, D. L., Park, H. S., Gravunder, A. J., & Bulea, T. C. (2016). A Robotic Exoskeleton for Treatment of Crouch Gait in Children With Cerebral Palsy: Design and Initial Application. *IEEE transactions on neural systems and rehabilitation engineering : a publication of the IEEE Engineering in Medicine and Biology Society, 25*(6), 605-659.

Merino-Andrés, J., García de Mateos-López, A., Damiano, D. L., & Sánchez-Sierra, A. (2022). Effect of muscle strength training in children and adolescents with spastic cerebral palsy: A systematic review and meta-analysis. *Clinical Rehabilitation, 36*(1), 4-14.

Moen, T., Gryfakis, N., Dias, L., & Lemke, L. (2005). Crouched Gait in Myelomeningocele: A Comparison Between the Degree of Knee Flexion Contracture in the Clinical Examination and During Gait. *Journal of Pediatric Orthopaedics, 25*(5), 657-660.

Molnar, G. (1991). Rehabilitation in cerebral palsy. *Western Journal of Medicine, 154*(5), 569.

Moreau, N. G., Bodkin, A. W., Bjornson, K., Hobbs, A., Soileau, M., & Lahasky, K. (2016). Effectiveness of Rehabilitation Interventions to Improve Gait Speed in Children With Cerebral Palsy: Systematic Review and Meta-analysis. *Physical Therapy, 96*(12), 1938-1954.

Novak, I., Hines, M., Goldsmith, S., & Barclay, R. (2012). Clinical prognostic messages from a systematic review on cerebral palsy. *Pediatrics, 130*(5).

Novak, I., Morgan, C., Fahey, M., Finch-Edmondson, M., Galea, C., Hines, A., . . . Popat, H. (2020). State of the Evidence Traffic Lights 2019: Systematic Review of Interventions for Preventing and Treating Children with Cerebral Palsy. *Current Neurology and Neuroscience Reports, 20*(2).

Olney, S., MacPhail, H., Hedden, D., & Boyce, W. (1990). Work and power in hemiplegic cerebral palsy gait. *Physical Therapy, 70*(7), 431-438.

Perry, J. (1990). Pathologic gait. *Instructional Course Lectures, 39*, 325-331.

Postans, N. J., & Granat, M. H. (2005). Effect of functional electrical stimulation, applied during walking, on gait in spastic cerebral palsy. *Developmental Medicine and Child Neurology, 47*(1), 46-52.

Rethlefsen, S. A., Yasmeh, S., Wren, T. A., & Kay, R. M. (2013). Repeat hamstring lengthening for crouch gait in children with cerebral palsy. *Journal of Pediatric Orthopedics, 33*(5), 501-504.

Roemmich, R. T., & Bastian, A. J. (2018). Closing the loop: From motor neuroscience to neurorehabilitation. *Annual Review of Neuroscience, 41*, 415-429.

Rogozinski, B. M., Davids, J. R., Davis, R. B., Jameson, G. G., & Blackhurst, D. W. (2009). The efficacy of the floor-reaction ankle-foot orthosis in children with cerebral palsy. *Journal of Bone and Joint Surgery, 91*(10), 2440-2447.

Sandler, A. D. (2010). Children with spina bifida: key clinical issues. *Pediatric Clinics of North America, 57*(4), 879-892.

Sarajchi, M., Al-Hares, M. K., & Sirlantzis, K. (2021). Wearable Lower-Limb Exoskeleton for Children With Cerebral Palsy: A Systematic Review of Mechanical Design, Actuation Type, Control Strategy, and Clinical Evaluation. *IEEE transactions on neural systems and rehabilitation engineering : a publication of the IEEE Engineering in Medicine and Biology Society, 29*, 2695-2720.

Shideler, B. L., Bulea, T. C., Chen, J., Stanley, C. J., & Damiano, D. L. (2020). Toward a hybrid exoskeleton for crouch gait in children with cerebral palsy: neuromuscular electrical stimulation for improved knee extension. *Journal of NeuroEngineering and rehabilitation, 17*(1), 121.

Smith, P. A., Hassani, S., Reiners, K., Vogel, L. C., & Harris, G. F. (2004). Gait Analysis in Children and Adolescents With Spinal Cord Injuries. *The Journal of Spinal Cord Medicine, 27 Suppl 1*, 44-49.

Steel, K., Glover, J., & Spasoff, R. (1991). The motor control assessment: An instrument to measure motor control in physically disabled children. *Archives of Physical Medicine and Rehabilitation, 72*(8), 549-553.

Steele, K., Seth, A., Hicks, J., Schwartz, M., & Delp, S. (2010). Muscle contributions to support during single-limb stance in crouch gait. *Journal of Biomechanics, 43,* 2099-2105.

Thompson, N., Baker, R., Cosgrove, A., Corry, I., & Graham, H. (1998). Musculoskeletal modelling in determining the effect of botulinum toxin on the hamstrings of patients with crouch gait. *Developmental Medicine and Child Neurology, 40*(9), 622-625.

Tucker, L. A., Chen, J., Hammel, L., Damiano, D. L., & Bulea, T. C. (2020). An open source graphical user interface for wireless communication and operation of wearable robotic technology. *Journal of Rehabilitation and Assistive Technologies Engineering, 7*.

Van Der Krogt, M. M., Bregman, D. J., Wisse, M., Doorenbosch, C. A., Harlaar, J., & Collins, S. H. (2010). How crouch gait can dynamically induce stiff-knee gait. *Annals of Biomedical Engineering, 38*(4), 1593-1606.

Vuillermin, C., Rodda, J., Rtz, E., Shore, B., Smith, K., & Graham, H. (2011). Severe crouch gait in spastic diplegia can be prevented: a population-based study. *The Journal of Bone and Joint Surgery, 93*(12), 1670-1675.

Wein, H., Bryant, E., & Hicklin, T. (2017, September 12). *Robotic Device Aids Walking in Children with Cerebral Palsy*. Retrieved from NIH Research Matters: https://www.nih.gov/news-events/nih-research-matters/robotic-device-aids-walking-children-cerebral-palsy

Yeargin-Allsopp, M., Van Braun, K. N., Doernberg, N. S., Benedict, R. E., Kirby, R. S., & Durkin, M. S. (2008). Prevalence of Cerebral Palsy in 8-Year-Old Children in Three Areas of the United States in 2002: A Multisite Collaboration. *Pediatrics, 121*(3), 547-554.

Žarković, D., Šorfová, M., Tufano, J. J., Kutílek, P., Vítečková, S., Ravnik, D., . . . Otáhal, J. (2021). Gait changes following robot-assisted gait training in children with cerebral palsy. *Physiological Research, 70*(S3), S391-S408.

13 APPENDIX

A. FDA Letter: Study Determination for the Proposed Study titled, "Prototyped Powered Knee Orthosis" (January 2013)
B. FDA Letter: Study Determination for the Proposed Study titled, "Evaluating an Extension Assist Knee Ankle Foot Orthosis to Improve Gait in Children with Movement Disorders" (January 2019)
C. Recruitment Flyer
D. Pre-Screening Phone Script
E. Quebec User Evaluation of Satisfaction with assistive Technology (QUEST 2.0)
F. Authorization for Recording, Filming, and/or Photographing of Patients in the Clinical Center
